# In Vitro Evaluation of In Silico Screening Approaches in Search for Selective ACE2 Binding Chemical Probes

**DOI:** 10.3390/molecules27175400

**Published:** 2022-08-24

**Authors:** Alexey V. Rayevsky, Andrii S. Poturai, Iryna O. Kravets, Alexander E. Pashenko, Tatiana A. Borisova, Ganna M. Tolstanova, Dmitriy M. Volochnyuk, Petro O. Borysko, Olga B. Vadzyuk, Diana O. Alieksieieva, Yuliana Zabolotna, Olga Klimchuk, Dragos Horvath, Gilles Marcou, Sergey V. Ryabukhin, Alexandre Varnek

**Affiliations:** 1 Enamine Ltd., 78 Chervonotkatska Street, 02660 Kyiv, Ukraine; 2 Institute of Food Biotechnology and Genomics, National Academy of Sciences of Ukraine, 2a Osipovskogo Street, 04123 Kyiv, Ukraine; 3 Chemspace LLC, 85 Chervonotkatska Street, 02094 Kyiv, Ukraine; 4 Educational and Scientific Institute of High Technologies, Taras Shevchenko National University of Kyiv, 60 Volodymyrska Street, 01033 Kyiv, Ukraine; 5 Institute of Organic Chemistry, National Academy of Sciences of Ukraine, 5 Murmanska Street, 03028 Kyiv, Ukraine; 6 Palladin Institute of Biochemistry of the National Academy of Sciences of Ukraine, 9 Leontovitcha Street, 01054 Kyiv, Ukraine; 7 Laboratory of Chemoinformatics, University of Strasbourg, 4, rue B. Pascal, 67081 Strasbourg, France

**Keywords:** QSAR modeling, molecular docking, Neprilysin, angiotensin-converting enzyme, selective ACE2 enzyme binding, blood-brain barrier penetration, enzymology inhibition assay adjustment, in vitro studies

## Abstract

New models for ACE2 receptor binding, based on QSAR and docking algorithms were developed, using XRD structural data and ChEMBL 26 database hits as training sets. The selectivity of the potential ACE2-binding ligands towards Neprilysin (NEP) and ACE was evaluated. The Enamine screening collection (3.2 million compounds) was virtually screened according to the above models, in order to find possible ACE2-chemical probes, useful for the study of SARS-CoV2-induced neurological disorders. An enzymology inhibition assay for ACE2 was optimized, and the combined diversified set of predicted selective ACE2-binding molecules from QSAR modeling, docking, and ultrafast docking was screened in vitro. The in vitro hits included two novel chemotypes suitable for further optimization.

## 1. Introduction

Drug discovery is a tedious and time-consuming task, not able to cope with emergency situations such as the latest world-wide sanitary crisis due to the Severe Acute Respiratory Syndrome Coronavirus 2 (SARS-CoV-2) [1]. In order to be able to react quickly to such situations, mankind should develop an arsenal of drugs and probe molecules to interact with all relevant biological targets—even before these are discovered to be key players in infectious diseases.

So far, the life cycle of SARS-CoV-2 is reasonably well-understood thanks to such unprecedented worldwide collaborations. The list of key SARS-CoV-2-related targets is now available to the scientific community and waiting to be validated and used for drug development. One of them is angiotensin-converting enzyme 2 (ACE2)—a host zinc-containing metalloenzyme located on the surface of endothelial cells. SARS-CoV-2 enters cells via interaction between viral spike protein with extracellular domains of the transmembrane ACE2 that facilitates viral RNA entry accompanied by the ACE2 internalization [2]. Resulting ACE2 downregulation leads to the suppression of its highly important biological functions, causing hypertension, cardiac contractility, etc. [3]. Being highly expressed in lungs, vascular and intestinal cells, ACE2 is also thought to be indirectly responsible for the other systematic manifestations of SARS-CoV-2 side effects, caused by virus-induced changes in corresponding organs. Moreover, recent studies demonstrate an alarming correlation between the binding affinity of different strains of SARS-CoV-2 to ACE2 and the severity of induces symptoms. The Delta strain has multiple mutations in the spike protein that give it greater ACE2 receptor binding ability than the wild type [4], in contrast to the recent Omicron variant with much weaker binding affinity [5]. In addition, ACE2 produced by microvascular endothelial cells of the blood-brain barrier (BBB) potentially allows the virus to cross the BBB and exert neuroinvasive properties causing neurological diseases [6].

Despite the high importance of ACE2, there are very few ACE2-binder discovery projects in comparison with its homologue ACE [7]. Thus, there is still a need for understanding the molecular mechanisms of the ACE2 (SARS-CoV-2 related or not) and monitoring the potential influence of the temporary shutdown of ACE2 on the different biological tissues and organs. One of the tools that allow for achieving such understanding are chemical probes (further-probes)—small molecules that can induce desired biological effects by means of selective binding to specific molecular targets and thus allow researchers to ask mechanistic and phenotypic questions about the target in biochemical, cell-based or animal studies [8,9,10]. Up to now, the only report of ACE2-related probes development with experimental validation was a study describing two thiol-containing compounds, functioning as reducing agents and decreasing the binding of viral spike glycoprotein to ACE2 via allosteric modifications. However, instead of ACE2 they target spike disulfides of SARS-CoV-2, and thus cannot be used for investigation of the influence of ACE2 downregulation on the different biological processes without the presence of SARS-CoV-2 itself. Otherwise, there are rather few compounds (108, potential duplicates included—in 2020, at the time of this study) with reported ACE2 activity in the ChEMBL 26 database [11], 65 of which report thermodynamic inhibition constants K_i_. Herewith, ACE2 (CHEMBL3736) is one of the least well-explored biological targets so far.

Thus, here we aim to develop ACE2-targeting chemical probes to investigate the effects of a temporary shutdown of ACE2 on the different biological tissues and organs. The requirements for the high-quality small-molecule probes differ in comparison to the drug candidates. Probes do not need to exhibit good pharmacodynamics and oral bioavailability, but should demonstrate high potency and selectivity, which are not always expected from drugs (some of them have indeed the polypharmacological mode of action) [10]. In the context of current work, designed probes should show selectivity to ACE2 in comparison to its closely related homologous biological targets that play a role in counterbalancing the effect of ACE2 in blood pressure regulation– notably angiotensin-converting enzyme (ACE) and Neprilysin (NEP). Such selectivity would allow separation of the effects caused by ACE2 downregulation from the effects caused by the interaction of the probes with both closely related molecular targets (ACE and NEP). Moreover, the probes should be potentially blood-brain-barrier (BBB)-permeable for the SARS-CoV-2-induced neurological side effects investigation.

In the recently published work, the in silico identification of ACE2 probes via ultrafast docking resulted in 200 potential hits [12]. Now we have complemented our previous in silico screening results with almost 400 newly selected potential hits detected via classical docking, pharmacophore-based search, and QSAR ACE2/ACE and ACE2/NEP selectivity filtering with further experimental in vitro validation. The combined set of the most promising molecules, (overall 577 compounds from the Enamine screening collection), were tested in HTS mode in an enzymatic assay using the fluorescence method. Seven compounds were selected for dose-response assay and demonstrated micromolar activity. Those compounds represent novel ACE2-binding chemotypes that could be used for further structural optimization.

## 2. Methods

### 2.1. In Silico Screening

Two computational contributors to the final hit list contributed in addition to the already published ultrafast docking, vide supra: computational chemists of ENAMINE and the Chemoinformatics Laboratory at the University of Strasbourg (UNISTRA).

ENAMINE used the ICM docking tool to screen candidates against MD-refined geometries of the ACE2 active site, after removing the ligand and soaking in explicit water. Used site geometries were thus MD-visited ‘‘apo’’ structures with conserved crystallographic water molecules.

**Figure 1:**
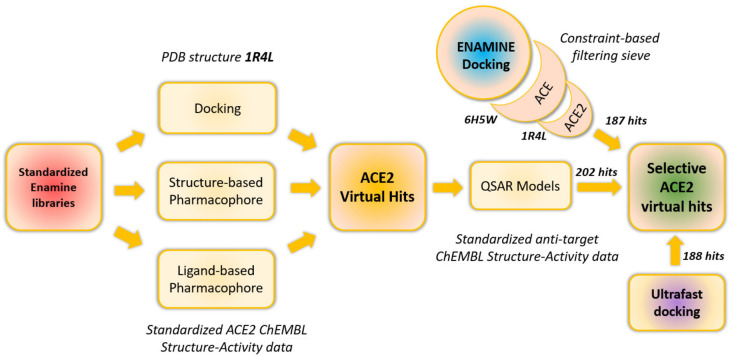
The general workflow of in silico search for selective ACE2 inhibitors. Except for “ENAMINE docking” and ultrafast docking, the rest of the modeling/screening steps were performed by UNISTRA.

UNISTRA (Figure 1) used structure-based pharmacophore screening with LigandScout [13], and docking with the PLANTS software, both based on analysis of existing native human ACE2 X-ray structure [14], (1R4L in PDB). In parallel, they used ligand-based approaches to create ACE2 ligand-based pharmacophores (LigandScout) [13] and to train ISIDA-descriptor [15] based QSAR classification models for ACE and NEP. These approaches are based on the known ACE2 (and respectively ACE and NEP) inhibitors from ChEMBL 26 database. QSAR models were designed to prioritize predicted ‘‘selective’’ ACE2 hits (Figure 1).

Finally, the most promising ACE2 virtual hits (together with the molecules selected from our previous work) [12] were subjected to in vitro validation.

#### 2.1.1. UNISTRA Datasets and Their Preliminary Processing

For structure-based modeling, the 1R4L ACE2-ligand cocrystal was downloaded from the Protein Data Bank and “washed” with the VegaZZ [16] program (crystallographic waters were removed, protein residues had their hydrogen atoms assigned according to default amino acid acidity/basicity, etc.). UNISTRA used the ChEMBL 26 database as a major input source of ACE2, ACE, and NEP reference ligands and training sets of reported activities. These were downloaded (into separate files) and then submitted to the group’s default standardization protocol on http://infochim.u-strasbg.fr/webserv/VSEngine.html (accessed in 10 October 2020). Returned standardized SMILES were then “deduplicated” with and respectively without considering stereochemical information.

For docking and ligand-based pharmacophore construction, only strict duplicates of the same stereoisomer/enantiomer were searched, specifically over the compound subsets with reported thermodynamic inhibition constants, Ki. If found, the stereoisomer was assigned as activity score the average value of the ChEMBL activity values for each entry if the latter were not conflicting. In terms of pKi values, specific entries were “not conflicting” if within ½ log unit with respect to each other. Otherwise, all the entries concerning that stereoisomer were discarded.

For categorical QSAR model building, used ISIDA fragment counts are stereochemistry-agnostic, hence structural duplicates were tracked with respect to the stereochemistry-depleted standardized SMILES strings (all stereoisomers now count as the same “structure”). This approach concerned the two “antitargets” ACE and NEP, for which the associated ChEMBL compound series were downloaded and standardized as above-mentioned. Categorical labels “active” and “inactive” were assigned to the compounds, following a procedure already used for automatic ChEMBL structure-activity data retrieval [17]. In particular, ACE is reported to have two distinct N- and C-terminal active sites, respectively. The Active/Inactive classification scheme used here does however not account for any specific site (data were merged irrespective of the—known or alleged—binding site). Duplicate SMILES associated with diverging activity classes were discarded.

#### 2.1.2. UNISTRA Docking

The UNISTRA group employed PLANTS [18] to perform docking based on the 1R4L PDB structure of the enzyme, by defining the center of the active site as the geometric center of the co-crystallized ligand, and employing a cutoff radius of 12 Å. The ChemPLP score was used at “speed1” (lowest speed–top accuracy). Previous experience with PLANTS/ChemPLP revealed that ChemPLP is compound size-dependent (larger molecules systematically become more negative scores) and that this dependence can best be empirically characterized by assuming ChemPLP proportional to N^0.35^ where N is the number of heavy atoms in the molecule. Therefore, docking hits were selected and ranked by a modified, size-independent ChemPLP × N*^−^*^0.35^ rather than the default ChemPLP.

#### 2.1.3. UNISTRA Ligand-Based Pharmacophore Model

Using LigandScout, the overlay of the 51 ChEMBL-reported ACE2 binders with pK_i_ or pIC50 better than 7 highlighted a cluster of 7 molecules, including species known to be selective for ACE2 over ACE. The pharmacophore extracted from the latter cluster was used in virtual screening, after manually pruning the Zn-chelating and anionic features (the latter thus figured as simple ‘‘hydrogen bond acceptors’’ in the pharmacophore). The simplified pharmacophore is likely less selective and thus prone to return more false negatives—but opens the possibility to discover binders with fewer charges and perhaps better availability and blood-brain barrier penetration).

#### 2.1.4. UNISTRA Structure-Based Pharmacophore Model

This was automatically extracted by LigandScout from the 1R4L PDB structure, and also pruned by removing the Zn-chelating and charged features (the cocrystalized ligand MLN-4760 contains two carboxylates rendered as simple hydrogen bond acceptors and a protonated secondary amino group rendered as H-bond donor.

#### 2.1.5. UNISTRA Antitarget Categorical QSAR Models

Structure-Activity Class QSAR models were built for ACE and NEP, following the standard evolutionary model building procedure [19] based on the Support Vector Machine learner (here—Support Vector Classifications) which includes choosing optimally suited descriptor spaces amongst one hundred proposed ISIDA fragmentation schemes. Top individual models were sampled by the evolutionary procedure and ranked by a fitness score reflecting the mean Balanced Accuracy BA_XV_ over a 12-fold repeated leave-1/3-out cross-validation scheme. The few best individual top models based on different ISIDA fragment descriptors were combined into consensus predictors for each antitarget. These eventually served as a final filter to refine (in the sense of rejecting those predicted as class “active” for either ACE or NEP) the initial list of potential ACE2 binders from pharmacophore screening and docking.

For all the ISIDA fragment count descriptor-based models, a “bounding box” criterion was employed as a simple applicability domain (AD) [20]—in order to be eligible for prediction, external compounds need to have fragment counts within the corresponding ranges defined by training set molecules.

#### 2.1.6. ENAMIN Docking into Multiple Active Site Conformers

Crystal structures of ACE and ACE2 proteins were derived from the RCSB databank. Structural alignment and sequence studies were performed using inbuilt ICM Molsoft modules [21] for bioinformatical tasks. The Gromacs [22] 2019 package was used for conducting MD and further analysis of the results. In the present work, we used a full-atom amber99 force field for energy minimization of the protein-ligand complexes, namely a descent algorithm for 20,000 steps [23]. Equilibration of the solvent was carried out with positional restraints superimposed on the atoms of protein structures, while the solvent molecules remained mobile for all the simulated 100 ps. Each system was placed in a box where the layer of TIP3P water molecule was 10 Å. Final systems were neutralized by adding of Na^+^ and Cl*^−^* ions to achieve a concentration of 150 mM. All simulations were performed in periodic boundary conditions using the v-rescale thermostat algorithm to maintain the temperature (310 K) and the Parrinello-Rahman barostat algorithm for permanent pressure constant (1 bar) [24,25]. The long-range non-bonded interactions were calculated using the Particle-Mesh-Ewald method [26]. Ligand topologies were generated with an antechamber module from the AmberTools18 package. ACE2- and ACE-ligand bound forms (PDBID: 1R4L and 6H5W), as well as the apo-forms of ACE2 and ACE, the same ligand-bound complexes with extracted ligands, were subjected to MD simulations of 150 ns. Conformational clustering of the trajectories was performed with Gromacs tools based on the root mean square values calculated for Cα atoms and a cut-off distance between clusters of 0.15 nm.

For docking, selected protein conformers from the MD simulations were prepared as active sites for the ICM docking software. The grid maps were generated in a rectangular box with 0.5 Å grid spacing centered at the ligand binding site. Both, the reference set and Enamine screening collection were docked in a Batch Docking mode with simultaneous generation of 3D conformers, from the spreadsheet and the previously indexed database, respectively. Flexible ligand docking with the ICM software uses Monte Carlo simulations to globally optimize a set of ligand internal coordinates in the space of grid potential maps calculated for the protein pocket [27]. A thoroughness value, which represents the length of the simulation was increased up to three, as the pocket contains metal ions.

Ligand poses were scored and then ranked according to the ICM scoring function. The quality of each complex was estimated based on the predicted score, binding energy function, which is calculated as the weighted sum of ligand-target van der Waals interactions and internal force field energy of the ligand, free energy changes due to conformational energy loss upon ligand binding, H-bonding interactions, H-bond donor-acceptor desolvation energy, solvation electrostatic energy upon ligand binding, hydrophobic free energy gain and a size correction term proportional to the number of ligand atoms [28].

### 2.2. Experimental Methods

Despite the commercial availability of ACE2 Inhibitor Screening Kits the optimal HTS protocols are still not published and their validation is still needed. Among available kits the Abcam, ab273373 was used [29]. This assay is based on the ability of active angiotensin-converting enzyme (ACE2) to cleave a synthetic MCA-based peptide substrate (the structure not disclosed by the manufacturer) and release a free fluorophore. The released fluorophore can be easily quantified using a standard fluorescence microplate reader. During this screening campaign, the next milestones were estimated: (i) optimization of the test compounds solutions used volume (without loss of performance and analytical signal recognition); (ii) optimization of reaction volume and concentrations of the reagents; (iii) determination of IC_50_ for known inhibitor MLN-4760 (EN300-22203532) and reference inhibitor from the kit (the structure not disclosed by manufacturer) in new validated conditions; (iv) primary screening of 577 selected compounds; (v) hit confirmation; (vi) dose-response curve (DRC) of confirmed hits.

At the volume optimization step, 25 and 40 µL samples were tested in 384-well plates in kinetic mode (Exc/Em 320/420 nm). The results showed that the asay demonstrates good performance: Z-prime values calculated from rate values were in the range of 0.6–0.8, S/B values calculated from RFU were 25–30 for 25 µL and 40–50 for 40 µL. Therefore, the smaller volume (25 µL) was chosen for further optimization. In addition, it should be noted, that the reaction rate values decreased during incubation time, which could be explained by a slight increase in temperature from 25 °C at the 10-min mark to 27 °C at 70 min (see Figures S1 and S2 and Table S7 in the Supplementary Materials). The next optimization step was to optimize dilutions of enzyme and substrate without significant decreasing of the analytical signal. The 1:2, 1:4, and 1:8 dilutions of both enzyme and substrate were used (see Figures S3–S6 in the Supplementary Materials). The data is summarized in Table 1. The analytical signal quality in all dilutions meets the requirements of HTS (Z-prime > 0.5), but at low enzyme or substrate concentrations (S/B) values decrease significantly. Consequently, it is preferable to choose dilutions, which do not exceed 1:4 for both enzyme and substrate. In this study, the 1:2 dilution of enzyme and substrate was used for further experimentations.

In optimized conditions, the DRC for two reference compounds was measured (three times each, see SI), and it should be noted that IC50 for MLN-4760 measured under chosen conditions differs by a factor of 2 to 6 from PubChem data (440 pM). In the case of inhibitor provided by the Manufacturer (structure not disclosed), IC50 measured also differs by a factor of 5 to 9 from the value specified in the description to the kit. For the primary screen, the IC50 values for the inhibitor provided by the manufacturer, IC50 = 185–300 nM, and MLN-4760, IC50 0.9–2.5 nM were used.

The commercial Abcam (ab273373) Angiotensin II Converting Enzyme (ACE2) Inhibitor Screening Kit and 384-well 3544 Corning low volume, black clear bottom plates were used. The reagent preparation was performed in accord with kit instruction. In order to prepare the required enzyme concentration, we dissolved 20 µL of stock enzyme solution (concentration in stock and MW not disclosed) in 198 µL of ACE2 Dilution Buffer (provided by the Manufacturer), then aliquoted the entire volume into 25 µL and frozen at −70 °C. A substrate solution calculated for one assay well contained 0.5 µL of stock Substrate solution and 9.5 µL of ACE2 Assay Buffer, stored at −70 °C. Reference inhibitor from the kit (five µL in stock, structure, and MW not disclosed) was not aliquoted, 50 µL of ACE Assay Buffer should be added directly before the experiment.

Primary screen assay conditions. **Volume:** 25 µL; **Concentrations:** enzyme and substrate solutions prepared according to the instructions and further diluted both to 1:2 in the Assay mixture. **Assay buffer:** provided by the Manufacturer (composition not disclosed, see SI for reference). **Compounds:** 20 µM final concentrations in the Assay mixture, *n* = 1. **Reference Inhibitors:** (1) Ref. inhibitor provided by the Manufacturer, 300 nM, (*n* = 4); (2) Compound from Enamine library EN300-22203532, 2.5 nM, (*n* = 4). **Temperature:** 25 °C. The enzyme solution was added into corresponding plate wells (columns 1*–*22), and buffer was added into wells corresponding to negative (Enzyme) control (columns 23, 24). Plates were incubated for 15 min at 25 °C. Then Substrate solution was added to all experimental wells. Plates were centrifuged and measurement started in kinetic mode (Exc/Em 320/420 nm). **Results processing:** two time points were chosen from kinetic curves for all wells and corresponding RFU values were obtained. ΔRFU/Δt were calculated for each curve and % of inhibition values were calculated from the equation: 100%—ΔRFU of sample/ΔRFU of Enzyme control*100%, according to the recommendation of the Manufacturer.

DRC and IC50 calculations. **Concentrations:** enzyme and substrate solutions were diluted both to 1:2 in the Assay mixture. **Assay buffer:** provided by the Manufacturer (composition not disclosed). **Temperature:** 25 °C. **Compounds:** 2-fold titrated from 100 µM, *n* = 4. **Reference Inhibitors:** (1) Ref. inhibitor Abcam 2-fold titrated from 1.5 µM, (*n* = 4); (2) Ref. inhibitor EN300-22203532, 2-fold titrated from 20 nM, (*n* = 4). Compounds were incubated with enzyme for 15 min. Plates were measured in kinetic mode (Exc/Em 320/420 nm). **Results processing:** two time points were chosen from kinetic curves for all wells and corresponding RFU values were obtained. ΔRFU/Δt were calculated for each curve and percent of inhibition values were calculated from the equation: 100%—ΔRFU of sample*/*ΔRFU of Enzyme control × 100%, according to the recommendation of the Manufacturer. Dose-response curves were fitted using GraphPad Prism using sigmoidal fit with variable slope. It should be noted that reference compound EN300-22203532 did not fit the proper titration range and demonstrated lower IC50 value than previously.

## 3. Results

### 3.1. General Discussion of So-Far Known ACE2 Inhibitors

Interrogation of the ChEMBL 26 database concerning human ACE2 (CHEMBL3736) returned 102 compounds for which 108 test results are available, whereas human ACE (CHEMBL1808) is linked to 1032 compounds associated with 1346 test results. Human Neprilysin (CHEMBL1944) relates to 673 compounds and 806 test results. Clearly, ACE2 is much less studied than the two other mentioned enzymes. The sparsity of ACE2 data (after standardization and duplicate removal, 60 entries would be basically available for structure-activity modeling) is therefore limiting the usefulness of any ACE2-activity predictor trained by machine learning on so little data. A Support Vector Machine-based regression model predicting ACE2 *pK_i_* values can actually be fitted and cross-validated (Q^2^ = 0.87) on the few ChEMBL entries, but its applicability domain is forcibly restricted to the ChEMBL-covered chemical families. Therefore, this approach was not actually used in virtual screening.

**Figure 2:**
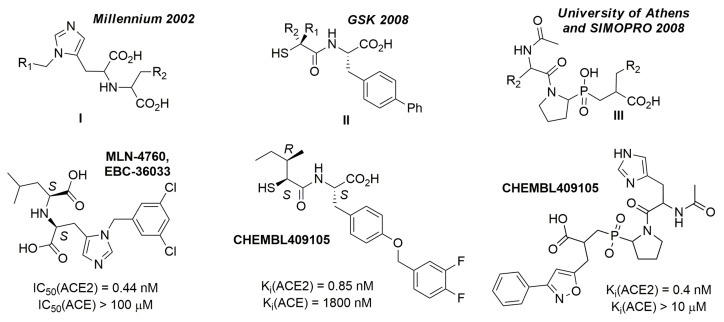
Main chemotypes of known selective ACE2 inhibitors with corresponding chemotypes (I–III) and showcasing examples.

Furthermore, there is no way to learn ACE2/ACE or, respectively, ACE2/NEP selectivity directly, by considering ligands tested on both the concerned enzymes and for which experimental activity values could be converted into an experimental selectivity score. Therefore, the ‘‘selectivity’’ issue was simply addressed by building QSAR predictors based on the rather large ACE and NEP datasets, in order to preferentially select candidates predicted not to bind by these models. A few examples of selective ACE2 binders include CHEMBL429844 (synonyms: MLN-4760, EBC-36033, EN300-22203532 (0.44 nM)), CHEMBL251808 (0.85 nM.) and CHEMBL409105 (0.4 nM) [3,30]. Detailed analysis showed that all these compounds come from three independent projects and are based on three major chemotypes. The first chemotype is a histidine-based polypeptide mimetic (I) developed by Millennium Pharmaceuticals in 2002 [31], the second one is an α-thiol amide-based compound (II), proposed by GlaxoSmithKline in 2008 [3], and the last one is pseudoproline containing phosphinic peptides (III), which comes from an academic collaboration between the University of Athens and SIMOPRO [30] (Figure 2). Moreover, all these chemotypes show selectivity to ACE2 over ACE and contain highly polar chemical moieties such as carboxylates, which likely hinder the blood-brain barrier (BBB) passage. Consequently, the existing selective ACE2 inhibitors cannot be used for in vivo investigations of the SARS-CoV-2 neuroinvasive properties and related side effects. It is important to note that passive BBB-permeability (which does not involve active transportation by transport proteins) can be predicted using simple empirical rules: molecular weight ≤ 400 Da, the total number of forming H-bonds < 8, lipophilicity: 1.5 ≤ LogP ≤ 2.7, Polar surface area (PSA): 60–70 Å [2,32,33].

The binding site in ACE2 has multiple hydrogen bonding spots, a zinc-chelation spot, and hydrophobic interaction sites. The ligand MLN-4760 acts as a transition state analogue, mimicking a peptide substrate, anchored with a C-terminal carboxylate moiety and coordinated by the catalytic zinc. The overall grade of homology between ACE2 and ACE domains is about 32% sequence identity, while the ACE2 peptidase domain has a 42% sequence identity (or 75% similarity) and a 42% sequence identity (or 76% similarity) with the individual N- and C-domains of ACE, respectively.

### 3.2. UNISTRA Pharmacophore-Based Screening

**Figure 3:**
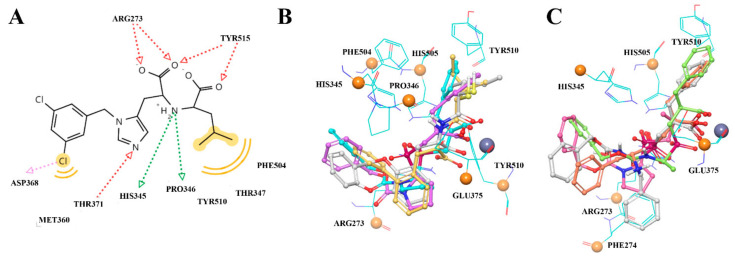
The interaction between the MLN-4760 and ACE2 (Zn^2+^—ligand interactions and charged features excluded) (**A**): interaction map, (**B**) the most active compounds from the reference set fitting the pharmacophore model; (**C**) less active substances possessing distinct conformations and interaction network.

**Figure 4:**
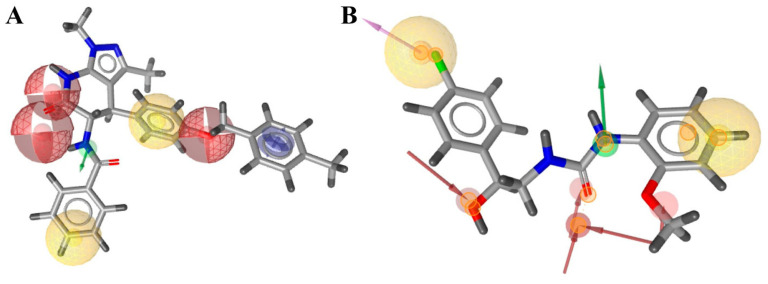
The LB (left, **A**) and the SB (right, **B**) pharmacophores. Red-colored spheres—H-bond acceptor features, green—H-bond donor features, yellow—lipophilic features, blue—aromatic ring.

The structure-based pharmacophore was built according to the interaction diagram (Figure 3), using the 1R4L crystal structure of ACE2 with its co-crystallized ligand CHEMBL429844. Ten features are present in the pruned SB pharmacophore (Figure 4B; as mentioned in Methods, charge and Zn chelating features were removed). To increase the probability of finding hits, any one out of 10 features was optional for screening (i.e., could be ignored when evaluating results), which means any compound from the Enamine screening library with at least nine matched features was retrieved (72 hits).

For ligand-based pharmacophore modeling, a total of 51 compounds with *pK_i_* values >7 (35 of them have *pK_i_* > 8). Their LigandScout clustering led to seven distinct clusters; three of these had more than three members and represented the main chemotypes of known ligands. The chosen cluster consisted of seven compounds representing only ACE2-selective compounds. Herewith, the ligand-based pharmacophore (Figure 4A) includes seven features—the minimum number of features presented in all cluster members, which allowed to separate the selective cluster from the full list of known ACE2-inhibiting compounds. It retrieved 91 hits from the Enamine screening library.

### 3.3. UNISTRA Docking Results

Prior to actual virtual screening, the PLANTS docking protocol was first challenged to “discover” the ChEMBL-reported active ACE2 compounds, at *pK_i_* > 7. Unfortunately, this question cannot be settled on the basis of ChEMBL data only: out of the ChEMBL-extracted 61 ACE2 ligands of known thermodynamic inhibition constant, 41 have *pK_i_* > 7 (34 have *pK_i_* > 8) and no docking program can capture the subtle differences in interactions between these and the remaining micromolar compounds. However, if this set is augmented with 200 random ChEMBL decoy molecules, ACE2 binders are seen to be top-ranked by PLANTS, with a ROC AUC value of 0.71 for the ChemPLP scoring function used. Ranking by ligand efficacy (ChemPLP/N—number of ligand heavy atoms) is less discriminant (ROC AUC = 0.59). However, ranking by the size-independent, normalized ChemPLP × N^−0.35^ criterion actually improves discrimination (ROC AUC of 0.73). The coefficient 0.35 has been determined independently, based on the observation of the UNISTRA team that, over multiple targets, ChemPLP scores appear to be significantly correlated to the number of ligand heavy atoms, according to a logarithmic law log (−ChemPLP) = 0.35 log (N)^+^ constant. Docking hits from the Enamine screening collection were defined as compounds that simultaneously outperform the native 1R4L ligand in terms of all three scores ChemPLP, ChemPLP/N, and ChemPLP × N^−0.35^. A total of 245 compounds fulfill these conditions.

### 3.4. UNISTRA QSAR Modeling Results

**Figure 5:**
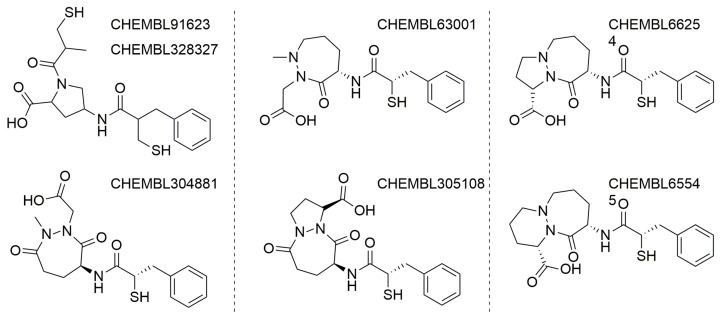
Example of strongly congeneric ACE actives from CHEMBL.

While there is a priori plenty of data to support the calibration of an ACE binding predictor model, the major issue here was related to the specific two-domain structure of the ACE target. The literature analysis showed that despite the almost identical structure of these domains, their activity differs. For some ligands, this domain-specific difference reaches more than 2 logarithmic units of concentration [34]. In most primary sources, the authors do not specify which binding site the given activities belong to [3,35], so the construction of a qualitative regression model for ACE was almost impossible. Building different models for each ACE active site is not an option—mainly, because as above-mentioned that site is not always clearly assigned. The used classification scheme, however—albeit arguably not rigorous—nevertheless allows, given the empirically chosen threshold of 25 nM defining “actives”, a near-perfect separation of classes (cross-validated Balanced Accuracy = 0.97). Near-perfect separation levels typically mean that the problem was “too easy”—the pool of actives being typically represented by a homogeneous series of actives, for which the machine learning may easily find some specific signature in terms of descriptors. The ACE model basically learned to recognize the specific signature of the most “popular” ChEMBL-reported ACE actives –in the present context, being classified as “inactive” by the ACE model basically means that the candidate is not a member of the structural families of top ACE binders (see Figure 5.), all while containing substructures seen in ACE-tested compounds (in order to be considered inside the model AD).

The ACE model is publicly available on the Strasbourg web server http://infochim.u-strasbg.fr/webserv/VSEngine.html (accessed on 1 January 2022). It is a consensus Support Vector Classification model combining four individual “recipes”, and returns the mean of the four real-value scores, denoting the predicted fuzzy truth value of the hypothesis that the compound is “active”. Three models are based on circular default (atom symbol) and respectively Force-Field colored ISIDA fragment counts, the fourth uses Force-Field colored ISIDA sequence counts.

For the NEP compounds, the automated extraction and curation of CHEMBL1944-associated structure-activity data resulted in a pool of 340 unique (stereochemistry-depleted) structures, which, at cut-off of 50 nM, result in a near-perfectly balanced set in terms of actives/inactives. The resulting NEP model, also available on the above-mentioned web server, is a consensus of five individual ‘‘recipes’’. Three of these involve again circular default (atom symbol) and respectively Force-Field colored ISIDA fragment counts, one uses Force-Field colored ISIDA sequence counts and the last utilizes pharmacophore-colored ISIDA circular fragments. Their cross-validated Balanced Accuracies are also very high—between 0.86 and 0.91.

Above QSAR models were applied to assess ACE and NEP binding propensity of the UNISTRA diverse hits from docking. The web-supported ACE model implicitly provides a trustworthiness assessment combining the applicability of each of the four individual models (by the “bounding box” criterion) and the level of agreement of their individual predictions. The docked hits were mostly well covered by the ACE approach (>80% of predictions were ranked “good” or “optimal” in terms of trustworthiness). A number of 85 of the 234 submitted docking hits were predicted to likely be active (“active” hypothesis fuzzily true at >70%). As for the NEP predictor, ~60% of submitted docking hits were ranked “good” or “optimal” in terms of trustworthiness, but only one molecule had its predicted “active” hypothesis fuzzily true at >70%).

Predicted ACE and NEP binders were, however, at this preliminary phase of the project, not actually discarded—rather, they were allowed to enter the ACE2 experimental testing, in view of a future testing campaign on ACE and NEP, respectively. In further iterations of this project, as soon as the antitarget ACE and NEP models will be validated by the planned testing campaigns (or rebuilt in view of additional experimental data), predicted “actives” will be discarded. Note that the very stringent (high nanomolar) cut-off criteria to define antitarget actives should, in perspective, be lowered—the current models would correctly rank low micromolars as antitarget-inactive, all while at a similar affinity for ACE2 they would count as “hits”.

### 3.5. ENAMINE MD-Based Docking Results

Both ACE- and ACE2-ligand complexes showed notable stability, without any significant changes in the side chain orientation during the MD simulation. The problem is that despite the overall difference between the sequence the binding sites are similar enough. From the study of all available complexes we found, that the ACE binding site contains several water molecules, which are absent in ACE2 complexes. In an attempt to use ACE as an anti-target molecule we decided to determine how stable these protein-bound water molecules are.

**Figure 6:**
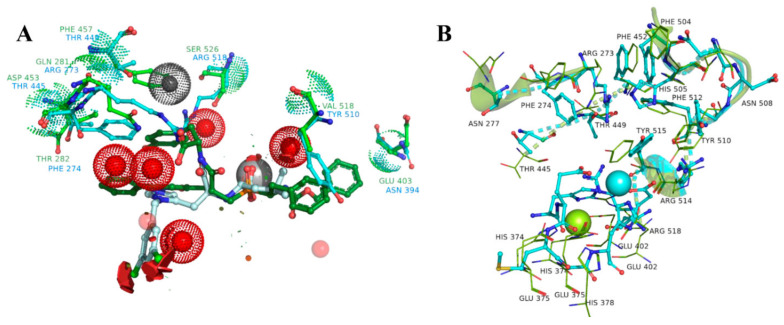
A Cα-based superposition of two binding sites, with highlighted the most distinct amino acids involved in the ligand binding (green color for ACE and cyan for ACE2) (**A**). It can be seen, from the spatial alignment, that water molecules, depicted as steric features (red spheres) and found in all resolved ACE structures, would interfere with moieties of the ACE2-selective ligand from the ACE2 catalytic site (1R4L). An MD simulation of the apo-ACE2 form showed a moderate amino acid motility inside the active site, despite the overall rearrangements of the protein structure, when the cleft extended-initial state is colored with cyan, and the final state with green (**B**).

From these, we processed the apo-forms of ACE and ACE2 with coordinated zinc atoms in the binding sites by means of 150 ns MD simulation. After 50 ns, the binding site of ACE2 in the absence of the ligand started to extend the cleft and finally shared a common geometry with ACE2 from PDB ID: 1R42. During the first 50 ns of the trajectory the atoms of the binding site preserved its initial coordinates and then we observed a progressive extension of the binding cleft, which stopped after 120 ns, but there were no significant changes in the ligand interacting residues. The structural data was also confirmed with a conformational clustering. Based on the matrix of RMS deviation values (atom-pair distances method) calculated for the atoms of the binding site region (radius of 10 A from its center), we defined three main clusters, which corresponded to these conformational transitions. A similar situation occurred with the ACE apo-form protein structure. However, the protein-bound water molecules, which were found in all ACE crystal structures, did not change their positions across the MD simulations (Figure 6).

Finally, we generated docking models using the final states of both ACE and ACE2 protein-ligand trajectories. And solvent molecules from ACE served as positional constraint features to prevent simultaneous interaction of inhibitors with both ACE2 and ACE binding sites.

**Figure 7:**
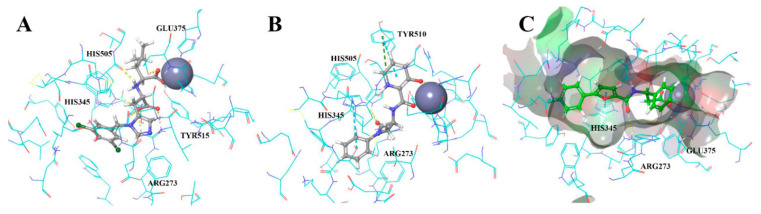
(**A**) Cocrystalized MLN-4760 redocked. (**B**,**C**) Similar poses and interaction maps illustrated by two highly scored compounds (Z3488516360 and Z85905794, Table S5 in the Supplementary Materials) from the virtual screening resulting set.

We applied docking to predict stable conformations and binding orientation of ligands inside the binding site of the ACE2 protein without consideration of the reported activity values [36]. Further analysis of the ACE2-ligand interactions revealed that the most adequate docking model for ACE2, assumes Pi-Pi-stacking interactions for one of His345, His505, and Tyr510 residues; formation of a hydrogen bonds with Arg273; hydrogen bonds with Tyr515 or Glh375; geometrically available electron donor near the metal atom (Figure 7). Our reference set combined only compounds with estimated Ki values from ACE2 and ACE test results (52 and 120 assays, respectively). The molecular docking of the reference compounds against ACE and ACE2 resulted in a division of the reference set by target selectivity. The list with scores is given in the SI. Finally, 39 out of 52 ACE2-selective reference inhibitors with known Ki values got into the corresponding binding site.

The molecular docking of the compounds with known activity against ACE returned 166 compounds with calculated scoring function values similar to that found in the literature.

### 3.6. In Vitro ACE2 Screening

The combination of pharmacophore (202 molecules) and docking (187 molecules) approaches resulted in a set of 389 compounds, which were selected from the Enamine compounds library for testing via enzymatic fluorescence assay in high throughput screening mode. In addition, 188 compounds were selected for testing from the results of our previous computational modelling paper [12]. After stock quantity and QC checks, 577 compounds were subjected to the in vitro phase.

**Figure 8:**
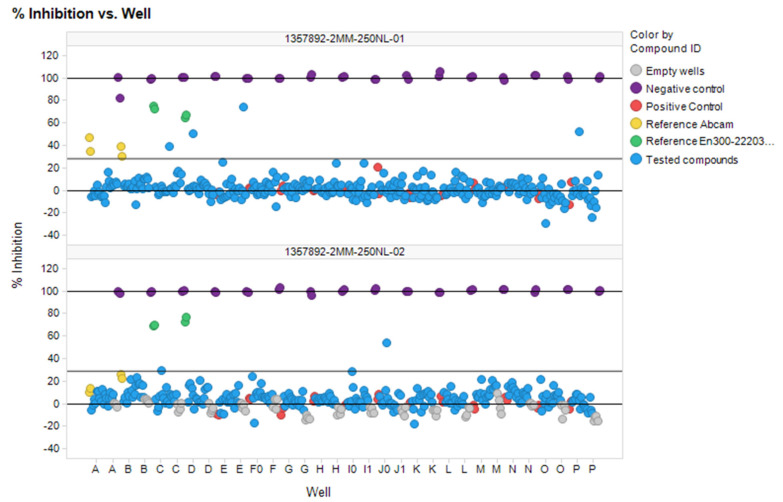
Plate scatterplot (% inhibition) for 577 compounds screened against ACE2 in two plates.

Using optimized conditions (384-well plates, 25 µL, 1:2 enzyme and substrate dilution, 20 µM final concentrations of investigated compounds) the 577 selected compounds were screened. The results of this screening in two plates are shown in Figure 8. Both plates showed good analytical signal (Z″ = 0.84 and 0.82 and S/B = 5.54 and 5.2 respectively).

The seven compounds out of 577 screened were selected according to to hit criteria Inh% > 3 × SD + Avg, which equals to 28.2 (first plate) and 28.8 (second plate). Five out of seven compounds had shown high intrinsic fluorescence on wavelengths 320 exc/420 em, but this feature did not interfere with calculations of % inhibition because ΔRFU/Δt parameter was used. Analytical signal parameters met HTS requirements in the given conditions (Z-prime < 0.5).

**Figure 9:**
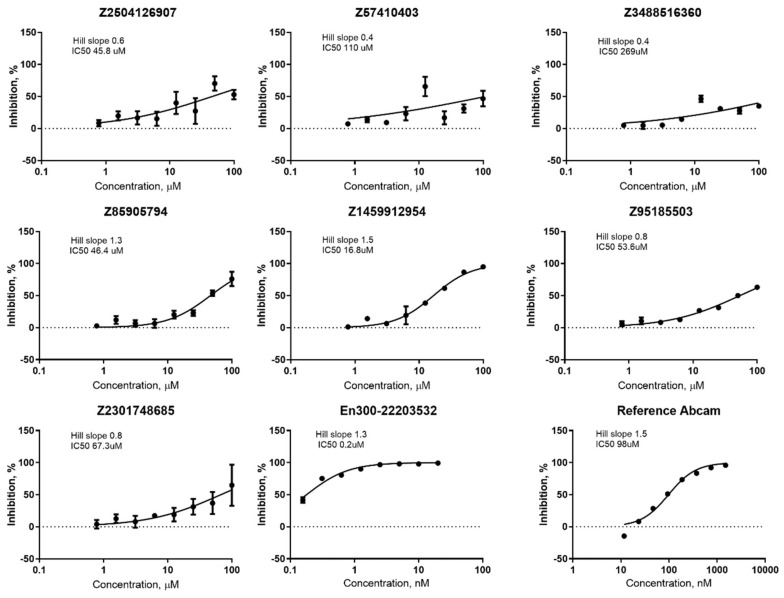
DRC for seven hits and two reference compounds.

All identified hits were confirmed and all the hit compounds corresponding DRC were measured (Figure 9). IC50 values for chosen compounds + two reference compounds were calculated from dose-response curves. In general, all the studied compounds appeared to have low potency to inhibit ACE2, beside ID# Z1459912954, which corresponds to an IC50 value of 16.8 µM.

**Figure 10:**
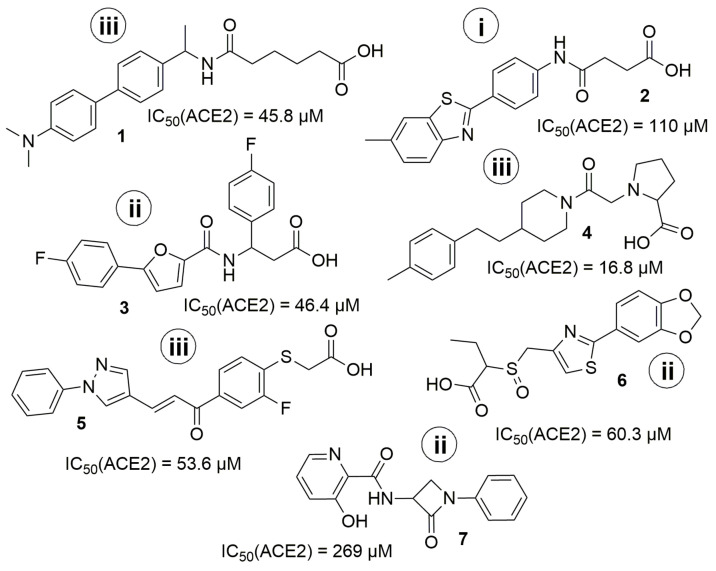
The structure of identified hits. (**i**) from “ultrafast’’ docking; (**ii**) from docking; (**iii**) from pharmacophore modeling.

The more detailed analysis of the hits showed that despite the almost identical distribution of compounds in an in vitro study from three selections (ultrafast docking/”classical’’ docking/pharmacophore modelling) the number of hits was different. There was only one hit coming from the “ultrafast’’ set and three hits from each of the others. The most promising hit (Z1459912954) came from “pharmacophore modelling’’ selection. However, the results obtained do not allow us to make strict conclusions about whether any of the three models is better, because the number of compounds validated in vitro from each set was relatively small. Moreover, the initial training set of known ACE2 inhibitors used was also critically small. The results obtained (including negative ones) will be used in the future for the correction of the computational models. The seven hit-compounds obtained represent two new chemotypes and could be used as a basis for a further hit to lead optimization (Figure 10.). The most potent hits **4, 1,** and **3** have no known neighbors (at default 95% similarity) in the current ChEMBL release 30. It is also notable, that the BBB-penetration capability prediction using boiled-egg diagram [37] as ADME parameters representation, showed that the compounds **4**, **1,** and reference compound **MLN-4760** have the potential for crossing BBB (see Supplementary Materials, Figure S8 for details).

## 4. Conclusions

Using a combination of workflows from three in silico partner groups, this article describes an experimentally validated prospective virtual screening campaign for ACE2-binders, also considering their selectivity with respect to off-targets (ACE and NEP)—albeit the latter aspect was not yet externally validated. The most diverse hits were selected and ranked according to the possible off-target activity.

Assay conditions were adjusted after three steps of validation: appropriate volume and dilutions of enzyme and substrate were chosen, in addition, correlation with reference compounds activities was achieved.

The combined set of the most promising molecules, taken from the QSAR modeling, ultrafast and classical docking hitlists (577 compounds overall, Enamine screening collection used as an input source), were tested in HTS mode in an enzymatic assay using fluorescence method. Seven compounds were selected for dose-response assay. Although none of the seven shows activity in the nanomolar region, and only two compounds match the optimal requirements for BBB-penetration, their strong point is novelty: these ligands present two novel ACE2-binding chemotypes and could be used for further structural optimization.

Despite the very limited scale of the in vitro screening campaign, all three modeling approaches discussed in this work and in our previous paper [12] contributed some hit molecules to the in vitro stage.

## Figures and Tables

**Table 1:** The analytical signal quality of assay vs. dilution of enzyme and substrate.

Substrate	1:1		1:2		1:4		1:8	
Enzyme	S/B	Z-Prime	S/B	Z-Prime	S/B	Z-Prime	S/B	Z-Prime
1:1	24.29	0.92	13.14	0.87	7.26	0.80	4.44	0.82
1:2	13.61	0.90	7.68	0.94	4.46	0.90	2.97	0.79
1:4	6.77	0.94	4.09	0.87	2.62	0.78	1.92	0.82
1:8	4.56	0.89	2.87	0.80	1.96	0.73	1.56	0.60

## Data Availability

The data presented in this study are available on request from the corresponding authors.

## Supplementary Materials

The following supporting information can be downloaded at: https://www.mdpi.com/article/10.3390/molecules27175400/s1, Figure S1: The kinetic curves of positive (inhibitor) control (1–40 µL; 2–25 µL) and negative (enzyme) control (3–40 µL; 4–25 µL); Figure S2: Dependence of relative rate of the reaction (RFU/t) from time of the reaction for positive and negative control for 40 µL and 25 µL; Figure S3: The kinetic curves of positive control and negative control for the undiluted substrate and diluted enzyme (1:1; 1:2; 1:4 and 1:8); Figure S4: The kinetic curves of positive control and negative control for the 1:2 diluted substrate and diluted enzyme (1:1; 1:2; 1:4 and 1:8); Figure S5: The kinetic curves of positive control and negative control for the 1:4 diluted substrate and diluted enzyme (1:1; 1:2; 1:4 and 1:8); Figure S6: The kinetic curves of positive control and negative control for the 1:8 diluted substrate and diluted enzyme (1:1; 1:2; 1:4 and 1:8); Figure S7: DRC for two reference compounds (MLN-4760 and Abcam ref) in optimized conditions; Figure S8: A boiled-egg diagram for the hit compounds (**1**–**7**) and reference molecule MLN-4760 (DiCl); Table S1: Molecules with literature Ki measurements values against ACE2 taken from CHEMBL database; Table S2: Molecules with literature Ki measurements values against ACE taken from CHEMBL database; Table S3: Molecules with literature Ki measurements values against NEP taken from CHEMBL database; Table S4: Virtual hits obtained using UNISTRA pharmacophore search, 202 molecules in total; Table S5: Virtual hits obtained using ENAMINE docking; Table S6: Virtual hits obtained using Ultrafast docking method; Table S7: The values of standard deviations and coefficient of variations for relative reaction rate (RFU/t) at different volumes and reaction times; Table S8: The QC parameters values of two plates used in screening; Table S9: Hits selected according to hit criteria Inh% >3 × SD + Avg_plate. References [12,37,38,39] are cited in the supplementary materials.
