# Supplementary material for: In Vitro Evaluation of In Silico Screening Approaches in Search for Selective ACE2 Binding Chemical Probes
Source: Molecules. 2022 Aug 24;27(17):5400. doi: 10.3390/molecules27175400 (PMC9458095; doi:10.3390/molecules27175400)
Supplement: Supplementary file 1 [file molecules-27-05400-s001.zip › molecules-1860149-supplementary.pdf]

IN VITRO **EVALUATION OF** *IN SILICO* **SCREENING APPROACHES IN SEARCH FOR SELECTIVE ACE2 BINDING CHEMICAL PROBES**

TABLE OF CONTENT – SUPPLEMENTARY INFORMATION

1. Training set for ACE2 target (CHEMBL)……………………………………………………..………………1

2. Training set for ACE anti target (CHEMBL)………………………………………………………………………3

3. Training set for NEP anti target (CHEMBL)…………………………………………………………...……7

4. Selection of compounds for *in vitro* tests after virtual screening ……………………………..…………..21

4.1 *UNISTRA virtual hits*………………………………………………….……………………….….……21

4.2 ENAMINE virtual hits……………………………………………………………………….……..……27

4.3 *Ultrafast docking virtual hits*[1]…………………………… ………..……………………..……33

5. ACE2 assay calibration and *in vitro* studies of selected virtual hits…………………………..……….…40

5.1 Optimization of volume……………………………………………………………...…..………40

5.2 Optimization of enzyme and substrate dilution……………………………………………….………41

5.3 IC50 calculations for two reference inhibitors………………………………..……………..……43

5.4 Primary screen QC and results…………………………………………………...…….…...……44

6. Evaluation of the BBB penetration ability of the hit molecules...………………………….…………….……45

7. References……………………………………………………………………………………………..…….……..45

1. Training set for ACE2 target (CHEMBL)

Table S1. Molecules with literature Ki measurements values against ACE2 taken from CHEMBL database.

Smiles	CHEMBL ID
OC(=O)[C@H](Cc1ccccc1)CP(O)(=O)[C@H]1CCCN1	CHEMBL261033
C[C@@H](N)P(O)(=O)C[C@@H](Cc1ccccc1)C(O)=O	CHEMBL405913
CC(C)C[C@@H](NC(=O)OCc1ccccc1)P(O)(=O)C[C@@H](Cc1ccccc1)C(O)=O	CHEMBL264665
C[C@H](CP(O)(=O)[C@H]1CCCN1C(=O)OCc1ccccc1)C(O)=O	CHEMBL409713
CC(C)(S)C(=O)N[C@@H](Cc1ccc(cc1)-c1ccccc1)C(O)=O	CHEMBL404044
OC(=O)[C@H](Cc1ccc(cc1)-c1ccccc1)NC(=O)[C@H](S)Cc1ccccc1	CHEMBL163454
CC(C)C[C@H](NC(C)=O)C(=O)N[C@H](Cc1ccccc1)P(O)(=O)C[C@@H](Cc1ccccc1)C(O)=O	CHEMBL408705
OC(=O)[C@H](Cc1ccc(cc1)-c1ccccc1)NC(=O)[C@@H](S)CCc1ccccc1	CHEMBL402987
CC(C)C[C@@H](NC(=O)[C@H](Cc1c[nH]cn1)NC(C)=O)P(O)(=O)C[C@@H](Cc1ccccc1)C(O)=O	CHEMBL408448
OC(=O)[C@H](Cc1ccc(cc1)-c1ccccc1)NC(=O)[C@@H](S)Cc1ccc2ccccc2c1	CHEMBL272925
OC(=O)[C@H](Cc1ccc(cc1)-c1ccccc1)NC(=O)[C@@H](S)CC1CCCCC1	CHEMBL271224
OC(=O)[C@H](Cc1ccc(cc1)-c1ccccc1)NC(=O)CS	CHEMBL258333
OC(=O)[C@H](Cc1ccccc1)CP(O)(=O)[C@H]1CCCN1C(=O)OCc1ccccc1	CHEMBL260677

CC(C)C[C@H](CP(O)(=O)[C@H]1CCCN1C(=O)OCc1ccccc1)C(O)=O	CHEMBL411298
CC(C)C[C@@H](NC(=O)[C@H](Cc1c[nH]cn1)NC(C)=O)P(O)(=O)CC(Cc1cc(no1)-c1ccccc1)C(O)=O	CHEMBL408182
OC(=O)[C@H](Cc1ccc(cc1)-c1ccccc1)NC(=O)[C@@H](S)Cc1ccccc1	CHEMBL350414
CC[C@@H](C)[C@H](S)C(=O)N[C@@H](Cc1ccc(OCc2cc(cc(c2)C(F)(F)F)C(F)(F)F)cc1)C(O)=O	CHEMBL252009
OC(=O)[C@H](Cc1ccc(cc1)-c1ccccc1)NC(=O)[C@@H](S)c1ccccc1	CHEMBL437595
OC(=O)[C@H](Cc1ccc(cc1)-c1ccccc1)NC(=O)[C@@H](S)C1CCCCC1	CHEMBL257026
CC[C@@H](C)[C@H](S)C(=O)N[C@@H](Cc1ccccc1-c1ccccc1)C(O)=O	CHEMBL398545
CC[C@@H](C)[C@H](S)C(=O)N[C@@H](Cc1ccc(OCc2ccc(cc2)C(F)(F)F)cc1)C(O)=O	CHEMBL251809
C[C@H](NC(C)=O)C(=O)N1CCC[C@@H]1P(O)(=O)C[C@@H](Cc1ccccc1)C(O)=O	CHEMBL409721
CC(C)(C)C[C@H](S)C(=O)N[C@@H](Cc1ccc(cc1)-c1ccccc1)C(O)=O	CHEMBL271223
CC(=O)N[C@@H](CCC(O)=O)C(=O)N1CCC[C@@H]1P(O)(=O)C[C@@H](Cc1ccccc1)C(O)=O	CHEMBL263663
C[C@H](S)C(=O)N[C@@H](Cc1ccc(cc1)-c1ccccc1)C(O)=O	CHEMBL257727
CC(C)[C@H](NC(C)=O)C(=O)N1CCC[C@@H]1P(O)(=O)C[C@@H](Cc1ccccc1)C(O)=O	CHEMBL411052
CC(=O)N[C@@H](CCCCN)C(=O)N1CCC[C@@H]1P(O)(=O)C[C@@H](Cc1ccccc1)C(O)=O	CHEMBL411942
CC[C@@H](C)[C@H](S)C(=O)N[C@@H](Cc1ccccc1OCc1ccccc1)C(O)=O	CHEMBL254493
CC[C@@H](C)[C@H](S)C(=O)N[C@@H](Cc1cccc(Oc2ccccc2)c1)C(O)=O	CHEMBL252003
CC(=O)N[C@@H](Cc1ccc(O)cc1)C(=O)N1CCC[C@@H]1P(O)(=O)C[C@@H](Cc1ccccc1)C(O)=O	CHEMBL410509
CC(=O)N[C@@H](Cc1ccccc1)C(=O)N1CCC[C@@H]1P(O)(=O)C[C@@H](Cc1ccccc1)C(O)=O	CHEMBL258698
CC[C@@H](C)[C@H](S)C(=O)N[C@@H](Cc1ccc(OCc2ccccc2)cc1)C(O)=O	CHEMBL254703
CC[C@@H](C)[C@H](S)C(=O)N[C@@H](Cc1ccc(OCc2ccc(F)cc2)cc1)C(O)=O	CHEMBL254911
CC[C@@H](C)[C@H](S)C(=O)N[C@@H](Cc1cccc(OCc2ccccc2)c1)C(O)=O	CHEMBL254495
OC(=O)[C@H](Cc1ccc(cc1)-c1ccccc1)NC(=O)[C@@H](S)C1CCC1	CHEMBL257229
CC[C@@H](C)[C@H](S)C(=O)N[C@@H](Cc1ccc2ccccc2c1)C(O)=O	CHEMBL253428
CC[C@@H](C)[C@H](S)C(=O)N[C@@H](Cc1ccc(Oc2ccccc2)cc1)C(O)=O	CHEMBL254282
CC[C@@H](C)[C@H](S)C(=O)N[C@@H](Cc1cccc(c1)-c1ccccc1)C(O)=O	CHEMBL401397
CC[C@@H](C)[C@H](S)C(=O)N[C@@H](Cc1cccc2ccccc12)C(O)=O	CHEMBL400527
CC(=O)N[C@@H](Cc1c[nH]cn1)C(=O)N1CCC[C@@H]1P(O)(=O)C[C@@H](Cc1ccccc1)C(O)=O	CHEMBL261423
CC[C@@H](C)[C@H](S)C(=O)N[C@@H](Cc1ccc(OCc2ccc(F)cc2F)cc1)C(O)=O	CHEMBL398771
OC(=O)[C@H](Cc1ccc(cc1)-c1ccccc1)NC(=O)[C@@H](S)C1CCCC1	CHEMBL404117
CCCC[C@H](S)C(=O)N[C@@H](Cc1ccc(cc1)-c1ccccc1)C(O)=O	CHEMBL257270
CC[C@H](C)[C@H](S)C(=O)N[C@@H](Cc1ccc(cc1)-c1ccccc1)C(O)=O	CHEMBL269996
CC[C@@H](C)[C@H](S)C(=O)N[C@@H](Cc1ccc(cc1)-c1ccccc1)C(O)=O	CHEMBL252391
CC(C)[C@H](S)C(=O)N[C@@H](Cc1ccc(cc1)-c1ccccc1)C(O)=O	CHEMBL269997
CC[C@@H](C)[C@H](S)C(=O)N[C@@H](Cc1ccccc1)C(O)=O	CHEMBL400526
CC(C)C[C@H](S)C(=O)N[C@@H](Cc1ccc(cc1)-c1ccccc1)C(O)=O	CHEMBL271225
CC[C@H](S)C(=O)N[C@@H](Cc1ccc(cc1)-c1ccccc1)C(O)=O	CHEMBL257726
CC[C@@H](C)[C@H](S)C(=O)N[C@@H](Cc1ccc(O)cc1)C(O)=O	CHEMBL253224

CC(C)C[C@H](NC(C)=O)C(=O)N1CCC[C@@H]1P(O)(=O)CC(Cc1cc(no1)-c1ccccc1)C(O)=O	CHEMBL261121
CC[C@@H](C)[C@H](S)C(=O)N[C@@H](Cc1ccccc1Oc1ccccc1)C(O)=O	CHEMBL251804
CC[C@@H](C)[C@H](S)C(=O)N[C@@H](Cc1ccc(OCc2ccc(F)c(F)c2)cc1)C(O)=O	CHEMBL251808
CC(=O)N[C@@H](Cc1c[nH]cn1)C(=O)N1CCC[C@@H]1P(O)(=O)CC(Cc1cc(no1)-c1ccccc1)C(O)=O	CHEMBL409105
CC(C)C[C@H](NC(C)=O)C(=O)N1CCC[C@@H]1P(O)(=O)C[C@@H](Cc1ccccc1)C(O)=O	CHEMBL260273

2. Training set for ACE anti target (CHEMBL)

Table S2. Molecules with literature Ki measurements values against ACE taken from CHEMBL database.

11	CHEMBL ID
CC[C@H](C)[C@H](NC(C)=O)C(=O)N[C@@H](CC(C)C)C(=O)N[C@@H](Cc1ccc(O)cc1)P(O)(O)=O	CHEMBL3264009
CN[C@@H](C(C)C)C(=O)N[C@@H](Cc1ccc(O)cc1)C(=O)N[C@@H](Cc1ccc(O)cc1)P(O)(O)=O	CHEMBL3264008
CC(C)(S)C(=O)N[C@@H](Cc1ccc(cc1)-c1ccccc1)C(O)=O	CHEMBL404044
OC(=O)[C@@H]1CCC[C@@H]2SCC[C@H](NC(=O)[C@@H](S)Cc3ccccc3)C(=O)N12	CHEMBL289556
OC(=O)[C@H](Cc1ccccc1)NC(=O)[C@@H](Cc1cc(no1)-c1ccccc1)CP(O)(=O)[C@H](Cc1ccccc1)NC(=O)OCc1ccccc1	CHEMBL567628
OC(=O)[C@H](Cc1ccccc1)NC(=O)[C@@H](Cc1cc(no1)-c1ccccc1)CP(O)(=O)[C@H](Cc1ccccc1)NC(=O)OCc1ccccc1	CHEMBL567628
COc1ccc2[C@@H](CCc2c1)[C@@H](CS)C(=O)N[C@@H](Cc1c[nH]c2ccccc12)C(O)=O	CHEMBL305388
Oc1ccc2c(oc3c(O)c(O)ccc3c2=O)c1O	CHEMBL477921
C[C@H](NC(=O)[C@@H](CSC(O)=O)Cc1ccc2OCOc2c1)C(=O)OCc1ccccc1	CHEMBL325055
C[C@@H](SC(C)=O)C(=O)N1[C@@H](C[C@@H]([C@@H]1c1ccccc1)C(O)=O)C(O)=O	CHEMBL1788109
OC(=O)C(CCc1ccccc1)NC1Cc2ccccc2[C@H]2CCC[C@H](N2C1=O)C(O)=O	CHEMBL358203
OC(=O)[C@H](Cc1c[nH]c2ccccc12)NC(=O)[C@@H](Cc1cc(no1)-c1ccccc1)CP(O)(=O)[C@H](Cc1ccccc1)NC(=O)OCc1ccccc1	CHEMBL577754
OC(=O)[C@H](Cc1ccc(O)cc1)NC(=O)[C@@H](Cc1cc(no1)-c1ccccc1)CP(O)(=O)[C@H](Cc1ccccc1)NC(=O)OCc1ccccc1	CHEMBL570953
OC(=O)[C@H](Cc1c[nH]c2ccccc12)NC(=O)C(Cc1ccc(cc1)-c1ccccc1)CP(O)(=O)[C@H](Cc1ccccc1)NC(=O)OCc1ccccc1	CHEMBL570740
OC(=O)[C@H](Cc1ccc(O)cc1)NC(=O)[C@@H](Cc1cc(no1)-c1ccccc1)CP(O)(=O)[C@H](Cc1ccccc1)NC(=O)OCc1ccccc1	CHEMBL570953
OC(=O)[C@H](Cc1ccccc1)NC(=O)[C@@H](Cc1cc(no1)-c1ccccc1)CP(O)(=O)[C@H](Cc1ccccc1)NC(=O)OCc1ccccc1	CHEMBL567628
OC(=O)[C@H](Cc1c[nH]c2ccccc12)NC(=O)[C@@H](Cc1cc(no1)-c1ccccc1)CP(O)(=O)[C@H](Cc1ccccc1)NC(=O)OCc1ccccc1	CHEMBL577754
CC[C@H](C)[C@H](NC(C)=O)C(=O)N[C@@H](Cc1ccccc1)C(=O)N[C@@H](Cc1ccc(O)cc1)P(O)(O)=O	CHEMBL3264010

OC(=O)[C@H](Cc1c[nH]c2ccccc12)NC(=O)[C@@H](Cc1cc(no1)-c1ccccc1)CP(O)(=O)[C@H](Cc1ccccc1)NC(=O)OCc1ccccc1	CHEMBL577754
OC(=O)[C@@H]1CCCN1C(=O)CCC(=O)[C@H](Cc1ccccc1)NC(=O)c1ccccc1	CHEMBL435360
OC(=O)[C@H](Cc1ccccc1)NC(=O)CCC(=O)[C@H](Cc1ccccc1)NC(=O)c1ccccc1	CHEMBL213105
CC[C@H](C)[C@H](S)C(=O)N[C@@H](Cc1ccc(cc1)-c1ccccc1)C(O)=O	CHEMBL269996
OC(=O)[C@H](Cc1ccc(cc1)-c1ccccc1)NC(=O)[C@H](S)Cc1ccccc1	CHEMBL163454
CC[C@H](S)C(=O)N[C@@H](Cc1ccc(cc1)-c1ccccc1)C(O)=O	CHEMBL257726
CC[C@@H](C)[C@H](S)C(=O)N[C@@H](Cc1ccc2ccccc2c1)C(O)=O	CHEMBL253428
CC(C)(C)OC(=O)N[C@@H](Cc1ccccc1)C(=O)CCC(=O)N[C@@H](Cc1ccccc1)C(O)=O	CHEMBL212864
CCCC[C@H](S)C(=O)N[C@@H](Cc1ccc(cc1)-c1ccccc1)C(O)=O	CHEMBL257270
C[C@H](S)C(=O)N[C@@H](Cc1ccc(cc1)-c1ccccc1)C(O)=O	CHEMBL257727
OC(=O)[C@H](Cc1c[nH]c2ccccc12)NC(=O)[C@@H](Cc1cc(no1)-c1ccccc1)CP(O)(=O)[C@H](Cc1ccccc1)NC(=O)OCc1ccccc1	CHEMBL577754
OC(=O)[C@H](Cc1c[nH]c2ccccc12)NC(=O)C(CC1CC(=NO1)c1ccc(cc1)-c1ccccc1)CP(O)(=O)[C@H](Cc1ccccc1)NC(=O)OCc1ccccc1	CHEMBL570722
OC(=O)[C@H](Cc1ccc(O)cc1)NC(=O)[C@@H](Cc1cc(no1)-c1ccccc1)CP(O)(=O)[C@H](Cc1ccccc1)NC(=O)OCc1ccccc1	CHEMBL570953
OC(=O)[C@H](Cc1ccc(O)cc1)NC(=O)[C@@H](Cc1cc(no1)-c1ccccc1)CP(O)(=O)[C@H](Cc1ccccc1)NC(=O)OCc1ccccc1	CHEMBL570953
Oc1cc(O)c2c(c1)oc1cc(O)c(O)cc1c2=O	CHEMBL187265
OC(=O)[C@H](Cc1c[nH]c2ccccc12)NC(=O)[C@@H](Cc1cc(no1)-c1ccccc1)CP(O)(=O)[C@H](Cc1ccccc1)NC(=O)OCc1ccccc1	CHEMBL577754
OC(=O)[C@H](Cc1ccc(cc1)-c1ccccc1)NC(=O)[C@@H](S)Cc1ccc2ccccc2c1	CHEMBL272925
OC(=O)[C@H](Cc1ccc(cc1)-c1ccccc1)NC(=O)[C@@H](S)C1CCCC1	CHEMBL404117
CC[C@@H](C)[C@H](S)C(=O)N[C@@H](Cc1ccc(cc1)-c1ccccc1)C(O)=O	CHEMBL252391
CC[C@@H](C)[C@H](S)C(=O)N[C@@H](Cc1cccc(Oc2ccccc2)c1)C(O)=O	CHEMBL252003
CC[C@@H](C)[C@H](S)C(=O)N[C@@H](Cc1ccccc1Oc1ccccc1)C(O)=O	CHEMBL251804
CC[C@@H](C)[C@H](S)C(=O)N[C@@H](C)C(O)=O	CHEMBL254900
CC(C)C[C@@H](N1CCO[C@H]([C@H](NC(=O)[C@H](S)Cc2ccccc2)C1=O)c1ccccc1)C(O)=O	CHEMBL311838
CO[C@@H]1[C@@H](OC(N)=O)[C@@H](O)[C@H](Oc2ccc3c(O)c(NC(=O)c4ccc(O)c(CC=C(C)C)c4)c(=O)oc3c2C)OC1(C)C	CHEMBL36506
OC(=O)[C@H](Cc1c[nH]c2ccccc12)NC(=O)[C@@H]1CCC[C@H]1P(O)(=O)[C@H](Cc1ccccc1)NC(=O)OCc1ccccc1	CHEMBL571716
CC(C)(C)OC(=O)N[C@@H](Cc1ccccc1)C(=O)CCC(=O)N[C@@H](Cc1c[nH]c2ccccc12)C(O)=O	CHEMBL215163
C[C@H](CP(O)(=O)[C@H](Cc1ccccc1)NC(=O)[C@H](CC(O)=O)NC(C)=O)C(=O)N[C@@H](C)C(N)=O	CHEMBL1235767
OC(=O)[C@H](Cc1c[nH]c2ccccc12)NC(=O)[C@@H]1CCCC1P(O)(=O)[C@H](Cc1ccccc1)NC(=O)OCc1ccccc1	CHEMBL2153740
C[C@@H](NP(O)(O)=O)C(=O)N1CCC[C@H]1C(O)=O	CHEMBL1161022

NCCCC[C@H](N[C@H](CCc1ccccc1)C(O)=O)C(=O)N[C@@H](Cc1c[nH]c2ccccc12)C(O)=O	CHEMBL212711
OC(=O)[C@H](Cc1c[nH]c2ccccc12)NC(=O)CCC(=O)[C@H](Cc1ccccc1)NC(=O)c1ccccc1	CHEMBL385901
CC[C@@H](C)[C@H](S)C(=O)N[C@@H](Cc1ccc(cc1)-c1ccccc1)C(O)=O	CHEMBL252391
NCCCC[C@H](N[C@@H](CCc1ccccc1)C(O)=O)C(=O)N1CCC[C@H]1C(O)=O	CHEMBL1237
NC(=O)[C@H](CCC(O)=O)NC(=O)[C@H](CCC(O)=O)NC(=O)[C@H](Cc1cc(no1)-c1ccc(cc1)-c1cccc(Cl)c1)CP(O)(=O)c1ccc(Br)cc1	CHEMBL507420
OC(=O)[C@H](Cc1c[nH]c2ccccc12)NC(=O)[C@@H]1CCC[C@H]1P(O)(=O)[C@H](Cc1ccccc1)NC(=O)OCc1ccccc1	CHEMBL571716
OC(=O)[C@H](Cc1ccc(O)cc1)NC(=O)[C@H](Cc1cc(no1)-c1ccccc1)CP(O)(=O)[C@H](Cc1ccccc1)NC(=O)OCc1ccccc1	CHEMBL570732
OC(=O)[C@H](Cc1ccccc1)NC(=O)[C@H](Cc1cc(no1)-c1ccccc1)CP(O)(=O)[C@H](Cc1ccccc1)NC(=O)OCc1ccccc1	CHEMBL570718
OC(=O)[C@H](Cc1c[nH]c2ccccc12)NC(=O)C(CC1CC(=NO1)c1ccc(cc1)C(F)(F)F)CP(O)(=O)[C@H](Cc1ccccc1)NC(=O)OCc1ccccc1	CHEMBL570704
C[C@H](N)C(=O)N1CCC[C@H]1C(O)=O	CHEMBL414828
CC(C)(C)C[C@H](S)C(=O)N[C@@H](Cc1ccc(cc1)-c1ccccc1)C(O)=O	CHEMBL271223
CC(C)C[C@H](S)C(=O)N[C@@H](Cc1ccc(cc1)-c1ccccc1)C(O)=O	CHEMBL271225
CC[C@H](C)[C@H](NC(=O)[C@H](CCC(N)=O)NC(=O)[C@@H]1CCCN1C(=O)[C@H](CCCN=C(N)N)NC(=O)[C@@H]1CCCN1C(=O)[C@H](Cc1c[nH]c2ccccc12)NC(=O)[C@@H](N)CCC(O)=O)C(=O)N1CCC[C@H]1C(=O)N1CCC[C@H]1C(O)=O	CHEMBL216386
C[C@H](NC(=O)C(CC1CC(=NO1)c1ccccc1)CP(O)(=O)[C@H](Cc1ccccc1)NC(=O)OCc1ccccc1)C(O)=O	CHEMBL570811
OC(=O)C(CCc1ccccc1)NC(Cc1ccccc1)C=O	CHEMBL152307
OC(=O)[C@H](Cc1ccccc1)NC(=O)[C@@H](Cc1cc(no1)-c1ccccc1)CP(O)(=O)[C@H](Cc1ccccc1)NC(=O)OCc1ccccc1	CHEMBL567628
Oc1ccc2c(oc3cc(O)c(O)cc3c2=O)c1O	CHEMBL477740
CC[C@H](C)[C@H](NC(C)=O)C(=O)N[C@@H](Cc1ccc(O)cc1)C(=O)N[C@@H](Cc1ccc(O)cc1)P(O)(O)=O	CHEMBL1233799
CC[C@@H](C)[C@H](S)C(=O)N[C@@H](Cc1ccc(OCc2cc(cc(c2)C(F)(F)F)C(F)(F)F)cc1)C(O)=O	CHEMBL252009
CC[C@@H](C)[C@H](S)C(=O)N[C@@H](Cc1ccc(OCc2ccc(F)c(F)c2)cc1)C(O)=O	CHEMBL251808
CC[C@@H](C)[C@H](S)C(=O)N[C@@H](Cc1ccccc1-c1ccccc1)C(O)=O	CHEMBL398545
CC[C@@H](C)[C@H](S)C(=O)N[C@@H](Cc1cccc2ccccc12)C(O)=O	CHEMBL400527
NCCCC[C@H](N[C@@H](CCc1ccccc1)C(O)=O)C(=O)N[C@@H](Cc1c[nH]c2ccccc12)C(O)=O	CHEMBL212710
OC(=O)[C@@H]1CCC[C@@H]2SCC[C@H](NC(=O)[C@@H](S)Cc3ccccc3)C(=O)N12	CHEMBL289556
Oc1cc(O)c2c(c1)oc1c(O)c(O)ccc1c2=O	CHEMBL448040
OC(=O)[C@H](Cc1ccc(cc1)-c1ccccc1)NC(=O)[C@@H](S)c1ccccc1	CHEMBL437595
CC[C@H](C)[C@H](NC(C)=O)C(=O)N[C@@H](Cc1ccc(O)cc1)C(=O)N[C@@H](Cc1ccc(O)cc1)P(O)(O)=O	CHEMBL1233799
CS(=O)(=O)N[C@@H](CCCCN)C(=O)NC[C@H](CC1(CCCC1)C(=O)N[C@@H](Cc1ccc(O)cc1)C(O)=O)C(O)=O	CHEMBL42583

OC(=O)[C@H](Cc1ccc(O)cc1)NC(=O)[C@@H](Cc1cc(no1)-c1ccccc1)CP(O)(=O)[C@H](Cc1ccccc1)NC(=O)OCc1ccccc1	CHEMBL570953
OC(=O)CN1Cc2ccccc2CC(N[C@H](CCc2ccccc2)C(O)=O)C1=O	CHEMBL107695
C[C@H](CS)C(=O)N1CCC[C@H]1C(O)=O	CHEMBL1560
CC(C)(C)OC(=O)N[C@@H](Cc1ccccc1)C(=O)CCC(=O)N1CCC[C@H]1C(O)=O	CHEMBL386032
CC[C@@H](C)[C@H](S)C(=O)N[C@@H](Cc1ccc(Oc2ccccc2)cc1)C(O)=O	CHEMBL254282
OC(=O)[C@H](Cc1ccc(cc1)-c1ccccc1)NC(=O)[C@@H](S)CCc1ccccc1	CHEMBL402987
CC[C@@H](C)[C@H](S)C(=O)N[C@@H](Cc1ccccc1OCc1ccccc1)C(O)=O	CHEMBL254493
OC(=O)[C@H](Cc1ccc(cc1)-c1ccccc1)NC(=O)CS	CHEMBL258333
CC(=O)N[C@@H](CC(O)=O)C(=O)NC[C@H](CC1(CCCC1)C(=O)N[C@@H](Cc1ccc(O)cc1)C(O)=O)C(O)=O	CHEMBL4462603
CC[C@@H](C)[C@H](S)C(=O)N[C@@H](Cc1ccc(OCc2ccc(F)cc2)cc1)C(O)=O	CHEMBL254911
C[C@H](NCS)C(=O)N1CCC[C@H]1C(O)=O	CHEMBL166164
NCCCC[C@H](N[C@@H](CCc1ccccc1)C(O)=O)C(=O)N1CCC[C@H]1C(O)=O	CHEMBL1237
CC[C@@H](C)[C@H](S)C(=O)N[C@@H](Cc1ccc(OCc2ccc(cc2)C(F)(F)F)cc1)C(O)=O	CHEMBL251809
CC[C@@H](C)[C@H](S)C(=O)N[C@@H](Cc1ccc(OCc2ccccc2)cc1)C(O)=O	CHEMBL254703
CC[C@@H](C)[C@H](S)C(=O)N[C@@H](Cc1cccc(c1)-c1ccccc1)C(O)=O	CHEMBL401397
CC[C@@H](C)[C@H](S)C(=O)N[C@@H](Cc1ccc(O)cc1)C(O)=O	CHEMBL253224
CC[C@@H](C)[C@H](S)C(=O)N[C@@H](Cc1ccccc1)C(O)=O	CHEMBL400526
OC(=O)[C@@H]1CCCN1C(=O)CCC(=O)C(Cc1ccccc1)NC(=O)c1ccccc1	CHEMBL416147
CC(=O)N[C@@H](CC(O)=O)C(=O)NC[C@H](CC1(CCCC1)C(=O)N[C@@H](Cc1ccc(O)cc1)C(O)=O)C(O)=O	CHEMBL4462603
CN[C@@H](C(C)C)C(=O)N[C@@H](Cc1ccccc1)C(=O)N[C@@H](Cc1ccc(O)cc1)P(O)(O)=O	CHEMBL3264007
C[C@H](CP(O)(=O)[C@H](Cc1ccccc1)NC(=O)[C@H](CC(O)=O)NC(C)=O)C(=O)N[C@@H](C)C(N)=O	CHEMBL1235767
OC(=O)[C@H](Cc1ccc(O)cc1)NC(=O)[C@@H](Cc1cc(no1)-c1ccccc1)CP(O)(=O)[C@H](Cc1ccccc1)NC(=O)OCc1ccccc1	CHEMBL570953
CC[C@@H](C)[C@H](S)C(=O)N[C@@H](Cc1ccc(OCc2ccc(F)cc2F)cc1)C(O)=O	CHEMBL398771
CC[C@@H](C)[C@H](S)C(=O)N[C@@H](Cc1cccc(OCc2ccccc2)c1)C(O)=O	CHEMBL254495
CC[C@@H](C)[C@H](S)C(=O)NCC(O)=O	CHEMBL401086
CCCc1nc2c(C)cc(cc2n1Cc1ccc(cc1)-c1ccccc1C(O)=O)-c1nc2ccccc2n1C	CHEMBL1017
CC[C@H](C)[C@H](NC(C)=O)C(=O)N[C@@H](Cc1ccccc1)C(=O)N[C@@H](Cc1ccc(O)cc1)P(O)(O)=O	CHEMBL3264010
CC[C@H](C)[C@H](NC(C)=O)C(=O)N[C@@H](CCC(O)=O)C(=O)N[C@@H](Cc1ccc(O)cc1)P(O)(O)=O	CHEMBL3264356
CC[C@H](C)[C@H](NC(C)=O)C(=O)N[C@@H](CCC(O)=O)C(=O)N[C@@H](Cc1ccc(O)cc1)P(O)(O)=O	CHEMBL3264356
OC(=O)[C@@H]1CCCN1	CHEMBL54922
OC(=O)[C@H](Cc1ccc(cc1)-c1ccccc1)NC(=O)[C@@H](S)C1CCC1	CHEMBL257229
CC(C)[C@H](S)C(=O)N[C@@H](Cc1ccc(cc1)-c1ccccc1)C(O)=O	CHEMBL269997
OC(=O)[C@H](Cc1ccc(cc1)-c1ccccc1)NC(=O)[C@@H](S)CC1CCCCC1	CHEMBL271224
OC(=O)[C@H](Cc1ccc(cc1)-c1ccccc1)NC(=O)[C@@H](S)C1CCCCC1	CHEMBL257026

OC(=O)[C@@H]1C[C@H](CN1C(=O)CP(O)(=O)CCCCc1ccccc1)C1CCCCC1	CHEMBL581
NCCCC[C@H](N[C@@H](CCc1ccccc1)C(O)=O)C(=O)N1CCC[C@H]1C(O)=O	CHEMBL1237
CN[C@@H](C(C)C)C(=O)N[C@@H](Cc1ccc(O)cc1)C(=O)N[C@@H](Cc1ccc(O)cc1)P(O)(O)=O	CHEMBL3264008
C[C@H](NP(O)(=O)CCc1ccccc1)C(=O)N1CCC[C@H]1C(O)=O	CHEMBL309601
CS(=O)(=O)N[C@@H](CCCCN)C(=O)NC[C@H](CC1(CCCC1)C(=O)N[C@@H](Cc1ccc(O)cc1)C(O)=O)C(O)=O	CHEMBL42583
OC(=O)[C@H](Cc1ccc(cc1)-c1ccccc1)NC(=O)[C@@H](S)Cc1ccccc1	CHEMBL350414
CC[C@H](C)[C@H](NC(C)=O)C(=O)N[C@@H](CC(C)C)C(=O)N[C@@H](Cc1ccc(O)cc1)P(O)(O)=O	CHEMBL3264009
CN[C@@H](C(C)C)C(=O)N[C@@H](Cc1ccccc1)C(=O)N[C@@H](Cc1ccc(O)cc1)P(O)(O)=O	CHEMBL3264007
Cc1nc(Nc2ncc(s2)C(=O)Nc2c(C)cccc2Cl)cc(n1)N1CCN(CCO)CC1	CHEMBL1421
OC(=O)[C@H](Cc1ccc(O)cc1)NC(=O)[C@@H](Cc1cc(no1)-c1ccccc1)CP(O)(=O)[C@H](Cc1ccccc1)NC(=O)OCc1ccccc1	CHEMBL570953
OC(=O)[C@@H]1CCC[C@@H]2SCC[C@H](NC(=O)[C@@H](S)Cc3ccccc3)C(=O)N12	CHEMBL289556
OC(=O)[C@H](Cc1c[nH]c2ccccc12)NC(=O)[C@@H]1CCCC1P(O)(=O)[C@H](Cc1ccccc1)NC(=O)OCc1ccccc1	CHEMBL2153740

3. Training set for NEP anti target (CHEMBL)

Table S3. Molecules with literature Ki measurements values against NEP taken from CHEMBL database.

Smiles	CHEMBL ID
CCC(C)C(S)C(=O)NC(Cc1ccc2ccccc2c1)C(O)=O	CHEMBL253428
CC(C)CCNC(=O)C(CC#Cc1ccc(F)cc1F)NCP(O)(O)=O	CHEMBL287971
CC(C)Oc1ccc(CC(NC(=O)C(NCP(O)(O)=O)C(C)C)C(O)=O)cc1	CHEMBL328030
OC(=O)CCNC(=O)C(Cc1ccc(cc1)-c1ccccc1)NCC(O)=O	CHEMBL1829583
CC(C)CC(CC(=O)NC(CO)C(O)=O)C(O)=O	CHEMBL3221963+CHEMBL3221964
OC(=O)C(Cc1ccc(cc1)-c1ccccc1)NC(=O)C(Cc1c[nH]cn1)NCP(O)(O)=O	CHEMBL315669
CCCC(CC1(CCCC1)C(=O)NC1(CO)Cc2ccccc2C1)C(O)=O	CHEMBL212526+CHEMBL211383+CHEMBL377037
CC(S)C(=O)NC1CCc2cccc3CC(N(c23)C1=O)C(O)=O	CHEMBL100044
OC(=O)C1CCN2CCCC(NC(=O)C(S)Cc3ccccc3)C(=O)N12	CHEMBL66254

CC(C)CC(CC(O)=O)C(=O)NC(CO)C(O)=O	CHEMBL3222111+CHEMBL3221965
Cc1cc(ccc1F)S(=O)(=O)NC(C1CCOCC1)C(=O)NO	CHEMBL204593+CHEMBL562246
OC(=O)C(CC#Cc1ccccc1F)NCP(O)(O)=O	CHEMBL448098
CC(N)C(=O)NC(CCCNC(N)=N)C(=O)NC(Cc1ccccc1)C(=O)NC(CO)C(=O)NC(CCCNC(N)=N)C(O)=O	CHEMBL2022226
OC(=O)C1CCCN2N1C(=O)C(CCC2=O)NC(=O)C(S)Cc1ccccc1	CHEMBL66481
NC(CCC(N)=O)C(=O)NC(CCCNC(N)=N)C(=O)NC(Cc1ccccc1Br)C(=O)NC(CO)C(=O)NC(CCCNC(N)=N)C(O)=O	CHEMBL2022232
OC(=O)CCNC(=O)C(Cc1ccccc1)CP(O)(=O)C(Cc1ccccc1)NC(=O)OCc1ccccc1	CHEMBL25341
OC(=O)C(Cc1ccc(cc1)-c1ccccc1)NC(=O)C(S)CCc1ccccc1	CHEMBL402987
CC(C)C(NCP(O)(O)=O)C(=O)NC(Cc1ccc(cc1)C1CCCCC1)C(O)=O	CHEMBL93167
CC(C)CC(NC(=O)C(CS)C(C)c1ccccc1)C(O)=O	CHEMBL273898
OC(=O)C(COc1ccccc1)CC(Cc1ccc(cc1)-c1ccccc1)C(=O)NCc1nn[nH]n1	CHEMBL284818
OC(=O)CNC(=O)C1(CS)CCCC1	CHEMBL3298938
CC(C)OC(=O)N1CC(S)CC1CNCc1cc(F)ccc1F	CHEMBL192441
CC(C(CS)C(=O)NC(Cc1ccc(O)cc1)C(O)=O)c1ccccc1	CHEMBL11616
OC(=O)C(Cc1ccccc1)NC(=O)C(Cc1ccccc1)CP(O)(=O)C(Cc1ccccc1)NC(=O)OCc1ccccc1	CHEMBL27880+CHEMBL282118
COCCC(NC1(CCCC1)C(=O)NC(Cc1nc(co1)-c1ccccc1)C(O)=O)C(O)=O	CHEMBL1778531
NC(CCCNC(N)=N)C(=O)NC(Cc1ccccc1)C(=O)NC(CO)C(=O)NC(CCCNC(N)=N)C(O)=O	CHEMBL2029394
NC(CCC(N)=O)C(=O)NC(CCCNC(N)=N)C(=O)NC(Cc1ccccc1)C(=O)NC(CO)C(O)=O	CHEMBL2029395
CCCN1N(CC(O)=O)C(=O)C(CCC1=O)NC(=O)C(S)Cc1ccccc1	CHEMBL62417
CCCC(NC1(CCCC1)C(=O)NC(Cc1nc(co1)-c1ccccc1)C(O)=O)C(O)=O	CHEMBL1778529
NCCc1c(NC(=O)C(CC#Cc2ccc(F)cc2F)NCP(O)(O)=O)[nH]c2ccccc12	CHEMBL35995
OC(=O)C1CCCCCCc2cccc(CC(CS)C(=O)N1)c2	CHEMBL147526+CHEMBL287358+CHEMBL357584
CC(C)(S)C(=O)NC1CCc2cccc3CC(N(c23)C1=O)C(O)=O	CHEMBL93816

CC1Oc2ccccc2N(CC(O)=O)C(=O)C1NC(=O)C(S)Cc1ccccc1	CHEMBL50559+CHEMBL53879
COCCC(NC1(CCCC1)C(=O)NC(Cc1ncc(o1)-c1ccc(Cl)cc1)C(O)=O)C(O)=O	CHEMBL1778534
OCC(NC(=O)C(CS)Cc1ccccc1)C(O)=O	CHEMBL3298859
OP(O)(=O)CNC(CC#Cc1ccccc1F)C(=O)NCCc1cccc2ccccc12	CHEMBL36809
OC(=O)C1CCCN2CCCC(NC(=O)C(S)Cc3ccccc3)C(=O)N12	CHEMBL65545
OC(=O)C(Cc1ccc(cc1)-c1ccccc1)NC(=O)C1(CCCC1)NCP(O)(O)=O	CHEMBL329666
CCc1nnc(NC(=O)C2(CC(CCc3ccccc3)C(O)=O)CCCC2)s1	CHEMBL379065
CCOC(=O)C(C)NC(Cc1ccc(cc1)-c1cccc(Cl)c1)C(=O)Nc1nnn[nH]1	CHEMBL3695448
OC(=O)CN1c2ccccc2CCCC(NC(=O)C(S)Cc2ccccc2)C1=O	CHEMBL50814
CC(C)C(NC(=O)C(CC#Cc1ccccc1F)NCP(O)(O)=O)C(O)=O	CHEMBL36382
CC(C)C(S)C(=O)NC(Cc1ccc(cc1)-c1ccccc1)C(O)=O	CHEMBL269997
NCCCCC(NC(=O)C(Cc1ccccc1)CP(O)(=O)C(Cc1ccccc1)NC(=O)OCc1ccccc1)C(O)=O	CHEMBL27111
COCCC(NC1(CCCC1)C(=O)NC(Cc1nc(no1)-c1ccccc1)C(O)=O)C(O)=O	CHEMBL1778536
CC(OC1OC(CO)C(O)C(O)C1NC(C)=O)C(NC(=O)C(Cc1ccccc1)NC(=O)C(CCCNC(N)=N)NC(=O)C(N)CCC(N)=O)C(=O)NC(CCCNC(N)=N)C(O)=O	CHEMBL3633455
OP(O)(=O)CNC(CC#Cc1ccc(F)cc1F)C(=O)NCCc1cccc2ccccc12	CHEMBL290868
OP(O)(=O)C(Cc1ccc2OCOc2c1)NC(Cc1ccc(cc1)-c1ccccc1)c1nnn[nH]1	CHEMBL268473+CHEMBL273489
CC(NC(=O)C(CS)Cc1ccccc1)C(O)=O	CHEMBL51715+CHEMBL86201+CHEMBL165980
OC(=O)C(Cc1ccccc1)NC(=O)C(Cc1ccccc1)NC(=O)CC#N	CHEMBL80950+CHEMBL335046
OC(=O)C(Cc1ccc(cc1)-c1ccccc1)NCP(O)(O)=O	CHEMBL273216+CHEMBL288364
CC(CC(Cc1ccc(cc1)-c1ccccc1)NC(=O)c1nnn(Cc2ccccc2)n1)C(=O)OCc1ccccc1	CHEMBL3970744
CCCC(CC1(CCCC1)C(=O)NC1CCCCC1CCC)C(O)=O	CHEMBL211529
COc1ccc(CNC(=O)c2cc(cnc2-c2cccnc2)-c2cc(Cl)cc(Cl)c2)cc1OC	CHEMBL3099978+CHEMBL3099899
OP(O)(=O)C(Cc1ccc2ccccc2c1)NC(Cc1ccc(cc1)-c1ccccc1)c1nnn[nH]1	CHEMBL273244+CHEMBL415967
OC(CN(CC1CCCCC1)C(=O)NC(Cc1ccc(O)cc1)C(O)=O)C(O)=O	CHEMBL402569
CC(C(CS)C(=O)NC(Cc1ccc(O)cc1)C(O)=O)c1ccc(F)c(F)c1	CHEMBL11607

CCCC(NC1(CCCC1)C(=O)NC(Cc1nc(CC(C)C)co1)C(O)=O)C(O)=O	CHEMBL1778528
CC(C)CC(NC(=O)C(CC#Cc1ccc(F)cc1F)NCP(O)(O)=O)C(O)=O	CHEMBL289150
OC(=O)C(Cc1ccc(cc1)-c1ccccc1)NC(=O)C(S)C1CCC1	CHEMBL257229
OC(=O)C(Cc1ccc(cc1)-c1ccccc1)NC(=O)C(Cc1ccc2OCOc2c1)NCP(O)(O)=O	CHEMBL328463
CCCC(NC(=O)C(CS)C(C)c1ccccc1)C(O)=O	CHEMBL275690
CC(CS)C(=O)NC(CSCc1ccc(cc1)N(C)C)C(O)=O	CHEMBL457317+CHEMBL457319+CHEMBL507175
OC(=O)C(Cc1ccc(cc1)-c1ccccc1)NC(=O)CS	CHEMBL258333
CC(C)CC(NC(=O)C(CS)Cc1ccccc1)C(O)=O	CHEMBL51428+CHEMBL442337
CC(NC(=O)C(CS)C(C)c1ccc(F)c(F)c1)C(O)=O	CHEMBL276008
OP(O)(=O)CNC(CC#Cc1ccccc1F)C(=O)NCCc1ccc(Br)cc1	CHEMBL417507
CCCCCC(CC(=O)NO)C(=O)NC(C(C)C)C(=O)N1CCCC1CO	CHEMBL67311+CHEMBL117720+CHEMBL308333+CHEMBL1563884
ONC(=O)C(Cc1ccccc1)C(=O)NCC(O)=O	CHEMBL17107
NC(Cc1ccccc1)C(=O)NC(CO)C(=O)NC(CCCNC(N)=N)C(O)=O	CHEMBL2029393
NC(CCC(N)=O)C(=O)NC(CCCNC(N)=N)C(=O)NC(Cc1ccc(F)cc1)C(=O)NC(CO)C(=O)NC(CCCNC(N)=N)C(O)=O	CHEMBL2021931+CHEMBL2022234
OC(=O)CNC(=O)C(Cc1ccccc1)CP(O)(=O)C(Cc1ccccc1)NC(=O)OCc1ccccc1	CHEMBL282158
OC(=O)C(Cc1ccccc1)N1C(=S)SC(=Cc2ccc(cc2)C(O)=O)C1=O	CHEMBL3263104+CHEMBL3263101+CHEMBL3263103
CC(NC(=O)C(N)CCC(N)=O)C(=O)NC(Cc1ccccc1)C(=O)NC(CO)C(=O)NC(CCCNC(N)=N)C(O)=O	CHEMBL2022227
CC(C)CC1CC(=O)N(C(CO)C(O)=O)C1=O	CHEMBL3221958
OP(O)(=O)CNC(CC#Cc1ccccc1F)c1nnn[nH]1	CHEMBL40028
CSCCC(N)CSSCC(Cc1ccccc1)C(=O)NC(Cc1ccccc1)C(=O)OCc1ccccc1	CHEMBL1160525+CHEMBL1160959
CC(C)CC(NC(=O)C(CC#Cc1ccccc1F)NCP(O)(O)=O)C(O)=O	CHEMBL290256
NC(=O)C(CCC(O)=O)NC(=O)C(CCC(O)=O)NC(=O)C(Cc1cc(no1)-c1ccc(cc1)-c1cccc(Cl)c1)CP(O)(=O)c1ccc(Br)cc1	CHEMBL507420
CC(C)CC(NC(=O)C(CC#Cc1ccc(F)cc1F)NCP(O)(O)=O)C(=O)NC(C)C(O)=O	CHEMBL147847
CCC(C(CS)C(=O)NC(Cc1ccc(O)cc1)C(O)=O)c1ccccc1	CHEMBL267766

OC(=O)C1Cc2cccc3CCC(NC(=O)C4(S)CCCC4)C(=O)N1c23	CHEMBL99861
CC(=O)SC1CC(COCc2cc(F)c(F)cc2F)N(C1)C(=O)Oc1cccc2OCCOc12	CHEMBL191879
CC(NC(Cc1ccc(cc1)-c1cc(Cl)ccc1Cl)C(=O)Nc1nnn[nH]1)C(=O)OC(C)(C)C	CHEMBL3695449
OC(CN(CCN1C(=O)c2cccc3cccc(C1=O)c23)C(=O)NC(Cc1ccc2ccccc2c1)C(O)=O)C(O)=O	CHEMBL271718
OC(=O)C1CCCCCc2ccccc2CC(CS)C(=O)N1	CHEMBL357311+CHEMBL342091
CC(C)CN(CC(O)C(O)=O)C(=O)NC(Cc1ccc2ccccc2c1)C(O)=O	CHEMBL450935
OP(O)(=O)C(Cc1ccccc1)NC(Cc1ccc(cc1)-c1ccccc1)c1nnn[nH]1	CHEMBL276872+CHEMBL418704
CCCCC(S)C(=O)NC1CCc2ccccc2N(CC(O)=O)C1=O	CHEMBL299875
COC(=O)C(CC(C)C)NC(=O)NC(CO)C(O)=O	CHEMBL3221961+CHEMBL3221962
OP(O)(=O)CNC(CC#Cc1ccccc1F)C(=O)NCc1ccccc1	CHEMBL37861
OC(=O)CNC(=O)C(CS)Cc1ccccc1	CHEMBL10247+CHEMBL61512+CHEMBL298827
CCC(C)C(S)C(=O)NC(Cc1ccc(cc1)-c1ccccc1)C(O)=O	CHEMBL252391+CHEMBL269996
CC(C(CS)C(=O)NC(COCc1ccccc1)C(O)=O)c1ccccc1	CHEMBL11236
NC(CCC(N)=O)C(=O)NC(CCCNC(N)=N)C(=O)NC(Cc1ccccc1)C(=O)NC(CO)C(=O)NC(CCCNC(N)=N)C(O)=O	CHEMBL2022225+CHEMBL2022238+CHEMBL3633447
NCCCCC(NC(=O)C(N)CCC(N)=O)C(=O)NC(Cc1ccccc1)C(=O)NC(CO)C(=O)NC(CCCNC(N)=N)C(O)=O	CHEMBL3633449
Cc1ccc(CC(NC(=O)C(CCCNC(N)=N)NC(=O)C(N)CCC(N)=O)C(=O)NC(CO)C(=O)NC(CCCNC(N)=N)C(O)=O)cc1	CHEMBL2024269
NC(=O)CC(NC(=O)C(Cc1ccccc1)CP(O)(=O)C(Cc1ccccc1)NC(=O)OCc1ccccc1)C(O)=O	CHEMBL27842
OC(=O)CN1c2ccccc2CCC(NC(=O)C(CC#Cc2ccc(F)cc2F)NCP(O)(O)=O)C1=O	CHEMBL446393
NC(CCC(N)=O)C(=O)NC(CCCNC(N)=N)C(=O)NC(Cc1ccc(I)cc1)C(=O)NC(CO)C(=O)NC(CCCNC(N)=N)C(O)=O	CHEMBL2022236
CC(C)CCC(NC(CCc1ccccc1)C(O)=O)C(=O)NC(Cc1ccc(cc1)-c1ccccc1)C(O)=O	CHEMBL1778526
CC(C)C(NCP(O)(O)=O)C(=O)NC(Cc1ccc(cc1)-c1ccccc1)C(=O)NC(Cc1ccc(O)cc1)C(O)=O	CHEMBL329894
CC(C)(C)CC(S)C(=O)NC(Cc1ccc(cc1)-c1ccccc1)C(O)=O	CHEMBL271223

OC(=O)C(Cc1c[nH]c2ccccc12)NC(=O)C1CCCC1P(O)(=O)C(Cc1ccccc1)NC(=O)OCc1ccccc1	CHEMBL571716+CHEMBL2153740
CC(NC(=O)C(CCCNC(N)=N)NC(=O)C(N)CCC(N)=O)C(=O)NC(CO)C(=O)NC(CCCNC(N)=N)C(O)=O	CHEMBL2022228
CC(NC(=O)C(CS)C(C)c1ccc2OCOc2c1)C(O)=O	CHEMBL11620
OC(=O)CCNC(=O)C(Cc1ccc(cc1)-c1ccccc1)NC(CCc1ccccc1)C(O)=O	CHEMBL1829584
COCCC(CC1(CCCC1)C(=O)NC1(CO)Cc2ccccc2C1)C(O)=O	CHEMBL225084+CHEMBL389703
Fc1cc(F)c(COCC2CC(CN2C(=O)Oc2cccc3OCCOc23)SC(=O)c2ccccc2)cc1F	CHEMBL193490
NC(CCC(N)=O)C(=O)NC(CCCNC(N)=N)C(=O)NC(Cc1ccc(cc1)C(=O)c1ccccc1)C(=O)NC(CO)C(=O)NC(CCCNC(N)=N)C(O)=O	CHEMBL2024270
CCCCC(NCP(O)(O)=O)C(=O)NC(Cc1ccc(cc1)-c1ccccc1)C(O)=O	CHEMBL313419
OC(=O)C(Cc1ccccc1)NC(=O)C(CC#Cc1ccccc1F)NCP(O)(O)=O	CHEMBL37542
OC(=O)C1Cc2cccc3CCC(NC(=O)C(S)C4CCCCC4)C(=O)N1c23	CHEMBL329474
OC(=O)C(Cc1ccccc1)NC(=O)C(CC(=O)CC#N)Cc1ccccc1	CHEMBL80243
OC(=O)C(Cc1ccc(cc1)-c1ccccc1)NC(=O)C(Cc1ccccc1O)NCP(O)(O)=O	CHEMBL329136
NC(CCC(N)=O)C(=O)NC(CCCNC(N)=N)C(=O)N(CC(=O)NC(CO)C(=O)NC(CCCNC(N)=N)C(O)=O)Cc1ccccc1	CHEMBL2024271
CC(NC(Cc1ccc(cc1)-c1cccc(Cl)c1)C(=O)Nc1cc(O)no1)C(O)=O	CHEMBL3695451
NC(CC(N)=O)C(=O)NC(CCCNC(N)=N)C(=O)NC(Cc1ccccc1)C(=O)NC(CO)C(=O)NC(CCCNC(N)=N)C(O)=O	CHEMBL2029396
OC(=O)Cc1csc(NC(=O)C(CC(COc2ccccc2)C(O)=O)Cc2ccc(cc2)-c2ccccc2)n1	CHEMBL34427+CHEMBL34541
CC(C)CN(CCP(O)(O)=O)C(=O)NC(Cc1ccc2ccccc2c1)C(O)=O	CHEMBL257251
OC(=O)C1Cc2cccc3CCC(NC(=O)C(CS)Cc4ccc(cc4)-c4ccccc4)C(=O)N1c23	CHEMBL2112402
CNC(=O)C(Cc1c[nH]cn1)NC(=O)CN(CCCc1ccccc1)CC(O)=O	CHEMBL3235416
OC(=O)CC(NC(=O)C(Cc1ccccc1)CP(O)(=O)C(Cc1ccccc1)NC(=O)OCc1ccccc1)C(O)=O	CHEMBL26814
CCCC(CC1(CCCC1)C(=O)NCc1ccc2OCOc2c1)C(O)=O	CHEMBL386053
OC(=O)C1CCCC2SCCC(NC(=O)C(S)Cc3ccccc3)C(=O)N12	CHEMBL289556
CCC(C)C(NC(=O)C(Cc1ccccc1)CP(O)(=O)C(Cc1ccccc1)NC(=O)OCc1ccccc1)C(O)=O	CHEMBL24919
CC(C(CS)C(=O)NC(Cc1ccc(O)cc1)C(O)=O)c1ccc(O)cc1	CHEMBL11227
CCC(S)C(=O)NC(Cc1ccc(cc1)-c1ccccc1)C(O)=O	CHEMBL257726

NC(CS)C(=O)NC(CCC(N)=O)C(=O)NC(CCCNC(N)=N)C(=O)NC(Cc1ccccc1)C(=O)NC(CO)C(=O)NC(CCCNC(N)=N)C(O)=O	CHEMBL2029399
CCCC(CC1(CCCC1)C(=O)NC1CCN(Cc2ccccc2)C1)C(O)=O	CHEMBL377041
OC(=O)CCC(NC(=O)C(Cc1ccccc1)CP(O)(=O)C(Cc1ccccc1)NC(=O)OCc1ccccc1)C(O)=O	CHEMBL25154+CHEMBL28153
OP(O)(=O)CNC(Cc1ccc(cc1)-c1ccccc1)c1nn[nH]n1	CHEMBL290698
CC(NCP(O)(O)=O)C(=O)NC(Cc1ccc(cc1)-c1ccccc1)C(O)=O	CHEMBL91218
CCCc1cnc(nc1)N1CC(S)CC1CNCc1cc(F)ccc1F	CHEMBL194651
CC(NC(=O)C(CS)Cc1ccccc1)C(=O)N1CCCC1C(O)=O	CHEMBL50203+CHEMBL299181
CCCN1CCCC(NC(=O)C(S)Cc2ccccc2)C(=O)N1CC(O)=O	CHEMBL61566
NC(CCC(N)=O)C(=O)NC(CCCNC(N)=N)C(=O)NC(Cc1ccccc1)C(=O)NC(CO)C(=O)NC(CCCNC(N)=O)C(O)=O	CHEMBL3633452
COC(=O)C(CC(C)C)CC(=O)NC(CO)C(O)=O	CHEMBL3221955+CHEMBL3221954+CHEMBL3221957+CHEMBL3221956
OC(=O)CC(NC(=O)C(CS)Cc1ccccc1)C(O)=O	CHEMBL3298934
CCCC(CC1(CCCC1)c1nnc(Cc2ccccc2)o1)C(O)=O	CHEMBL379950
CC(C(CS)C(=O)NC(Cc1ccc(O)cc1)C(O)=O)c1ccccc1C	CHEMBL416522
CC(O)C(NC(=O)C(Cc1ccccc1)NC(=O)C(CCCNC(N)=N)NC(=O)C(N)CCC(N)=O)C(=O)NC(CCCNC(N)=N)C(O)=O	CHEMBL2029397
NC(CCC(N)=O)C(=O)NC(CCCNC(N)=N)C(=O)NC(C(=O)NC(CO)C(=O)NC(CCCNC(N)=N)C(O)=O)c1ccccc1	CHEMBL2024267
OC(=O)CCNC(=O)C(CS)Cc1ccccc1	CHEMBL51466+CHEMBL196902
CC(C(CS)C(=O)NC(Cc1ccc(O)cc1)C(O)=O)c1ccc(F)cc1	CHEMBL273724
OC(=O)C(Cc1ccc(cc1)-c1ccccc1)NC(=O)C(S)C1CCCCC1	CHEMBL257026
OC(=O)C1Cc2ccccc2CCCCCCCC(CS)C(=O)N1	CHEMBL423574+CHEMBL433702
CC(CC(Cc1ccc(cc1)-c1ccccc1)N=C(O)c1cc(ncn1)C(O)=O)C(O)=O	CHEMBL3929999
CCCNc1ccnc(NC(=O)C2(CC(CCC)C(O)=O)CCCC2)n1	CHEMBL211897
NC(=N)NCCCC(NC(=O)C(Cc1ccccc1)CP(O)(=O)C(Cc1ccccc1)NC(=O)OCc1ccccc1)C(O)=O	CHEMBL24910+CHEMBL28215
CCCCC(CC(Cc1ccc(cc1)-c1ccccc1)C(=O)Nc1nc(CC(O)=O)cs1)C(O)=O	CHEMBL37527

OP(O)(=O)C(Cc1cccc2OCOc12)NC(Cc1ccc(cc1)-c1ccccc1)c1nnn[nH]1	CHEMBL10055+CHEMBL276690
NC(CCC(N)=O)C(=O)NC(CCCNC(N)=N)C(=O)NC(Cc1cccc(F)c1)C(=O)NC(CO)C(=O)NC(CCCNC(N)=N)C(O)=O	CHEMBL2022233
CC(C)CN(CC(O)C(O)=O)C(=O)NC(Cc1c[nH]c2ccccc12)C(O)=O	CHEMBL255568
OCC(NC(=O)C(CS)Cc1ccsc1)C(O)=O	CHEMBL3298936
OC(=O)C(Cc1ccc(cc1)-c1ccccc1)NC(=O)C(S)Cc1ccccc1	CHEMBL350414+CHEMBL163454
CCC(C)C(S)C(=O)NC(Cc1cccc(OCc2ccccc2)c1)C(O)=O	CHEMBL254495
OP(O)(=O)CNC(CC#Cc1ccc(F)cc1F)C(=O)NCCCc1ccccc1	CHEMBL290332
OC(=O)C(Cc1c[nH]c2ccccc12)NC(=O)C(Cc1cc(no1)-c1ccccc1)CP(O)(=O)C(Cc1ccccc1)NC(=O)OCc1ccccc1	CHEMBL570843+CHEMBL577753+CHEMBL577754
OP(O)(=O)C(Cc1cccc2ccccc12)NC(Cc1ccc(cc1)-c1ccccc1)c1nnn[nH]1	CHEMBL10251+CHEMBL9963
Cc1ccccc1C(CC(O)=O)NC(=O)c1cc(OCC2CC2)n(n1)-c1ccccc1F	CHEMBL2171398
CC(C)(C)C(NCP(O)(O)=O)C(=O)NC(Cc1ccc(cc1)-c1ccccc1)C(O)=O	CHEMBL93206
OP(O)(=O)CNC(CC#Cc1ccccc1F)C(=O)NCCc1ccc(cc1)-c1ccccc1	CHEMBL39714
CC(C)CC(S)C(=O)NC1CCc2ccccc2N(CC(O)=O)C1=O	CHEMBL415932
CCCC(CC1(CCCC1)C(=O)Nc1cccc(CC)n1)C(O)=O	CHEMBL377415
OC(=O)CNC(=O)C(CS)=Cc1ccccc1	CHEMBL81232+CHEMBL312460
NC(CCC(N)=O)C(=O)NC(CCCNC(N)=N)C(=O)NC(Cc1ccc(O)cc1)C(=O)NC(CO)C(=O)NC(CCCNC(N)=N)C(O)=O	CHEMBL2024268
OP(O)(=O)CNC(CC#Cc1ccc(F)cc1F)C(=O)NCCc1ccccn1	CHEMBL37456
COc1cc(nn1-c1ccccc1F)C(=O)NC(CC(O)=O)c1ccccc1C	CHEMBL2171396
OP(O)(=O)CNC(CC#Cc1ccccc1F)C(=O)NCCc1ccccc1	CHEMBL39997
CC(S)C(=O)NC1CCc2ccccc2N(CC(O)=O)C1=O	CHEMBL50480
CCCC(CC1(CCCC1)C(=O)NCc1nnc(C)s1)C(O)=O	CHEMBL377618
OC(=O)C1Cc2cccc3CCC(NC(=O)CS)C(=O)N1c23	CHEMBL99132
OC1CC(N(C1)C(=O)C1CCCCCCC(CS)C(=O)N1)C(O)=O	CHEMBL124536+CHEMBL2204326
OC(=O)C(Cc1ccccc1)NC(=O)C(Cc1cc(no1)-c1ccccc1)CP(O)(=O)C(Cc1ccccc1)NC(=O)OCc1ccccc1	CHEMBL570718+CHEMBL567628

CC(NC(=O)C(Cc1ccc(cc1)-c1ccccc1)CP(O)(=O)C(N)c1ccccc1)C(O)=O	CHEMBL117394+CHEMBL1159942+CHEMBL1159927
CCC(C)C(S)C(=O)NC(Cc1cccc2ccccc12)C(O)=O	CHEMBL400527
OC(=O)C(Cc1ccc(O)cc1)NC(=O)C(CS)Cc1ccccc1	CHEMBL11495+CHEMBL310482
OC(=O)CCN1c2ccccc2OCC(NC(=O)C(S)Cc2ccccc2)C1=O	CHEMBL301751
CC(OCc1ccccc1)C(NCP(O)(O)=O)C(=O)NC(Cc1ccc(cc1)-c1ccccc1)C(O)=O	CHEMBL366503
OC(=O)C(Cc1c[nH]c2ccccc12)NC(=O)C(CC1CC(=NO1)c1ccc(cc1)C(F)(F)F)CP(O)(=O)C(Cc1ccccc1)NC(=O)OCc1ccccc1	CHEMBL570704
OC(=O)CC(NC(=O)C1(CS)CCCCC1)C(O)=O	CHEMBL3298941
OC(=O)CCN1c2ccccc2CCC(NC(=O)C(CS)Cc2ccccc2)C1=O	CHEMBL51438
CC(N)P(O)(=O)CC(Cc1ccc(cc1)-c1ccccc1)C(=O)NC(C)C(O)=O	CHEMBL420321+CHEMBL1159940+CHEMBL1159933
CCC(C)C(S)C(=O)NC(Cc1ccc(OCc2ccc(cc2)C(F)(F)F)cc1)C(O)=O	CHEMBL251809
O=C(Cc1ccc2nccnc2c1)N1CCCC1	CHEMBL487941
CCCC(CC1(CCCC1)C(=O)NC(C)c1ccccc1)C(O)=O	CHEMBL378050
OC(=O)C1Cc2cccc3CCC(NC(=O)C(S)Cc4ccc(cc4)-c4ccccc4)C(=O)N1c23	CHEMBL319744
OP(O)(=O)C(Cc1cccc2cccnc12)NC(Cc1ccc(cc1)-c1ccccc1)c1nnn[nH]1	CHEMBL10436+CHEMBL268761
CC(O)C(NCP(O)(O)=O)C(=O)NC(Cc1ccc(cc1)-c1ccccc1)C(O)=O	CHEMBL327799
CC(C)C(NC(C)C(O)=O)C(=O)NC(Cc1ccc(cc1)-c1ccccc1)C(O)=O	CHEMBL92632
COC(=O)c1ccccc1NC(=O)CN(C)C(=O)C1CC(S)CN1S(=O)(=O)c1ccc2ccccc2c1	CHEMBL177000
CCC(C)C(S)C(=O)NC(Cc1ccc(OCc2cc(cc(c2)C(F)(F)F)C(F)(F)F)cc1)C(O)=O	CHEMBL252009
CS(=O)CCC(N)CS	CHEMBL1159809+CHEMBL3298943+CHEMBL3307167
COc1ccc2c(OC3CC(N(C3)C(=O)C(NC(=O)OC3CCCC3)C(C)(C)C)C(=O)NC3(CC3C=C)C(O)=O)cc(nc2c1Br)-c1csc(NC(=O)C(C)C)n1	CHEMBL1241348+CHEMBL3544915
CCCC(CC1(CCCC1)C(=O)NC1(CO)CCCC1)C(O)=O	CHEMBL212065
OP(O)(=O)CNC(CC#Cc1ccc(F)cc1F)C(=O)NCCc1ccc(cc1)-c1ccccc1	CHEMBL40168
CC(C)CC(NC(=O)C(Cc1ccccc1)CP(O)(=O)C(Cc1ccccc1)NC(=O)OCc1ccccc1)C(O)=O	CHEMBL27110
CC(C)C(S)C(=O)NC1CCc2cccc3CC(N(c23)C1=O)C(O)=O	CHEMBL98876

OC(=O)C(Cc1ccc(O)cc1)NC(=O)C(Cc1cc(no1)-c1ccccc1)CP(O)(=O)C(Cc1ccccc1)NC(=O)OCc1ccccc1	CHEMBL570953+CHEMBL570732
OC(=O)CN1c2ccccc2SCC(NC(=O)C(CS)Cc2ccccc2)C1=O	CHEMBL51780
OP(O)(=O)CNC(CC#Cc1ccc(F)cc1F)C(=O)NCCc1ccc2ccccc2c1	CHEMBL37078
CC(C)CC(NC(=O)C(Cc1ccccc1)NC(=O)c1cnccn1)B(O)O	CHEMBL325041+CHEMBL524664+CHEMBL1530+CHEMBL3559830
OC(=O)C(Cc1ccc(O)cc1)NC(=O)C(Cc1ccccc1)CP(O)(=O)C(Cc1ccccc1)NC(=O)OCc1ccccc1	CHEMBL278754
CC(NC(Cc1ccc(cc1)-c1ccccc1)C(=O)NC1=NNNN1)C(O)=O	CHEMBL3695450
COCCC(CC1(CCCC1)C(=O)NCCc1ccc(Cl)cc1)C(O)=O	CHEMBL225085+CHEMBL390170
OC(=O)C1CCCC2N1C(=O)C(Cc1ccccc21)NC(=O)C(S)Cc1ccccc1	CHEMBL78429+CHEMBL198316+CHEMBL353341
NC(CCC(N)=O)C(=O)NC(CCCNC(N)=N)C(=O)NC(Cc1c(F)c(F)c(F)c(F)c1F)C(=O)NC(CO)C(=O)NC(CCCNC(N)=N)C(O)=O	CHEMBL2022237
NC(CCC(N)=O)C(=O)NC(CCCNC(N)=N)C(=O)NC(Cc1ccc(Cl)cc1)C(=O)NC(CO)C(=O)NC(CCCNC(N)=N)C(O)=O	CHEMBL2022235
OC(=O)C(CCc1ccccc1)NC(Cc1ccc(cc1)-c1cccc(Cl)c1)C(=O)NC1=NNNN1	CHEMBL3695453
OC(=O)CN1c2ccccc2OCC(NC(=O)C(S)Cc2ccccc2)C1=O	CHEMBL54036
CCCC(CC1(CCCC1)C(=O)NCCc1ccc(Cl)cc1)C(O)=O	CHEMBL211610
CC(C(CS)C(=O)NC(Cc1ccc(O)cc1)C(O)=O)c1ccc(N)cc1	CHEMBL273672
OC(=O)Cn1ccnc1NC(=O)C(CC(COc1ccccc1)C(O)=O)Cc1ccc(cc1)-c1ccccc1	CHEMBL34369
OC(=O)C(COc1ccccc1)CC(Cc1ccc(cc1)-c1ccccc1)C(=O)Nc1nn[nH]n1	CHEMBL289130
CCC(C)C(S)C(=O)NC(Cc1ccc(OCc2ccc(F)c(F)c2)cc1)C(O)=O	CHEMBL251808
OC(=O)C1CCCC2SCCCC(NC(=O)C(S)Cc3ccccc3)N12	CHEMBL372307
OC(=O)C(Cc1c[nH]c2ccccc12)NC(=O)C(CC1CC(=NO1)c1ccc(cc1)-c1ccccc1)CP(O)(=O)C(Cc1ccccc1)NC(=O)OCc1ccccc1	CHEMBL570722
CC(C(NCP(O)(O)=O)C(=O)NC(Cc1ccc(cc1)-c1ccccc1)C(O)=O)c1ccccc1	CHEMBL329530
OC(=O)C(Cc1ccc(cc1)-c1ccccc1)NC(=O)C(Cc1ccc(cc1)-c1ccccc1)NCP(O)(O)=O	CHEMBL418959
CCCC(CC1(CCCC1)C(=O)Nc1nnc(CC)s1)C(O)=O	CHEMBL212572+CHEMBL211500+CHEMBL378763
OC(=O)C(CC#Cc1ccc(F)cc1F)NCP(O)(O)=O	CHEMBL39812

CC(C)CC(NP(O)(=O)OC1OC(C)C(O)C(O)C1O)C(=O)NC(Cc1c[nH]c2ccccc12)C(O)=O	CHEMBL41289+CHEMBL79104+CHEMBL418012+CHEMBL479579+CHEMBL563250+CHEMBL1742968+CHEMBL1318812+CHEMBL1257059+CHEMBL1512679+CHEMBL1742380+CHEMBL2028192+CHEMBL2028371+CHEMBL2442111+CHEMBL3040712
CCC(C)C(S)C(=O)NC(Cc1ccc(Oc2ccccc2)cc1)C(O)=O	CHEMBL254282
CCCC(CC1(CCCC1)NC(=O)c1ccc(=O)n(Cc2ccccc2)c1)C(O)=O	CHEMBL424877
Cc1ccccc1C(CC(O)=O)NC(=O)c1cc(OCC(O)C(C)(C)C)n(n1)-c1ccccc1F	CHEMBL2171392+CHEMBL2171394
COc1ccc(cc1OC)C(C)C(CS)C(=O)NC(Cc1ccc(O)cc1)C(O)=O	CHEMBL11806
CC(C)C(NCP(O)(O)=O)C(=O)NC(Cc1ccc(cc1)-c1ccccc1)C(O)=O	CHEMBL89563
CC(C)(S)C(=O)NC(Cc1ccc(cc1)-c1ccccc1)C(O)=O	CHEMBL404044
COCCC(NC1(CCCC1)C(=O)NC(Cc1nnc(o1)-c1ccccc1)C(O)=O)C(O)=O	CHEMBL1778535
CC(C)CN(CC(O)C(O)=O)C(=O)NC(Cc1ccc(O)cc1)C(O)=O	CHEMBL270576
CCC(C)C(S)C(=O)NC1CCc2cccc3CC(N(c23)C1=O)C(O)=O	CHEMBL319836
CC(O)C(NC(=O)C(CS)Cc1ccsc1)C(O)=O	CHEMBL3298937
CC(C)C(NCP(O)(O)=O)C(=O)NC(Cc1ccc(cc1)-c1ccccc1)C(=O)N1CC(O)CC1C(O)=O	CHEMBL329372
COCCC(NC1(CCCC1)C(=O)NC(Cc1nc(C)c(o1)-c1ccccc1)C(O)=O)C(O)=O	CHEMBL1778533
CC(C)C(NC(=O)C(Cc1ccccc1)CP(O)(=O)C(Cc1ccccc1)NC(=O)OCc1ccccc1)C(O)=O	CHEMBL24923+CHEMBL27273
CC(CSC(C)=O)C(=O)Nc1ccccc1	CHEMBL322069
CCC(NC(Cc1ccc(cc1)-c1cccc(Cl)c1)C(=O)NC1=NNNN1)C(O)=O	CHEMBL3695454
CCCC(NC1(CCCC1)C(=O)NC(Cc1ncc(o1)-c1ccccc1)C(O)=O)C(O)=O	CHEMBL1778530
COCCC(NC1(CCCC1)C(=O)NC(Cc1ncc(o1)-c1ccccc1)C(O)=O)C(O)=O	CHEMBL1778532
CCC(C)C(S)C(=O)NCC(O)=O	CHEMBL401086
CC(C(CS)C(=O)NCC(O)=O)c1ccccc1	CHEMBL273748
CC(CC(Cc1ccc(cc1)-c1cccc(Cl)c1)NC(=O)c1ccc(o1)C(O)=O)C(O)=O	CHEMBL3975843
OC(=O)CN1c2ccccc2OCC(NC(=O)C(CS)Cc2ccccc2)C1=O	CHEMBL51389

CCC(C)C(S)C(=O)NC(Cc1ccccc1)C(O)=O	CHEMBL400526
OC(=O)CCN1c2ccccc2CCC(NC(=O)C(S)Cc2ccccc2)C1=O	CHEMBL301364
NC(CCC(N)=O)C(=O)NC(CCCNC(N)=O)C(=O)NC(Cc1ccccc1)C(=O)NC(CO)C(=O)NC(CCCNC(N)=N)C(O)=O	CHEMBL3633451
CC(C(CS)C(=O)NC(Cc1ccccc1)C(O)=O)c1ccccc1	CHEMBL2051772+CHEMBL2052007
CC(NC(=O)C(CO)NC(=O)C(Cc1ccccc1)NC(=O)C(CCCNC(N)=N)NC(=O)C(N)CCC(N)=O)C(O)=O	CHEMBL2022230
OC(=O)CN1c2ccccc2CCC(NC(=O)C(S)CC2CCCCC2)C1=O	CHEMBL299438
OC(=O)C1CCCCCCc2ccccc2CC(CS)C(=O)N1	CHEMBL146573+CHEMBL149841
OC(=O)C(Cc1ccc2c(c1)oc1ccccc21)NCP(O)(O)=O	CHEMBL34668+CHEMBL146936
OC(=O)C1CCCCCc2cccc(CC(CS)C(=O)N1)c2	CHEMBL147185+CHEMBL148840
CCCC(CC1(CCCC1)C(=O)Nc1ccccn1)C(O)=O	CHEMBL211596
CC(NC(=O)C(CC(=O)NO)Cc1ccccc1)C(O)=O	CHEMBL16046+CHEMBL85320+CHEMBL2079630
OC(=O)CC(NC(=O)C1(CS)CCCC1)C(O)=O	CHEMBL3298940
CC(C)CN(CP(O)(O)=O)C(=O)NC(Cc1ccc2ccccc2c1)C(O)=O	CHEMBL442455
COCCC(CC1(CCCC1)C(=O)NCCCc1ccc(F)cc1)C(O)=O	CHEMBL224502+CHEMBL224670
CCCC(CC1(CCCC1)C(=O)Nc1nnc(Cc2ccccc2)s1)C(O)=O	CHEMBL379935
CC(C)CC(S)C(=O)NC(Cc1ccc(cc1)-c1ccccc1)C(O)=O	CHEMBL271225
CCCC(CC1(CCCC1)C(=O)Nc1cc(C)ccn1)C(O)=O	CHEMBL211314
COc1ccc2C(CCc2c1)C(CS)C(=O)NC(Cc1c[nH]c2ccccc12)C(O)=O	CHEMBL60439+CHEMBL59762+CHEMBL304629+CHEMBL305089+CHEMBL305388
CC(C)(C)C(O)COc1cc(nn1-c1ccccc1F)C(=O)NC(CC(O)=O)c1ccc(Cl)cc1Cl	CHEMBL2171393+CHEMBL2171395
Oc1ccccc1C=Cc1ccc2ccccc2n1	CHEMBL127545+CHEMBL487942
CC(C)CC(N1CCOC(C(NC(=O)C(S)Cc2ccccc2)C1=O)c1ccccc1)C(O)=O	CHEMBL311838
OP(O)(=O)C(CCc1cccc2ccccc12)NC(Cc1ccc(cc1)-c1ccccc1)c1nnn[nH]1	CHEMBL285619

CC(S)(Cc1ccccc1)C(=O)NC1CCc2ccccc2N(CC(O)=O)C1=O	CHEMBL48534
CSCCC(NC(=O)C(CC#Cc1ccccc1F)NCP(O)(O)=O)C(O)=O	CHEMBL36753
CS(=O)(=O)NC(CCCCN)C(=O)NCC(CC1(CCCC1)C(=O)NC(Cc1ccc(O)cc1)C(O)=O)C(O)=O	CHEMBL42583
CC(C)CN(CC(O)C(O)=O)C(=O)NC(Cc1cccc2ccccc12)C(O)=O	CHEMBL409920
CC(NC(=O)C(CS)C(C)c1ccccc1)C(O)=O	CHEMBL2051773+CHEMBL2052008
NCCCCC(NC(=O)C(CO)NC(=O)C(Cc1ccccc1)NC(=O)C(CCCNC(N)=N)NC(=O)C(N)CCC(N)=O)C(O)=O	CHEMBL3633450
ONC(=O)CC(Cc1ccccc1)C(=O)NCC(O)=O	CHEMBL16779
OC(=O)C(Cc1c[nH]cn1)NC(=O)C(Cc1ccccc1)CP(O)(=O)C(Cc1ccccc1)NC(=O)OCc1ccccc1	CHEMBL26703
CC(NC(=O)C(CS)C(C)c1ccc(F)cc1)C(O)=O	CHEMBL11297
CCCC(NC1(CCCC1)C(=O)NC(Cc1nc(CC)co1)C(O)=O)C(O)=O	CHEMBL1778527
CCC(C)C(S)C(=O)NC(Cc1cccc(c1)-c1ccccc1)C(O)=O	CHEMBL401397
OC(=O)C(Cc1ccc(cc1)-c1ccccc1)NC(=O)C(NCP(O)(O)=O)C1CCCCC1	CHEMBL92638
CC(CC(Cc1ccc(cc1)-c1cccc(Cl)c1)N=C(O)c1cc(n[nH]1)C(O)=O)C(O)=O	CHEMBL3937696
OC(=O)CCN1c2ccccc2SCC(NC(=O)C(S)Cc2ccccc2)C1=O	CHEMBL299441
CC(CC(Cc1ccc(cc1)-c1cccc(Cl)c1)NC(=O)c1cc(O)no1)C(O)=O	CHEMBL3677671+CHEMBL3703477
CCCCC(CN(O)C=O)C(=O)NC(C(=O)N(C)C)C(C)(C)C	CHEMBL84969+CHEMBL93332+CHEMBL431210
OC(=O)C1CCCCc2ccccc2CC(CS)C(=O)N1	CHEMBL150222+CHEMBL346424
OP(O)(=O)CNC(CC#Cc1ccc(F)cc1F)C(=O)NCCc1ccccc1	CHEMBL289638
CCCC(CC1(CCCC1)C(=O)Nc1ncccc1CC)C(O)=O	CHEMBL379312
COC(=O)C(CC(C)C)C(=O)NC(CO)C(O)=O	CHEMBL3221959+CHEMBL3221960
CCCC(CC1(CCCC1)C(=O)Nc1nnc(CC2CC2)s1)C(O)=O	CHEMBL212377
CCC(C)C(NCP(O)(O)=O)C(=O)NC(Cc1ccc(cc1)-c1ccccc1)C(O)=O	CHEMBL90545
CC(C)C(NCP(O)(O)=O)C(=O)NC(Cc1c[nH]c2ccccc12)C(O)=O	CHEMBL92949
ONC(=O)CN1Cc2c(Cl)cccc2NS1(=O)=O	CHEMBL299946
CN(NC(=O)C1CC(S)CN1S(=O)(=O)c1ccc2ccccc2c1)S(=O)(=O)c1ccc(C)cc1	CHEMBL425383

CC(CSC(C)=O)C(=O)NCc1ccccc1	CHEMBL326801
OC(=O)CNC(=O)C(CC(COc1ccccc1)C(O)=O)Cc1ccc(cc1)-c1ccccc1	CHEMBL34884+CHEMBL37539+CHEMBL159508
CC(C)CC(NC(=O)C(NC(=O)c1cccc(n1)-c1ccccc1)C(C)O)B(O)O	CHEMBL270515
OC(=O)C(Cc1ccc(cc1)-c1ccccc1)NC(=O)C(S)C1CCCC1	CHEMBL404117
CCC(CC)CN(CC(O)C(O)=O)C(=O)NC(Cc1ccc2ccccc2c1)C(O)=O	CHEMBL257416
OC(=O)C(Cc1ccc(cc1)-c1ccccc1)NC(=O)C(Cc1ccccc1)NCP(O)(O)=O	CHEMBL93180
NC(CCC(N)=O)C(=O)NC(CCCNC(N)=N)C(=O)NC(CC(=O)NC(CO)C(=O)NC(CCCNC(N)=N)C(O)=O)c1ccccc1	CHEMBL2022239
CC(C)C(NC(=O)C(CS)C(C)c1ccccc1)C(O)=O	CHEMBL275980
CC(C)CN(CC(O)C(O)=O)C(=O)NC(Cc1ccc(cc1)-c1ccccc1)C(O)=O	CHEMBL409720
CCCC(CC1(CCCC1)NC(=O)C1Cc2ccccc2C1)C(O)=O	CHEMBL212409
NC(Cc1ccccc1)C(=O)NC(CCC(N)=O)C(=O)NC(CCCNC(N)=N)C(=O)NC(Cc1ccccc1)C(=O)NC(CO)C(=O)NC(CCCNC(N)=N)C(O)=O	CHEMBL2029398
OC(=O)C(CC#Cc1ccccc1F)NC(=O)C(CC#Cc1ccccc1F)NCP(O)(O)=O	CHEMBL39777
NC(CCC(N)=O)C(=O)NC(CCCNC(N)=N)C(=O)NC(Cc1ccccc1F)C(=O)NC(CO)C(=O)NC(CCCNC(N)=N)C(O)=O	CHEMBL2022231
OP(O)(=O)CNC(CC#Cc1ccc(F)cc1F)C(=O)NCCc1ccc(Br)cc1	CHEMBL416234
NC(CCC(N)=O)C(=O)NC(CCCNC(N)=N)C(=O)NC(Cc1ccccc1)C(=O)NC(CS)C(=O)NC(CCCNC(N)=N)C(O)=O	CHEMBL3633448
CC(CNC(=O)C(Cc1ccc(cc1)-c1ccccc1)NC(CCc1ccccc1)C(O)=O)C(O)=O	CHEMBL1829585
CC(S)C(=O)NC(Cc1ccc(cc1)-c1ccccc1)C(O)=O	CHEMBL257727
CC(NC(=O)C(CS)Cc1ccsc1)C(O)=O	CHEMBL3298935
OC(=O)C(Cc1c[nH]c2ccccc12)NC(=O)C(CC1CC(=NO1)c1ccccc1)CP(O)(=O)C(Cc1ccccc1)NC(=O)OCc1ccccc1	CHEMBL570842
O=c1cc(oc2ccccc12)N1CCOCC1	CHEMBL367315
CC(C)CC(NC(=O)C(CC#Cc1ccccc1)NCP(O)(O)=O)C(O)=O	CHEMBL288837
CCC(NC(=O)C(CS)C(C)c1ccccc1)C(O)=O	CHEMBL6915
OC(CN(Cc1ccc-2c(Cc3ccccc-23)c1)C(=O)NC(Cc1ccc2ccccc2c1)C(O)=O)C(O)=O	CHEMBL256895
NC(=O)CCC(NC(=O)C(Cc1ccccc1)CP(O)(=O)C(Cc1ccccc1)NC(=O)OCc1ccccc1)C(O)=O	CHEMBL27868
CC(NC(=O)C(Cc1ccccc1)NC(=O)C(CCCNC(N)=N)NC(=O)C(N)CCC(N)=O)C(=O)NC(CCCNC(N)=N)C(O)=O	CHEMBL2022229
CCCC(CC1(CCCC1)C(=O)N(C)c1ccc(=O)n(Cc2ccccc2)c1)C(O)=O	CHEMBL209441

CC(C)CN(CC(O)C(O)=O)C(=O)NC(Cc1ccc(O)c(O)c1)C(O)=O	CHEMBL258278
CCCCC(S)C(=O)NC(Cc1ccc(cc1)-c1ccccc1)C(O)=O	CHEMBL257270
COCCC(CC1(CCCC1)C(=O)NC1CC1c1ccc(OC)cc1)C(O)=O	CHEMBL223280+CHEMBL389061
CCCC(CC1(CCCC1)c1nnc(o1)-c1ccc(=O)n(Cc2ccccc2)c1)C(O)=O	CHEMBL212374
COc1ccccc1CC(NCP(O)(O)=O)C(=O)NC(Cc1ccc(cc1)-c1ccccc1)C(O)=O	CHEMBL93785
CC(O)C(NC(=O)C(CS)Cc1ccccc1)C(O)=O	CHEMBL3298860
OC(=O)CCNC(=O)C(Cc1ccccc1)NCC(O)=O	CHEMBL1829582
CC(C)C(NCP(O)(O)=O)C(=O)NC(CCc1ccccc1)C(O)=O	CHEMBL93562
CCC(C)C(S)C(=O)NC(C)C(O)=O	CHEMBL254900
OC(=O)C(CC1CCCCC1)NC(=O)C(CC#Cc1ccc(F)cc1F)NCP(O)(O)=O	CHEMBL39828
CC(=O)NC1C(O)C(O)C(CO)OC1OCC(NC(=O)C(Cc1ccccc1)NC(=O)C(CCCNC(N)=N)NC(=O)C(N)CCC(N)=O)C(=O)NC(CCCNC(N)=N)C(O)=O	CHEMBL3633454+CHEMBL3633453
CCCCC(NC(=O)C(CS)C(C)c1ccccc1)C(O)=O	CHEMBL11537
NCCc1c(NC(=O)C(CC#Cc2ccccc2F)NCP(O)(O)=O)[nH]c2ccccc12	CHEMBL290304
OC(=O)C(Cc1ccc2ccccc2c1)NC(=O)C(CC#Cc1ccccc1F)NCP(O)(O)=O	CHEMBL39887
OC(=O)C1Cc2cccc3CCC(NC(=O)C4(S)Cc5ccccc5C4)C(=O)N1c23	CHEMBL316595
CC(O)C(NC(=O)C(Cc1ccccc1)CP(O)(=O)C(Cc1ccccc1)NC(=O)OCc1ccccc1)C(O)=O	CHEMBL431691+CHEMBL1794047
COCCOCC(CC1(CCCC1)C(=O)NC1CCC(CC1)C(O)=O)C(O)=O	CHEMBL434492
OC(=O)CN1c2ccccc2CCC(NC(=O)C(CS)Cc2ccccc2)C1=O	CHEMBL51498
CC(C)(C)CN(CC(O)C(O)=O)C(=O)NC(Cc1ccc2ccccc2c1)C(O)=O	CHEMBL273142
ONC(=O)CN1Cc2c(Cl)cccc2NC1=O	CHEMBL298647
CC(CS)C(=O)N1CCCC1C(O)=O	CHEMBL27686+CHEMBL76577+CHEMBL161170+CHEMBL162360+CHEMBL269634+CHEMBL434965+CHEMBL1560
CC(CC(Cc1ccc(cc1)-c1ccccc1)N=C(O)c1c[nH]c(n1)C(O)=O)C(O)=O	CHEMBL3902042
NC(CCC(N)=O)C(=O)NC(CCCNC(N)=N)C(=O)NC(CCc1ccccc1)C(=O)NC(CO)C(=O)NC(CCCNC(N)=N)C(O)=O	CHEMBL2024266

4. Selection of compounds for *in vitro* tests after virtual screening

4.1 UNISTRA virtual hits

202 Compounds were selected for further *in vitro* studies. Key to score values:

- Global ACE Score 0 > top risk, if both models predict 1 with high confidence;
- Global ACE Score 1 > high risk: both models predict 1, and at least one of these predictions is high confidence;
- 2 > Medium risk: one model says 1 with high confidence, the other 0, but with low confidence OR both models predict 1 with low confidence;
- 3 > Low risk: opposite as above (predicted 1 with low confidence, 0 with high confidence);
- 4 > Conflicting: one model says 0 with high confidence and the other 1, with high confidence;
- 5 > Fuzzy: both models are low confidence, and they are in conflict;
- 6 > Likely selective: both models predict 0, and at least one of these predictions is high confidence;
- 7 > Top selective, if both models predict 0 with high confidence.

Table S4. Virtual hits obtained using UNISTRA pharmacophore search, 202 molecules in total.

Smiles	ID	Global Score
Cc1nonc1NC(=O)CCNC(=O)c1ccc(Br)cc1	Z991624914	6
CCOC(=O)c1oc2ccccc2c1COC(=O)C1CCN(CC1)S(=O)(=O)CC	Z98486128	6
Cc1cc(NC(=O)Cn2c(noc2=O)-c2cccc(Br)c2)no1	Z982276516	6
COc1cc(CN(C)C(=O)CSCCC(O)=O)ccc1OCc1ccccc1	Z97961252	7
OC(=O)CSc1ccc(cc1F)C(=O)C=Cc1cnn(c1)-c1ccccc1	Z95185503	2
COc1cc(CNC(=O)COCc2nc3ccccc3s2)ccc1OCc1ccccc1	Z91079372	6
COc1cc(CNC(=O)c2cccc3cccnc23)ccc1OCc1ccccc1	Z88236866	7
COc1cc(CNC(=O)c2c[nH]c3ccccc23)ccc1OCc1ccccc1	Z88236855	7
O=C(NCCCn1ccc2ccccc12)c1ccc(CN2CCCC2=O)cc1	Z87658563	1
CC(=O)NCCc1ccc(cc1)C(=O)COC(=O)CSc1ccc2CCCc2c1	Z82140103	6
Fc1ccc(cc1)N1CCC(CNC(=O)NCc2cccc(CN3CCCC3)c2)C1	Z819964746	2
O=C(CCN1CCOC1=O)NCc1cccc(OCc2ccccn2)c1	Z809000274	0
CCC=C(C)C(=O)NC(C)c1ccc(OCc2ccncc2)c(OC)c1	Z773299328	6
Cc1cc(C(=O)COC(=O)c2ccc(O)cc2O)c(C)n1CCc1cccs1	Z74253898	6
CC(NS(=O)(=O)c1ccc(Cl)c(Cl)c1)C(=O)NNC(=O)c1ccccc1O	Z68348244	7
O=C(CCc1ccc2ccccc2n1)OCC(=O)N1CCc2ccccc2C1	Z66318769	0
CCCNC(=O)NC(=O)CN(C)Cc1ccc(OCc2ccccc2)c(OC)c1	Z65193223	7
O=C(NCc1cccc(Cn2ccnc2)c1)c1cccc(Cn2ccnc2)c1	Z647198664	2
OC(=O)CCc1ccc(cc1)S(=O)(=O)Nc1ccccc1Cc1ccccc1	Z64439672	0
OC1=C(C(N(Cc2ccco2)C1=O)c1cccc(OCc2ccccc2)c1)C(=O)c1ccc(F)cc1	Z57781767	2
COc1cc(ccc1NC(=O)NC1(Oc2ccc(Cl)cc2O1)C(F)(F)F)[N+]([O-])=O	Z57336828	6
CS(=O)(=O)c1ccc2sc(NC(=O)NC3(Oc4ccc(Cl)cc4O3)C(F)(F)F)nc2c1	Z57269060	6

FC(F)Oc1cccc(NC(=O)NC2(Oc3ccc(Cl)cc3O2)C(F)(F)F)c1	Z56811532	2
CCCCCCCCCCCCCCCCOC(=O)C[n+]1csc(CCO)c1C	Z56797066	6
FC(F)(F)C1(NC(=O)Nc2cccc(c2)S(=O)(=O)N2CCCCC2)Oc2cc(Cl)c(Cl)cc2O1	Z56781802	5
OC(=O)CCCCCCCCC#CC#CCCc1ccccc1	Z56754421	1
Clc1ccc(C(=O)NC(=S)Nc2ccc(OCc3ccccc3)cc2)c(Cl)c1	Z56060429	1
Brc1ccccc1C(=O)NC(=S)Nc1ccc(OCc2ccccc2)cc1	Z56060415	0
Cc1ccc2c(Cl)cc(Cl)c(OCC(=O)Nc3ncc(Cl)cc3Cl)c2n1	Z54893251	7
COc1cc(ccc1OCc1ccncc1)C(C)NC(=O)C1CCCN1S(=O)(=O)c1ccccc1	Z512338858	7
O=C(COc1ccc(OCc2ccccc2)cc1)NN=C1CCc2ccccc12	Z49842624	1
Fc1ccc(COc2ccccc2C(=O)NN=Cc2ccc(OC(=O)c3cccnc3)cc2)cc1	Z49831792	2
Cc1cc2C(=NNC(=O)c3ccc(CSc4nc(C)cc(C)n4)cc3)C(O)=Nc2c(C)c1	Z49734224	6
Cc1cc2C(=NNC(=O)c3ccc(CSc4nncn4C)cc3)C(O)=Nc2c(C)c1	Z49733282	6
CCc1ccc(C=NNc2ccc(cc2[N+]([O-])=O)S(=O)(=O)Nc2ccc(C)cc2C)cc1	Z49608813	6
CN(C)c1ccc(C=NNc2ccc(cc2[N+]([O-])=O)S(=O)(=O)Nc2ccc(C)cc2C)cc1	Z49608568	6
CC(=NNc1ccc(Cl)c(Cl)c1)c1ccc(cc1)S(=O)(=O)NCc1ccco1	Z49570282	6
CC(=NNc1nc2ccccc2s1)c1ccc(cc1)S(=O)(=O)NCc1ccco1	Z49559500	6
COC(=O)C1CCCCC1CSCc1cccc(c1)C(=O)NCc1ccco1	Z4874253836	7
COCCNC(=O)C(=Cc1ccc(OCc2ccccc2)cc1)c1nc2ccccc2[nH]1	Z46552833	6
CCOc1ccc(OCc2ccc(cc2)C(=O)NN=C2CCCc3ccccc23)cc1	Z44852701	6
O=C(Cc1cn2ccsc2n1)NN=Cc1cccc(OCc2ccccc2)c1	Z44841256	2
CC(N1CCC(C(C1)NC(=O)C(F)(F)F)c1ccccc1)c1nc(no1)-c1ccc(Cl)cc1	Z4457797242	6
CCCS(=O)(=O)N1CCCC(C1)C(=O)NCc1ccc(Cn2ccnc2)cc1	Z437796564	6
Cc1cc(=O)c(nn1-c1cccc(c1)C(F)(F)F)C(=O)NCc1ccnc(OCc2ccccc2)c1	Z421650342	6
Fc1cc(F)cc(c1)C(=O)NCc1ccc(OCc2cccnc2)cc1	Z421641354	3
CC(NC(=O)COc1ccccc1)c1ccc(OCc2cccnc2)cc1	Z408793428	7
NC(CC1CCCCO1)C(=O)Nc1cccc(Cc2nc3ccccc3[nH]2)c1	Z4064247424	2
C(CNCc1ccc(o1)-c1ccccc1)CN1CCN(CC1)c1ncccn1	Z401881196	2
CCOC(=O)C1CCCN(C1)S(=O)(=O)NCc1ccnc(OC2CCCC2)c1	Z401495940	7
OC(C1CCOCC1)C(=O)NCC(Cc1ccc2ccccc2c1)C(O)=O	Z4008314831	0
Fc1cc2CCC(NS(=O)(=O)Cc3ccc(Br)c(F)c3)c2c(F)c1F	Z3950782188	6
Cc1ccc(CCNS(=O)(=O)N=S(C)(=O)c2cccc(Br)c2)nc1	Z3950742710	6
OC(C1CCN(CC1)C(=O)C(CCc1ccccc1)c1ccccc1)C(O)=O	Z3870793818	1

OCC1(CC1)c1ccc(NCc2cccc(OCCCC(O)=O)c2)cc1	Z3861452555	1
CCn1cc(cn1)C(O)CN(Cc1c[nH]c2cnccc12)Cc1ccccc1	Z3817371940	6
OC(=O)C(Cc1c[nH]c2ccccc12)NC(=O)CCCc1c[nH]c2ccccc12	Z381015304	0
COC(=O)CCC1CC(N(Cc2cccc(n2)-c2ccc(F)cc2)C1)C(O)=O	Z3790658555	7
O=C(CCCc1nc2ccccc2[nH]1)NCc1ccc(Cn2ccnc2)cc1	Z371524258	1
Clc1ccc(CCS(=O)(=O)NCc2cccc(OCc3ccccn3)c2)cc1	Z3710549528	1
CCOC(=O)C1CCCN(C1)S(=O)(=O)NCc1ccccc1COC(C)C	Z369245556	7
OC1CC(Cn2cc(Cl)cn2)CC1NCc1ccccc1-n1cncn1	Z3686110620	5
Cc1cc(C)nc(NNC(=O)C2CCCN2C(=O)c2ccc(Cl)c(Cl)c2)n1	Z365761012	6
Cn1cc(cn1)C1CC1CN(Cc1nc2cc(ccc2n1C)C(O)=O)C1CC1	Z3634556367	6
NCC(Cc1ccc(F)cc1)C(=O)NC(CCC(O)=O)Cc1ccccc1	Z3632728987	0
CNc1nc(NCC2(CO)COCC(C)N2)ncc1Cl	Z3560300025	6
CC1Cc2c(n[nH]c2C(C)O1)C(=O)NCCc1cnc2c(Cl)cnn2c1	Z3551917512	6
CC1Cc2c(n[nH]c2C(C)O1)C(=O)N1CC(Cc2ccc(Cl)cc2)C1	Z3538353534	7
NC(=O)NC(CC(=O)Nc1nc(cs1)C1CC1)c1cccc(Br)c1	Z352070678	2
OC(=O)CC1CC(CCO1)NC(CCc1ccccc1)CCc1ccccc1	Z3507960861	0
COc1ccccc1NC(=O)NCC(O)c1ccc(Cl)cc1	Z332420796	7
COC(=O)c1cccc(CS(=O)(=O)CCC(=O)NC(C)c2ccccc2)c1	Z327268322	6
COc1ccc(NC(=O)C(CCSC)NS(=O)(=O)c2ccc(F)c(Cl)c2)cn1	Z32447596	7
CC(NC(=O)c1ccco1)C(=O)NNC(=O)Cn1c(COc2ccc(Cl)cc2)nc2ccccc12	Z31930160	6
CC(O)CNCC(O)COc1cc(C)c(Cl)cc1C(C)C	Z31383461	7
CN(CC(O)COc1ccc2ccccc2c1)Cc1nc2ccccc2c(=O)[nH]1	Z31360148	6
NCc1ccc2nc(cn2c1)C(=O)NCCN1CCN(CC1)c1ccc(F)cc1	Z3104014597	1
CCOc1ccc(CN(CC(O)C(C)O)CC(OC)OC)cc1	Z3063574844	7
FC(F)(F)c1ccccc1NNC(=O)CSCc1ccc(Cl)cc1	Z30568063	1
CC(C)C(NC(=O)c1ccccc1)C(=O)NNC(=O)COc1cccc(Br)c1	Z30504785	7
OC(CNCc1cccc(OCc2ccccn2)c1)CC(O)c1ccccc1	Z2998546318	0
O=C(COc1ccc(OCc2ccccc2)cc1)Nc1cccc2ncccc12	Z29904566	0
CN(CC(=O)Nc1nc2c(C)cccc2s1)S(=O)(=O)c1ccc(Cl)cc1	Z29808778	6
COc1ccc(cc1)C1(NC(=O)n2nc(COc3ccc(Cl)cc3)nc2S1)C(F)(F)F	Z295883136	6
CCC1(CC)CNC(=O)C1NCc1nnc(o1)-c1cccc(Cl)c1	Z2955117788	6
CN(CC(=O)Nc1cc(C)ccc1C)C(=O)C(C)(C)Oc1ccc(Cl)cc1	Z29080161	7

CCCn1c(NC(=O)c2ccc(COc3ccc(cc3)C(C)(C)C)cc2)nc2ccccc12	Z29078101	7
CCCn1c(NC(=O)c2ccc(OCc3c(C)noc3C)cc2)nc2ccccc12	Z29076428	6
COc1cc(CNC(=O)c2cccc(C)n2)ccc1OCc1ccccc1	Z287228324	7
CC(C)COc1ccc(CC(=O)N2CCN(CCc3cnccn3)CC2)cc1	Z2785020620	6
Cc1ccc(o1)C(O)(CC(=O)OCC(=O)Nc1ccc(Cl)c(c1)C(F)(F)F)C(F)(F)F	Z278087056	6
OC(=O)C1CCC(N1)C(=O)NCc1nccn1CCCc1ccccc1	Z2765250173	1
CC(C)C(=O)N(C)Cc1ccc(cc1)C(=O)NCc1ccc(CC(O)=O)cc1	Z2755488642	7
OC(=O)C(CNC1CCCN(Cc2ccccc2)C1)C1CCOCC1	Z2723106678	0
Cc1c(NC(=O)N2CC(O)(C2)c2cccc(Br)c2)nnn1C	Z2655587988	6
Cc1n[nH]c(CC(=O)N2CCOC(COCC3CC3)C2)n1	Z2636519802	6
CN(C)C(=O)COC(=O)c1oc2ccccc2c1COc1ccccc1	Z25325633	6
Cc1ccc2nc(CSCC(=O)N3CCCC(CNCC(O)=O)C3)cn2c1	Z2528180770	6
CC(Sc1nnc([nH]1)-c1ccc(Cl)cc1)C(=O)Nc1ccccc1C#N	Z25220340	6
CC(Sc1nnc([nH]1)-c1ccc(Cl)cc1)C(=O)N1CCCC1	Z25220212	7
CC(NC(=O)CCCCC(O)=O)c1ccc(cc1)-c1ccc(cc1)N(C)C	Z2504126907	7
OC(=O)CS(=O)(=O)CCN1CCc2oc(cc2C1)-c1ccccc1	Z2476165556	1
CN(CCCNC(=O)C=Cc1ccc(cc1)N1CCCC1=O)Cc1ccccc1	Z244987194	5
CN(CCC(O)=O)S(=O)(=O)NCc1ccc(OCc2cccc(C)c2)cc1	Z2437641363	7
COc1cc(CNC(=O)C2CCCN(C2)C(=O)c2ccco2)ccc1OCc1ccccc1	Z241267688	7
COCCN1C(Nc2ccccc2C1=O)c1ccc(OCc2csc(C)n2)cc1	Z241053142	6
Cc1csc(NC(=O)C2CCCN2S(=O)(=O)c2ccc(Br)s2)n1	Z240193508	6
CCC(CC)(CNCc1ccc(OCc2ccccc2)cc1)C(O)=O	Z2396544499	7
OC(=O)CCC1CCCN(CC(=O)Nc2ccc(OCc3ccccc3)cc2)C1	Z2368944440	0
OC(=O)C(CNCc1ccc2CCCc2c1)Cc1cc(F)c(F)c(F)c1	Z2361945398	6
O=C(Cn1nc(oc1=O)-c1ccccc1)NCc1ccc(NC(=O)c2ccco2)cc1	Z236112994	1
Cc1nc2ncnn2c(C)c1CCC(=O)NCc1ccc(NC(=O)c2ccco2)cc1	Z236111464	6
CN(CC(=O)NNc1nc(C)cc(C)n1)S(=O)(=O)c1ccc(Cl)cc1	Z230156322	6
OC(=O)Cc1cccc(c1)C(=O)NCc1cccc(NC(=O)N2CCCC2)c1	Z2295857926	1
OC(=O)Cc1csc(SCC(=O)NCC(c2ccccc2)c2ccccc2)n1	Z227733986	1
CC(=O)NC(CC(=O)Nc1nc(cs1)-c1ccc[nH]1)c1ccc(Cl)cc1	Z225557196	6
Cc1ccc(cc1C)S(=O)(=O)NCCC(=O)OC(C(N)=O)c1ccccc1	Z224053630	6
CCCc1nnc(SC(C)C(=O)Nc2ccc(OCc3ccccc3)cc2)[nH]1	Z223710640	7

CCN(CC)CC#CC(C)OC(=O)c1cccc(Cn2ccnc2)c1	Z2235067421	6
OC(=O)CCC(=O)c1cccc(NS(=O)(=O)CCC2Cc3ccccc3C2)c1	Z2234684491	1
OC(=O)CCc1ccc(cc1)S(=O)(=O)NCc1ccc(Cn2ccnc2)cc1	Z223250114	2
OC(=O)C(Cc1c[nH]c2ccccc12)NC(=O)NCC(=O)NCc1ccccc1	Z223122390	0
CCCCCCCCCCCCCCCCCC(=O)NCCc1ccc(O)c(O)c1	Z221503626	1
CCN(CC)Cc1ccc(CNC(=O)NCc2ccc(cc2)C(=O)OC)cc1	Z211762780	7
O=C(Cc1csc(n1)-c1ccccn1)NCc1ccccc1Cn1ccnc1	Z203879208	1
O=C(CCCc1c[nH]c2ccccc12)NCc1ccc(Cn2ccnc2)cc1	Z203868760	1
CCOC(=O)C=CC(=O)OCC(=O)Nc1ccc(OCc2ccccc2)cc1	Z19772058	7
COc1ccc(CCn2c(C)cc(C(=O)COC(=O)c3ccco3)c2C)cc1	Z19719192	6
CC(OC(=O)CCC(=O)c1cccs1)C(=O)Nc1cccc2ccccc12	Z19708939	6
CC(=O)c1cccc(NC(=O)COC(=O)Cc2cccc3ccccc23)c1	Z19679542	6
CC1(CCN(CC1)C(=O)NCc1ncccc1S(C)(=O)=O)c1ccc(Cl)cc1	Z1942053400	6
CCCCc1ccc(CC(=O)NCc2ccccc2CS(=O)(=O)NC(C)C)cc1	Z192582430	6
CC(OC(=O)COc1cc(C)cc(C)c1)C(=O)Nc1cccc2ccccc12	Z19118899	7
COc1ccc(cc1F)C(NC(=O)NC1CC(F)(F)C1)C(F)(F)F	Z1866051850	7
CCCOc1ccc(CNCCCCCCC(O)=O)c(OCCC)c1	Z1860186257	7
Cc1cccc(c1)-c1nn(cc1CNCCCCCCC(O)=O)-c1ccccc1	Z1860186067	6
OC(=O)CCCCCCNCc1ccc(OCc2ccncc2)cc1	Z1860185700	0
COc1ccc2cc(CNC(Cc3ccccc3)C(O)=O)ccc2c1	Z1860181504	7
OC(=O)C(Cc1ccccc1)NCc1ccc(s1)-c1ccccc1F	Z1860180973	0
OC(=O)C(Cc1ccccc1)NCc1ccc(o1)-c1cccc(c1)[N+]([O-])=O	Z1860180782	1
COc1cc(CNC(Cc2ccccc2)C(O)=O)ccc1OCc1ccccc1	Z1860180405	6
OC(=O)C(Cc1ccccc1)NCc1ccc(OCc2ccncc2)cc1	Z1860180319	0
COC(=O)c1c(C)c(C)sc1NC(=O)CSc1nc2cc(Cl)ccc2s1	Z18552119	6
Brc1ccc2cc(OCC(=O)c3ccc(cc3)-c3ccccc3)ccc2c1	Z18526358	0
CC(Sc1nnnn1C)C(=O)Nc1ccc(Cc2nc3ccccc3s2)cc1	Z18520648	6
FC(F)(F)c1cccc(CC(=O)OCC(=O)Nc2cccc3ccccc23)c1	Z18496578	1
CCN1CN(CC(=O)NC(Cc2ccccc2)c2ccccc2)CC1=O	Z1846556075	7
CC(C)(Oc1ccc(Cl)cc1)C(=O)OCC(=O)NC(=O)NCC=C	Z18465056	6
CCOc1ccccc1NC(=O)COc1ccc(OCc2ccccc2)cc1	Z18460907	7
CCN(CC)CCc1ccc(NC(=O)CCc2cccc(c2)C(=O)NC)cc1	Z1842567997	7

O=C(COC(=O)CCCc1c[nH]c2ccccc12)Nc1ccc2CCCc2c1	Z18265105	0
Brc1ccc(cc1)-n1cnc(c1)C(=O)NC1(CCC1)c1nnc[nH]1	Z1820684529	5
C(Cc1ccccc1)Sc1nnc(CN2CCCC2)n1Cc1ccccc1	Z17435259	0
Fc1cccc(COc2ccc(CNC(=O)c3cc([nH]c(=O)c3)C3CC3)cc2)c1	Z1718523989	2
COc1cc(CNC(=O)C2CCCN(C2)C(=O)c2ccc(F)cc2)ccc1OCc1ccccc1	Z169659202	7
CCOC(=O)c1ccc(OCC(O)CN(C)Cc2nc3ccccc3s2)cc1	Z169113726	6
CC(OC(=O)c1ccc(CN2CCCC2=O)cc1)C(=O)c1cccc(Cl)c1	Z167924950	7
COC(=O)C(NCc1cccc(c1)C(=O)NCC(C)C)c1cccc(O)c1	Z1677385241	6
CN(CCCNC(=O)CSCC(=O)Nc1cccc(C)c1)Cc1ccccc1	Z167265400	7
Cc1cc2occ(CC(=O)OCC(=O)c3ccc(CCNS(C)(=O)=O)s3)c2cc1C	Z165919446	6
Cc1nccn1Cc1cccc(CNC2CCC(O)(CC2)c2ccccc2)c1	Z1642148817	6
Clc1ccccc1Cn1cc(CN2CCC(Cc3cccnc3)CC2)cn1	Z1632800398	1
CN(CC(=O)Nc1ccccc1Cl)C(=O)COC(=O)c1ccc(C)cc1O	Z16128538	7
Cc1nc(NC(=O)CSc2nc3cc(Cl)ccc3c(=O)n2CC=C)c(Cl)cc1Cl	Z16109574	6
CC(CNC(=O)c1cn(cn1)-c1ccc(Br)cc1)CC(O)=O	Z1609746819	7
OC(=O)CC(NC(=O)c1cccc(OCc2cscn2)c1)c1ccc(F)cc1F	Z1603641278	3
CC(CCCc1ccccc1)C(=O)NCc1cccc(Cn2ccnc2C)c1	Z1592818345	6
CC(CCCc1ccccc1)C(=O)NCc1cccc(Cn2ccnc2)c1	Z1592818296	6
CC(NCc1cccc(CNC(C)c2cccc(O)c2)c1)c1cccc(O)c1	Z1589345472	7
O=S(=O)(Cc1ccccc1)Cc1cccc(Nc2ncncc2-c2ccccc2)c1	Z1587639456	1
CC(NC(=O)c1ccccc1Cl)C(=O)OCC(=O)NCCc1ccccc1	Z15810906	7
CC(OC(=O)CCCOc1cccc(C)c1)C(=O)Nc1cccc(c1)C(C)=O	Z15789613	7
Cc1ccc(F)c(CNC(c2ncon2)c2ccc(Cl)cc2)c1	Z1537376430	6
NC(=O)NCc1ccc(cc1)C(=O)NCC1CN(Cc2ccccc2)C(=O)C1	Z1530856063	2
O=C(NCc1cccc(CN2CCCC2)c1)N1CCN(CC2CCCC2)C(=O)C1	Z1510344616	0
Cc1c(sc2ccccc12)C(=O)COC(=O)Cn1cnc2ccccc2c1=O	Z150737296	6
CCOc1cccc(CC(=O)Nc2ccc(CCN3CCN(C)CC3)cc2)c1	Z1465787234	7
Cc1ccc(CCC2CCN(CC2)C(=O)CN2CCCC2C(O)=O)cc1	Z1459912954	6
OC(=O)C(Cc1ccccc1)NC(=O)C1CCCN(Cc2cccc(F)c2)C1	Z1455297863	0
CC1CN(Cc2ccc(cc2)C(=O)NCc2ccc(cc2)C(O)=O)CC(C)O1	Z1455162743	7
OC(=O)CCSCc1cccc(NC(=O)CNC(=O)Cc2cccc(F)c2)c1	Z1445292532	1
Cc1oc(CNC(=O)c2cccc(CN3CCCC3=O)c2)cc1C(O)=O	Z1445033742	6

OC(=O)c1cccc(CNC(=O)c2cccc(CN3CCCC3=O)c2)c1	Z1444931703	0
CCCCN(CC)Cc1cccc(CNS(=O)(=O)c2ccc(cc2)C(N)=O)c1	Z1398453834	7
O=C(COC(=O)CCc1c[nH]c2ccccc12)Nc1cccc2ccccc12	Z13758989	0
Cc1cc2nc([nH]c2cc1C)-c1ccc(CNC(=O)Cn2cnc3ccccc3c2=O)cc1	Z1362864815	6
COCC(C)(O)CNC(=O)c1cc(=O)c2cc(Cl)ccc2o1	Z1286284150	6
CCC(C)(O)CNC(=O)c1cccc(NS(=O)(=O)c2cc(C)ccc2C)c1	Z1286173685	7
CC(=O)CCc1ccc(OCC(=O)N2CCC(CN3CCCCC3)C2)cc1	Z1267336922	6
O=S(=O)(Cc1ccccc1)N1CCC(CC1)NCc1ccc2[nH]cnc2c1	Z1225756298	2
Cc1ccccc1COc1cccc(NC(=O)C2=CC=CN3CCS(=O)(=O)N=C23)c1	Z1183535759	6
O=C(NCc1ccc(Cn2ccccc2=O)cc1)c1ccc(Cn2ccnc2)o1	Z1171705391	5
O=C(NCC1CCN(C1)c1ccccc1)NCc1ccnc(OCC2CC2)c1	Z1166238502	2
NC(=O)C1CCCN(Cc2cccc(CNCc3cc(F)ccc3F)c2)C1	Z1156003558	3
CC(=O)Nc1cccc(OCCNC(=O)c2cccc(Cn3ccnc3)c2)c1	Z1129178234	6
O=C(NCCCOCc1ccccc1)c1ccc(CN2CCCC2=O)cc1	Z110108098	0
Cc1ccnc2nc(nn12)C(=O)NCc1ccc(cc1)-c1nc2ccccc2[nH]1	Z1094921216	6
CC(C)CCN1C(=O)c2ccc(cc2C1=O)C(=O)Nn1cnc2ccccc12	Z108516702	6
CCc1nonc1NC(=O)COc1ccc(Cl)c(C)c1	Z1080985856	6
NC(=O)C(NCc1cccc(c1)C(=O)OCc1ccccc1)c1ccccc1	Z1070886730	1
OC(CNc1nc(nc2ccccc12)-c1ccncc1)COc1ccc(I)cc1	Z1070421880	1
COC(=O)c1cc(ccc1Br)S(=O)(=O)N(C)CC(=O)Nc1cc(C)ccn1	Z1021113120	7

4.2 ENAMINE virtual hits

Top 196 compound were selected for further *in vitro* studies:

Table S5. Virtual hits obtained using ENAMINE docking.

**Smiles**	**ID**	**Docking Score**
Cc1c(sc2ncn(CC(=O)NCc3ccc(F)cc3)c(=O)c12)C([O-])=O	Z107063344	-1,031
[O-]C(=O)CC1SC(=O)N(Cc2ccccc2)C1=O	Z108565354	-1,116
[O-]C(=O)C1CC(=O)N(CCc2cccnc2)C1c1cccs1	Z1136435964	-1,059
OC(COc1ccc2oc(cc(=O)c2c1)C([O-])=O)COc1cccc2oc(cc(=O)c12)C([O-])=O	Z1187818721	-1,031
CCCCOc1ccc(cc1C)S(=O)(=O)Nc1ccc(cc1)C([O-])=O	Z1198524132	-1,133
[O-]C(=O)CCN1C(c2c(oc3ccccc3c2=O)C1=O)c1ccc(Cl)cc1	Z1213670676	-1,027

Cc1c(nnn1-c1cccc2C[NH+](Cc3ccccn3)CCc12)C([O-])=O	Z1263820424	-1,065
CN(C)c1cc(CNC(=O)C2C3CC(C=C3)C2C([O-])=O)c2ccccc2n1	Z1278589694	-1,109
[O-]C(=O)c1ccc(NS(=O)(=O)c2ccc3COCc3c2)c(F)c1	Z1341587211	-1,049
Cc1ccccc1-c1nc(cs1)C(=O)N1CCCC(C1)C([O-])=O	Z1443622330	-1,112
COc1ccc(cc1OC)N1CC(CC1=O)C(=O)N1CCCC(C1)C([O-])=O	Z1443624534	-1,120
[O-]C(=O)CCCNC(=O)c1csc(n1)-c1ccc(Cl)cc1	Z1443686640	-1,077
COc1ccc(cc1OC)N1CC(CC1=O)C(=O)NCCC([O-])=O	Z1443846609	-1,102
[O-]C(=O)C1CC1C(=O)NCc1cc(Cl)c2OCCCOc2c1	Z1443950799	-1,036
COC(C(C)NC(=O)c1ccc(s1)C([O-])=O)c1ccc(Cl)cc1	Z1444163443	-1,086
CC(NC(=O)c1cccc(n1)C([O-])=O)c1ccc(cc1)-c1cncnc1	Z1444189226	-1,076
[O-]C(=O)CN(C1CCOCC1)C(=O)CCNC(=O)c1ccc2ccccc2c1	Z1444669856	-1,101
Cc1cc2occ(CC(=O)N(CCC([O-])=O)C3CCOCC3)c2cc1C	Z1444689167	-1,039
[O-]C(=O)CC1(CCOCC1)NC(=O)c1ccc(CNC(=O)C2CCCC2)cc1	Z1444738651	-1,104
[O-]C(=O)CC1(CCC1)NC(=O)CCSc1ccccc1Cl	Z1444775888	-1,052
[O-]C(=O)c1cccc(CNC(=O)Cc2c[nH]c3cc(F)ccc23)c1	Z1444935835	-1,062
CC(NC(=O)CCC1Cc2ccccc2NC1=O)C([O-])=O	Z1445006666	-1,118
Cc1oc(CNC(=O)CSc2ccccc2C)cc1C([O-])=O	Z1445031591	-1,037
CC(CN(C)C(=O)\C=C\c1cccc(OCc2ccccc2)c1)C([O-])=O	Z1445094680	-1,157
Cc1cc(OCC([O-])=O)ccc1NC(=O)C1CC1c1ccc(F)cc1F	Z1445129833	-1,053
[O-]C(=O)C1CN(CCO1)C(=O)c1csc(n1)-c1ccc(Cl)cc1	Z1445264400	-1,136
[O-]C(=O)C1CN(CCO1)C(=O)Cc1csc(n1)-c1ccccc1Cl	Z1445264658	-1,122
CCc1cccc2c(CC(=O)N3CCOC(C3)C([O-])=O)c[nH]c12	Z1445269023	-1,107
Cc1ccc(OCc2ccc(o2)C(=O)N2CCOC(C2)C([O-])=O)c(C)c1	Z1445271585	-1,102
[O-]C(=O)CSCCNC(=O)Cc1cc2ccccc2[nH]c1=O	Z1445301341	-1,040
[O-]C(=O)CC1CN(CCO1)C(=O)CCCCc1nc2ccccc2s1	Z1445440081	-1,137
CCc1ccc(cc1)-n1nc(C(=O)N2CCOC(CC([O-])=O)C2)c2CCCc12	Z1445442556	-1,138
CN(CC([O-])=O)C(=O)C1CCCN(C1)c1ncnc2sccc12	Z1454831732	-1,058
[O-]C(=O)C1CCCN1C(=O)CCC1NC(=O)N(C1=O)c1ccc(F)cc1F	Z1455181379	-1,121
[O-]C(=O)C1CN(CCO1)C(=O)c1ccc(=O)n(Cc2ccccc2Cl)c1	Z1455208154	-1,132
[O-]C(=O)C1CN(CCO1)C(=O)c1ccc(s1)S(=O)(=O)c1ccccc1	Z1455210184	-1,133
COc1ccc(cc1)-c1cncc(c1)C(=O)N1CCOC(C1)C([O-])=O	Z1455216081	-1,142
[O-]C(=O)C1CN(CCO1)C(=O)c1cnc(Cc2ccccc2)s1	Z1455216934	-1,124

CN(CCC([O-])=O)C(=O)C1CCN(C1=O)c1cc(Cl)cc(Cl)c1	Z1455276421	-1,058
CC1(NC(=O)N(CC([O-])=O)C1=O)c1cccc(c1)C(N)=O	Z1470261493	-1,102
Cc1nc([S-])sc1CC(=O)N1CCC(C1)Oc1ccccc1Cl	Z1500765875	-1,062
CC(NC(=O)CNC(=O)c1[nH+]cccc1[O-])c1cccc(c1)N1CCOC1=O	Z1505526685	-1,069
Cn1cc(CC(=O)N2CCCC(C2)n2cc(cn2)C([O-])=O)c2ccccc12	Z1563258271	-1,133
[O-]C(=O)C1CCC(CC1)NC(=O)Cc1cn2cc(Cl)cc(Cl)c2n1	Z1570112466	-1,110
Cc1nn(Cc2ccccc2)c2sc(cc12)C(=O)NCCCCC([O-])=O	Z1583367866	-1,060
Cc1nn(Cc2cccc(Cl)c2)cc1C(=O)N1CCCCC1C([O-])=O	Z1603240994	-1,089
[O-]C(=O)CCC(=O)N1CC(C1)OCc1cccc2ccccc12	Z1603307264	-1,159
CCc1ccc(cc1)C(C)CC(=O)N1CCSCC1CC([O-])=O	Z1603396314	-1,028
COc1ccc(cc1)C12CC3CC(CC(C3)(C1)C(=O)NCC(C)C([O-])=O)C2	Z1603524636	-1,035
[O-]C(=O)CC1CN(CCO1)C(=O)CCCc1csc2ccccc12	Z1603549421	-1,057
CC(N(C)C(=O)c1ccc(o1)C([O-])=O)c1cc2ccccc2o1	Z1609472431	-1,074
CCc1ccc(cc1)-c1csc(NC(=O)C(C)OC(=O)Cn2[n-]c(=O)c3ccccc3c2=O)n1	Z16297160	-1,067
CC(C)[NH+](CC(=O)NCC([O-])=O)Cc1ccc(s1)-c1ccc(C)cc1	Z1635179015	-1,130
[O-]C(=O)CNC(=O)CN1CCN(Cc2ccccc2)C2(CCCCC2)C1	Z1635382180	-1,046
[O-]C(=O)CCC(=O)Nc1ccccc1OS(=O)(=O)C1=Cc2ccccc2CC1	Z164708502	-1,027
Cc1ccc(c(C)c1)-n1nnnc1SCC(=O)NC(Cc1c[nH]c2ccccc12)C([O-])=O	Z16587294	-1,108
CCOc1ccc(cc1)-n1c(COc2ccc(Br)cc2)nnc1SCC(=O)NC(C(C)C)C([O-])=O	Z16595339	-1,071
[O-]C(=O)c1coc(c1)C(=O)NCCCN1C(=O)COc2ccccc12	Z1675218108	-1,043
C[NH+](C)C1CCCN(C1)S(=O)(=O)c1ccc(cc1)N1CC(CC1=O)C([O-])=O	Z1682205537	-1,056
[O-]C(=O)c1cnc(SCC(=O)Nc2ccccc2-c2ccccc2)n1-c1ccc(F)cc1	Z16885365	-1,127
[O-]C(=O)C1CCCN(C1)C(=O)\C=C\c1ccnc2ccccc12	Z1696076573	-1,125
Cc1ccc(C)c(c1)S(=O)(=O)NCCN1CC(CC1=O)C([O-])=O	Z1738877858	-1,150
CC1Cc2cc(ccc2O1)S(=O)(=O)NCCN1CC(CC1=O)C([O-])=O	Z1738877938	-1,177
[O-]C(=O)c1cnn(c1)C1CCCN(C1)S(=O)(=O)c1cc2ccccc2o1	Z1739179814	-1,121
[O-]C(=O)C1CCN(CC1C([O-])=O)S(=O)(=O)c1csc(n1)-c1ccc(Cl)cc1	Z1739185884	-1,140
COc1cc(CC(=O)N2CC(O)C(C2)[NH+](C)CC([O-])=O)ccc1C	Z1762716382	-1,053
Cc1ccccc1CCNC(=O)C[NH+]1CCC(C)(C1)NCC([O-])=O	Z1762793214	-1,041
Cc1ccc2[nH]c(CNC(=O)c3ccc(c([O-])c3)[N+]([O-])=O)nc2c1	Z1834895765	-1,045
[O-]C(=O)CNC(=O)Cc1cc2c(ccc3ccccc23)o1	Z1869504135	-1,055
[O-]C(=O)c1ccc(nc1)C(=O)NCC(=O)Nc1cccc2ccccc12	Z1869590040	-1,123

CCOc1ccccc1-c1cccc(c1)C(=O)NC1(CCSC1)C([O-])=O	Z1869986520	-1,026
[O-]C(=O)c1ccc(nc1)C(=O)N(CCc1ccccc1)c1ccccc1	Z1870040797	-1,063
CC(N(C)C(=O)C1CC1C([O-])=O)c1sc(nc1C)-c1ccccc1	Z1870437846	-1,062
[O-]C(=O)COc1cccc(c1)C(=O)NC1C2CCc3ccccc3C12	Z1870489766	-1,110
CCc1ccnc(CNS(=O)(=O)c2cc(C)c(s2)C([O-])=O)c1	Z1973568377	-1,032
CC1Cc2ccc(C)cc2C1NC(=O)c1cnc(cn1)C([O-])=O	Z1991715548	-1,127
[O-]C(=O)Cn1cc(CNC(=O)c2cccc(n2)-n2cccn2)nn1	Z1991869568	-1,054
CC(C)Oc1ccc(cc1)-n1nnc(C(=O)NCc2cn(CC([O-])=O)nn2)c1C	Z1991871890	-1,144
CCc1oc(cc1C([O-])=O)C(=O)NCc1cc(OC)c(OC)cc1OC	Z2025672644	-1,049
[O-]C(=O)CC1COCCN1C(=O)c1ccc(Oc2ccccc2)o1	Z2147945371	-1,058
[O-]C(=O)C(Cc1c[nH]c2ccccc12)NC(=O)NCc1cc(on1)-c1cccs1	Z223122822	-1,043
[O-]C(=O)CCNC(=O)c1ccc(Oc2ccc(F)cc2)cc1	Z224131644	-1,030
CC(=O)N1CCc2cc(ccc12)C(=O)NCc1ccc(cc1)C([O-])=O	Z224155452	-1,081
[O-]C(=O)COCCNC(=O)COc1ccc(Cl)c2ccccc12	Z2256293796	-1,092
CC1CN(CCC1C([O-])=O)C(=O)c1cc2c(F)cccc2[nH]1	Z2256347747	-1,028
[O-]C(=O)CNC(=O)C[NH+]1CCC(CC1)c1cccc2ccccc12	Z2257007264	-1,113
CN(Cc1ccc(OCC=C)cc1)S(=O)(=O)c1ccsc1C([O-])=O	Z227872098	-1,026
[O-]C(=O)CC(NS(=O)(=O)c1ccc2OCCCOc2c1)c1cccs1	Z227932256	-1,029
COC1CC(CC([O-])=O)N(C1)C(=O)COc1ccccc1-c1ccccc1	Z2296029969	-1,130
Cc1c(cnn1C1CCN(CC1)C(=O)c1cc2ccc(Cl)cc2[nH]1)C([O-])=O	Z2296062134	-1,113
[O-]C(=O)c1cnn(c1)C1CCN(CC1)C(=O)c1ccc2n[nH]cc2c1	Z2296071895	-1,151
CCC(C([O-])=O)S(=O)Cc1csc(n1)-c1ccc2OCOc2c1	Z2301748685	-1,036
[O-]C(=O)C1CCN(C1)C(=O)NC1CCOC(C1)c1ccc(F)cc1	Z2353355224	-1,052
[O-]C(=O)CCNC(=O)N1CCC(C1)n1ncc2ccccc12	Z2353392374	-1,081
CC(C([O-])=O)C(=O)NC1CCCN(C1=O)c1ccccc1F	Z2361456651	-1,111
CC(C([O-])=O)C(=O)NC1CN(Cc2ccccc2C(F)(F)F)C(=O)C1	Z2361458637	-1,068
CC(C)c1noc(Cc2ccc(NC(=O)NCC(C)(O)C([O-])=O)cc2)n1	Z2362317423	-1,032
[O-]C(=O)c1cnc(cn1)C(=O)NCc1cccc2NCCCc12	Z2362409054	-1,120
[O-]C(=O)CS(=O)(=O)CC[NH+]1CCCC1c1cccc2ccccc12	Z2368931792	-1,127
[O-]C(=O)CS(=O)(=O)CC[NH+]1CCC(CC1)c1cccc2ccccc12	Z2368931867	-1,120
[O-]C(=O)CS(=O)(=O)CC[NH+]1CCCC2(CCCc3ccccc23)C1	Z2368933227	-1,121
[O-]C(=O)C1CCCN(C1)C(=O)C(=O)NCc1cc(Cl)c2OCCOc2c1	Z2370369361	-1,158

CC1CC(CN(C1)C(=O)Nc1ccnc(c1)-c1ccccc1)C([O-])=O	Z2376768926	-1,114
CC(NS(=O)(=O)c1ccccc1F)C(=O)N1CC(CCC1C)C([O-])=O	Z2376909550	-1,031
[O-]C(=O)CN1CCCCC(N2CCN(Cc3cccc4ccccc34)CC2)C1=O	Z2387260303	-1,125
CC([NH+]1CC[NH+](CC1)C1CCCCN(CC([O-])=O)C1=O)c1ccccc1Cl	Z2387260921	-1,135
COc1cccc(c1)C(C)N1CCN(CC1)C1CCCCN(CC([O-])=O)C1=O	Z2387262241	-1,078
CC1CN(CC[NH+]1CC(O)c1ccccc1)C1CCCCN(CC([O-])=O)C1=O	Z2387262537	-1,141
[O-]C(=O)CN1CCCCC([NH2+]C2CCOc3c(Br)cccc23)C1=O	Z2387267271	-1,124
[O-]C(=O)c1cc(c[nH]1)S(=O)(=O)NC1CCCOc2cc(F)ccc12	Z2437626057	-1,110
CC(Oc1ccccc1C)C(=O)N1CCN(CC1)C(=O)c1cnc(cn1)C([O-])=O	Z2442718712	-1,044
CCOc1ccc(Nc2nc(NCC([O-])=O)nc(N)c2[N+]([O-])=O)cc1	Z244561718	-1,056
[O-]C(=O)Cn1cc(NC(=O)Cc2ccc3CCCc3c2)cn1	Z2446566342	-1,044
CC(C)c1nn(C)c2sc(cc12)C(=O)Nc1ncn(CC([O-])=O)n1	Z2446731507	-1,055
CC(N1C(=O)c2ccc(C)cc2C1=O)C(=O)Nc1[nH+]cccc1[O-]	Z244928884	-1,129
[O-]C(=O)c1cn(nn1)C1CCCN(C1)C(=O)c1cc2cc(F)ccc2[nH]1	Z2460789070	-1,114
Cn1c2ccccc2n(CC(=O)N2CCCC(C2)n2cc(nn2)C([O-])=O)c1=O	Z2460789784	-1,119
C[NH+]1CCCC(CNc2ccc(nn2)C([O-])=O)C1c1ccc(cc1)C(F)(F)F	Z2465950862	-1,027
CCC1CN(CC[NH+]1Cc1ccccc1)c1nc(co1)C([O-])=O	Z2465975086	-1,026
[O-]C(=O)CCC(=O)N1N=C(CC1c1ccc2nccnc2c1)c1cccc(Cl)c1	Z2472759354	-1,033
COc1ccc(cc1OC)C1CCC[NH+]1Cc1cc(co1)C([O-])=O	Z2482868899	-1,040
COc1ccc2C[NH+](CCc3cn(CC([O-])=O)nn3)CCCc2c1	Z2482971026	-1,131
CCn1c(C[NH+]2CCC(C)(C2)c2ccccc2)nc2cc(ccc12)C([O-])=O	Z2483045010	-1,102
[O-]C(=O)c1cnn(c1)C1CCC[NH+](Cc2cc3ccccc3o2)C1	Z2483398500	-1,117
Cc1cccn2cc(CC(=O)NC3CC(C([O-])=O)c4ccccc34)[nH+]c12	Z2489741334	-1,029
CC(CNC(=O)N1CCn2c(C1)cc1ccc(Cl)cc21)C([O-])=O	Z2504967592	-1,048
[O-]C(=O)CC1(CCOCC1)C(=O)Nc1ccc2nc(oc2c1)C1CC1	Z2569847759	-1,050
[O-]C(=O)C1CN(C(=O)C1)c1cccc(c1)-c1ccc2OCCc2c1	Z2581527543	-1,232
CCCc1c(nnn1Cc1cccc(c1)-c1ccc(C)o1)C([O-])=O	Z2581554777	-1,030
Cn1ccc2c(cccc12)C(=O)N1CCC(CC1)n1cc(nn1)C([O-])=O	Z2595724277	-1,144
[O-]C(=O)c1cn(nn1)C1CCN(CC1)C(=O)c1ccc2CCCc2c1	Z2595724619	-1,158
Cc1c(Cl)cccc1C(=O)N1CCC(CC1)n1cc(nn1)C([O-])=O	Z2595725364	-1,112
Cc1c([nH]c(=O)c2ccccc12)C(=O)N1CCC(CC1)n1cc(nn1)C([O-])=O	Z2595725563	-1,128
COc1ccc(cc1)-c1cncc(c1)C(=O)NC1CC(C1)C([O-])=O	Z2595761057	-1,090

COc1ccc2nc(C)cc(SCC(NC(=O)\C=C\C([O-])=O)C([O-])=O)c2c1	Z259632350	-1,151
[O-]C(=O)CN1CCC([NH+]2CCC(CC2)c2nc(n[nH]2)-c2ccccc2)C1=O	Z2605220809	-1,086
[O-]C(=O)CN1CCC([NH2+]C(C2CCCc3ccccc23)c2ccccc2)C1=O	Z2605244725	-1,111
[O-]C(=O)CN1CCC(C1=O)n1nnc2c(Cl)cccc2c1=O	Z2609801542	-1,114
CS(=O)(=O)N1CCN(Cc2ccccc12)C(=O)C1CC(C1)C([O-])=O	Z2627894991	-1,029
[O-]C(=O)c1cnc(CNS(=O)(=O)c2cccc3CCCCc23)nc1	Z2721412938	-1,110
[O-]C(=O)c1cnc(C[NH+]2CCCC(C2)Oc2ccc(Cl)c(Cl)c2)cn1	Z2721842469	-1,060
CC1CC(C[NH+]1CCS(=O)(=O)CC([O-])=O)c1ccc(Br)cc1	Z2722367051	-1,041
[O-]C(=O)C1CC1C(=O)[N-]S(=O)(=O)CCc1ccc2OCCc2c1	Z2735463914	-1,113
[O-]C(=O)C1CC1C(=O)[N-]S(=O)(=O)CCC1CCc2ccccc12	Z2735473429	-1,028
[O-]C(=O)c1cn(nn1)C1CCCN(C1)C(=O)C1C2Cc3ccccc3C12	Z2755437230	-1,146
[O-]C(=O)c1cnc(s1)C1CCN(CC1)C(=O)C1Cc2ccccc2O1	Z2755503002	-1,089
COc1ccc2CCN(CCc2c1)C(=O)C1CC(C1)C([O-])=O	Z2755554601	-1,115
[O-]C(=O)c1nnn(C2CCN(CC2)C(=O)c2[nH]nc3ccccc23)c1C(F)F	Z2755627735	-1,067
[O-]C(=O)C1CCC([NH2+]1)C(=O)NCc1cccc(COc2ccccc2)c1	Z2764689987	-1,028
[O-]C(=O)c1ccc(NC(NC(=O)c2ccco2)C(=O)c2ccccc2)cc1	Z276544702	-1,038
CCn1c(C[NH+]2CCC3(CCc4ccccc34)C2)nc2cc(ccc12)C([O-])=O	Z2790718081	-1,120
CC(OC(C)C(=O)N1CCCC2(CCCc3ccccc23)C1)C([O-])=O	Z2793548331	-1,097
[O-]C(=O)c1ccc(cn1)C(=O)N1CCCC1c1ccc2OCCOc2c1	Z284101850	-1,033
[O-]C(=O)c1cc(C[NH+]2CC(C2)OCc2cccc3ccccc23)[nH]n1	Z2898077061	-1,142
Cc1nc2ccc(cc2s1)C(=O)N1CCC(CC1)n1cc(cn1)C([O-])=O	Z2911081744	-1,149
[O-]C(=O)c1cnn(c1)C1CCN(CC1)C(=O)c1cc(F)ccc1-n1cccn1	Z2911082529	-1,106
OC(Cc1cccc(c1)C(F)(F)F)C(=O)N1CCC(C2CCCC12)C([O-])=O	Z2911481194	-1,035
CC(C)CC(CC([O-])=O)C(=O)N1CC2CC(C2C1)c1ccccc1	Z2982912722	-1,032
[O-]C(=O)c1cncc(C[NH+]2CCC(CC2)c2c[nH]c3ccccc23)c1	Z2996604390	-1,108
[O-]C(=O)c1cc(c[nH]1)S(=O)(=O)NC1CCCc2ccccc2C1	Z3008864535	-1,174
CC(NC(=O)C[NH2+]C(C)(C)CC([O-])=O)c1ccc(Cl)c(Cl)c1	Z3016340668	-1,035
[O-]C(=O)C1CCC1C(=O)NC1CCN(CC1)C(=O)c1ccccc1F	Z3017785809	-1,064
[O-]C(=O)C1CN(C2CC[NH+](Cc3csc4ccccc34)C2)C(=O)C1	Z3061111004	-1,158
[O-]C(=O)C1=NOC2(C1)CCN(CC2)C(=O)c1cccc2cc[nH]c12	Z3216017609	-1,056
[O-]C(=O)C1CC=CCC1C(=O)Nc1ccccc1C(=O)Nc1ccc(Cl)c(Cl)c1	Z3279254512	-1,062
CC(C)c1cc(nc(n1)C(C)C)N1CCC(C1)n1cc(nn1)C([O-])=O	Z3290813540	-1,035

CCCc1cccc(C[NH2+]C2CCOC3(CC(C3)C([O-])=O)C2)c1	Z3301189034	-1,046
CC1=NC(CCC([O-])=O)C(=O)Nc2cc3OCOc3cc12	Z3307796746	-1,058
[O-]c1ccc[nH+]c1C(=O)NC1CN(C1=O)c1ccccc1	Z3488516360	-1,131
CC1(C)CCN(CC1c1ccccc1)C(=O)c1cc([nH]n1)C([O-])=O	Z3523961029	-1,125
COc1cc(C)c(cc1OC)S(=O)(=O)Nc1ccc(cc1)C([O-])=O	Z353959104	-1,066
[O-]C(=O)c1coc(Cn2cnc3scc(-c4ccccc4)c3c2=O)c1	Z3668860174	-1,102
Cc1cc(SCC(NC(=O)c2ccccc2)C([O-])=O)c2ccccc2n1	Z367650394	-1,029
Cc1cc(SCC(NC(=O)CCC([O-])=O)C([O-])=O)c2ccccc2n1	Z367678788	-1,027
Cc1cc(C)c(C)c(c1C)S(=O)(=O)Nc1ccc(cc1)C(=O)NCC([O-])=O	Z45581694	-1,109
CCOC(=O)C1=C(C)N(C(=O)NC1c1ccc(Cl)cc1)c1ccc(cc1)C([O-])=O	Z46978002	-1,068
[O-]C(=O)CCC(=O)N1CCN(CC1)S(=O)(=O)c1ccc2ccccc2c1	Z55148706	-1,134
CCOC(=O)c1sc2nc(CS(=O)(=O)CC([O-])=O)[nH]c(=O)c2c1C	Z55928965	-1,052
Cn1c(=O)c2C(CC([O-])=O)C(=O)Nc2n(Cc2ccccc2)c1=O	Z56691575	-1,084
[O-]C(=O)c1cccc(c1)N1C(=O)C2C(C3c4ccccc4C2c2ccccc32)C1=O	Z56759649	-1,052
COc1ccccc1Oc1ccc(cc1)S(=O)(=O)NC(CCSC)C([O-])=O	Z56883367	-1,049
[O-<br>]S(=O)(=O)CCN1C(=S)S\C(C1=O)=C1\C(=O)N(CC(=O)Nc2cccc(Br)c2)c2ccccc12	Z56958935	-1,117
CCOC(=O)c1ccc(NC2SC(=O)N(CCC([O-])=O)C2=O)cc1	Z57179878	-1,061
[O-<br>]C(=O)CCN1C(=O)C2C3CC(C4C3Sc3[nH]c(=O)sc3C4c3cccc(Oc4ccccc4)c3)C2C1=O	Z57707042	-1,241
COc1cc(cc(OC)c1O)C1C2C3CC(C2Sc2[nH]c(=O)sc12)C1C3C(=O)N(C(C)C([O-<br>])=O)C1=O	Z57707052	-1,132
OC1=C(C(N(CCC([O-])=O)C1=O)c1ccc(Cl)cc1)C(=O)c1ccc(Cl)cc1	Z57790704	-1,164
[O-]C(=O)C1CN(C(=O)C1)c1cccc(c1)N1CCCCC1=O	Z729606496	-1,116
CN(C)C(=O)Cc1ccc(NS(=O)(=O)c2ccc(cc2)N2CC(CC2=O)C([O-])=O)cc1	Z738201678	-1,141
[O-]C(=O)COc1ccc(Cc2nc3ccsc3c(=O)[nH]2)cc1	Z840271324	-1,034
COc1cc(cc(Cl)c1OC(C)C)C(=O)N1CCC(CC1)C([O-])=O	Z85886589	-1,068
[O-]C(=O)CC(NC(=O)c1ccc(o1)-c1ccc(F)cc1)c1ccc(F)cc1	Z85905794	-1,079
CC(CC([O-])=O)NC(=O)CCSc1ccccc1Cl	Z85971880	-1,036
CN(CC(=O)NC(CC([O-])=O)c1ccccc1)S(=O)(=O)c1ccc2ccccc2c1	Z98661094	-1,071
CC(C)(C)c1ccc(NS(=O)(=O)c2c[nH]c(c2)C([O-])=O)cc1	Z994089832	-1,151
CC1CCN(CC1)c1ccc(NS(=O)(=O)c2c[nH]c(c2)C([O-])=O)cc1	Z997763778	-1,185
[O-]C(=O)c1cc(c[nH]1)S(=O)(=O)N1CCc2ccc(Cl)cc12	Z997820334	-1,084

4.3 Ultrafast docking virtual hits*[1]*

Top 179 compounds were selected for further in vitro studies:

Table S6. Virtual hits obtained using Ultrafast docking method.

Smiles	ID	Docking Score
CN(C(=O)c1cn2ccccc2n1)C(C)(C)C([O-])=O	Z1021331764	-2,999
CC(C)(CC(=O)Nc1ccc(Cl)cc1C(N)=O)C([O-])=O	Z1213003361	-3,208
Oc1cccc(c1)-c1cc(no1)C([O-])=O	Z1245633796	-2,851
CC(C)(CC(=O)N1CCc2sccc2C1)C([O-])=O	Z1259093131	-3,262
C[C@]1(CC(=NO1)c1ccccc1Cl)C([O-])=O	Z1263738677	-2,741
Cc1c(Br)cccc1C(=O)N[C@](C)(C1CC1)C([O-])=O	Z1263878816	-2,951
Cc1cc(C)n2nc(nc2n1)C(=O)NC[C@@](C)(O)C([O-])=O	Z1270411711	-3,980
O[C@H](CNC(=O)[C@@H]1CC[C@H]2CCCC[C@@H]2[NH2+]1)C([O-])=O	Z1276741384	-3,322
CC[C@@](C)(NC(=O)c1ccc(CS(C)(=O)=O)cc1)C([O-])=O	Z1344172889	-2,994
CN(CC([O-])=O)C(=O)c1ccc2NC(=O)CCc2c1	Z1443721537	-3,681
[O-]C(=O)CCNC(=O)c1nn(cc1Br)-c1ccc(F)cc1	Z1443847037	-3,079
CC[C@H](C)[C@H](NC(=O)c1cnn2cccnc12)C([O-])=O	Z1444323471	-2,902
CC[C@@](NC(=O)[C@@H]1Cc2cc(F)ccc2O1)(C([O-])=O)c1ccccc1	Z1444494573	-3,507
[O-]C(=O)[C@@H](NC(=O)c1ccc(cc1)N1CCNC1=O)C1CC1	Z1444708093	-3,213
[O-]C(=O)CCNC(=O)c1ccc(NC(=O)c2ccc3cc[nH]c3c2)cc1	Z1444897161	-3,358
C[C@H](CNC(=O)c1ccc(CSc2ccccc2)o1)C([O-])=O	Z1445216557	-3,149
CCCc1cc([nH]n1)C(=O)NC[C@H](C)C([O-])=O	Z1445219905	-3,053
C[C@H](NC(=O)c1ccn(n1)-c1cccc(F)c1)C([O-])=O	Z1445244335	-3,366
CC(C)C[C@@H](CNC(=O)Cc1c(C)[nH]c2ccnn2c1=O)C([O-])=O	Z1445522149	-3,265
CN1CC[C@H](CC1=O)C(=O)N1CC[C@](C)(C1)C([O-])=O	Z1445589320	-2,762
Cc1ccc(nc1)N1CCC(CC1)C(=O)NCC([O-])=O	Z1455011032	-3,249
C[C@H]1[C@@H](Oc2ccccc12)C(=O)N(C)CCC([O-])=O	Z1455275593	-2,756
Cc1nn(c(C)c1C(=O)N1CC[C@H](C1)C([O-])=O)-c1ccc(F)cc1F	Z1528734101	-3,004
[O-]C(=O)[C@H](CNC(=O)[C@@H]1CC11CCOCC1)Cc1ccc2ccccc2c1	Z1603546790	-3,554
C[C@](NC(=O)c1ccc(OCC2CC2)cc1)(C1CC1)C([O-])=O	Z1603584482	-3,407

Cc1nn(Cc2ccc(C)cc2)c(C)c1CCC(=O)NC1(CC1)C([O-])=O	Z1603607484	-3,341
[O-]C(=O)C1(CC1)NC(=O)[C@@H]1SCCc2ccccc12	Z1603607950	-2,965
[O-]C(=O)C1(CCC1)NC(=O)c1[nH]c2ccc(Cl)cc2c1Cl	Z1603621279	-3,368
COc1cc(cc2OCCOc12)C(=O)N[C@H](Cc1ccc(F)cc1)C([O-])=O	Z1603655942	-3,265
[O-]C(=O)[C@@H](Cc1c[nH]c2cc(F)ccc12)NC(=O)c1cc2ccccc2[nH]1	Z1603708927	-3,773
COc1ccccc1C[C@H](CNC(=O)c1cn2ccc(C)cc2n1)C([O-])=O	Z1603719561	-3,470
CCC(CC)(CNC(=O)[C@H](CCSC)NC(=O)c1cccc(C)c1)C([O-])=O	Z1603720859	-3,423
CCC[C@H](NC(=O)CCc1ccc(Cl)s1)C([O-])=O	Z1609602981	-2,652
[O-]C(=O)CCCC(=O)Nc1cccc(c1)S(=O)(=O)Nc1ccc(SC(F)F)cc1	Z169880312	-3,145
C[C@@](O)(CNC(=O)[C@@H]1C[C@H]1c1ccc2OCCOc2c1)C([O-])=O	Z1715385555	-3,141
[O-]C(=O)[C@@H]1C[C@H]1C(=O)N1CCN(CC1)c1ncc(Cl)cc1Cl	Z1870383382	-3,399
[O-]C(=O)[C@@H]1C[C@H]1C(=O)Nc1ccc(cc1)-n1ncc2ccccc12	Z1870457637	-3,065
Cc1oc2nc[nH]c(=O)c2c1C(=O)N1C[C@@H]2CCC[C@]2(C1)C([O-])=O	Z1983855637	-2,756
CCc1ccccc1NC(=O)CN(C)C(=O)c1cnc(cn1)C([O-])=O	Z1991665237	-3,552
Cn1cccc1C(=O)NC[C@@](C)(C([O-])=O)c1ccccc1	Z1991894277	-3,418
Cc1cccc(C(=O)NC[C@@](C)(C([O-])=O)c2ccccc2)c1Cl	Z1991896568	-3,315
[O-]C(=O)CCC(=O)c1cccc(NS(=O)(=O)CCc2cccc(Cl)c2)c1	Z2067509084	-3,660
CCC(CC)(CC(=O)N1CC[C@H]2CCC[C@@H]12)C([O-])=O	Z2227405654	-3,118
COc1ccc(N2C[C@H](CC2=O)C(=O)N[C@@H](C)C([O-])=O)c(OC)c1	Z224133452	-2,921
Cc1c(oc2c(C)ccc(C)c12)C(=O)NCC1(CC1)C([O-])=O	Z2256307546	-3,108
CC(C)(CCNC(=O)CCCc1ccc2NC(=O)Cc2c1)C([O-])=O	Z2295949404	-3,570
[O-]C(=O)[C@@H]1C[C@@H](C1)NC(=O)c1cccc(c1)-c1ccoc1	Z2352635129	-3,213
[O-]C(=O)Cn1nc2CCCCCn2c1=O	Z235352019	-4,128
CC(C)c1noc(N[C@H](Cc2ccccc2)C([O-])=O)n1	Z2358579444	-2,829
COC[C@]1(CCCN1C(=O)COc1c(C)cccc1C)C([O-])=O	Z2386657737	-2,821
COC[C@]1(CCCN1C(=O)CCc1ccc(C)cc1C)C([O-])=O	Z2386662429	-2,798
[O-]C(=O)c1cn(nn1)[C@@H]1CCN(C1)c1nc(cs1)-c1ccc(F)cc1	Z2396011452	-3,873
Cn1nccc1[C@H](NC(=O)CC[C@@H]1CCOC1)C([O-])=O	Z2432766892	-3,010
[O-]C(=O)CCCS(=O)(=O)N1CCO[C@]2(CCc3ccccc23)C1	Z2437511386	-3,442
[O-]C(=O)CCC(=O)Nc1nc(C[NH+]2CCCCCC2)cs1	Z2442162418	-2,873

CN([C@H]1C[C@@H]2CC[C@H](C1)N2C(=O)CCC([O-])=O)C(=O)c1ccccc1	Z2442934503	-3,735
CC[C@H](NC(=O)CN1CCCCC1=O)C([O-])=O	Z2443698190	-3,589
COc1cc(OC)cc(c1)N1CC[C@@H](N(C)C(=O)[C@@H]2C[C@H]2C([O-])=O)C1=O	Z2466933858	-3,780
CC(C)c1ccc(C[C@H]2CC[N@@H+](Cc3nc(co3)C([O-])=O)CC2)cc1	Z2483066943	-3,248
C[C@H](CC([O-])=O)CC(=O)N1CCN(CC1)C(=O)Cc1c[nH]c2ccccc12	Z2506955864	-3,755
CC(C)(C)[C@H](NC(=O)c1ccc(nc1)C(F)F)C([O-])=O	Z2506960890	-3,008
[O-]C(=O)[C@@H](CC1CC1)NC(=O)c1cc2cscc2s1	Z2582403177	-3,399
[O-]C(=O)[C@@H]1OCC[C@@H]1NC(=O)c1cc(F)cc2cccnc12	Z2595664351	-3,097
[O-]C(=O)C1(CNC(=O)C2CCN(CC2)C(=O)c2ccccc2)CC1	Z2656172499	-3,068
[O-]C(=O)[C@H](CNC(=O)[C@@H]1[C@H]2Cc3ccccc3[C@@H]12)Cc1ccc2ccccc2c1	Z2755341268	-3,618
CO[C@H]1C[C@@H](C1)C(=O)NC[C@@H](C1CCOCC1)C([O-])=O	Z2755423684	-3,065
[O-]C(=O)[C@H](CNC(=O)[C@@H]1[C@H]2Cc3c(cccc3Cl)[C@@H]12)CC1CC1	Z2755441864	-3,605
CCN1CCOC[C@@H]1C(=O)N(C)C[C@H](C)C([O-])=O	Z2830729856	-3,035
C[C@H](Cc1cnc[nH]1)C(=O)N1CC[C@](C)(C1)C([O-])=O	Z2830976022	-2,704
[O-]C(=O)[C@H](CNC(=O)[C@@H]1CCc2cccnc12)Cc1ccccc1	Z2831426920	-3,538
C[C@@](NC(=O)c1cnc2ccccn12)(C1CC1)C([O-])=O	Z2832171806	-3,297
[O-]C(=O)C1(CCCC1)NC(=O)c1cnc2CCCCn12	Z2848079408	-3,289
C[C@H](NC(=O)Nc1ccc(C)cc1C)C([O-])=O	Z2858936381	-2,957
CN(CC([O-])=O)C(=O)NCC(C)(C)Cc1cccc(Cl)c1	Z2865280818	-3,542
[O-]C(=O)CN(Cc1ccccc1)C(=O)NCc1cc[nH]n1	Z2865543120	-2,695
C[C@@H]1[C@@H](CCN1C(=O)c1ccc(cc1)-n1ncc(C([O-])=O)c1C)[N@H+](C)CCO	Z2865626449	-3,659
CC(C)C[C@H]([NH3+])C(=O)N[C@@H](C(C)C)C([O-])=O	Z2893987095	-3,179
C[C@@H](N(C)C(=O)c1cn2CC[C@H](C)Cc2n1)C([O-])=O	Z2895942023	-3,173
C[C@H](NC(=O)c1ccc(NCc2nc3CCCc3s2)cc1)C([O-])=O	Z2897365512	-3,774
C[C@H](NC(=O)c1ccc(NCc2cccc3CCCc23)cc1)C([O-])=O	Z2897365646	-3,840
[O-]C(=O)C1(CCOCC1)NC(=O)c1ncc2CCCCn12	Z2897767238	-3,175
Cc1nc(cs1)-c1ccc(s1)C(=O)NC(C)(C)C([O-])=O	Z2897883459	-3,005
[O-]C(=O)[C@@H](NC(=O)NCc1cccc(Cn2cccn2)c1)C1CC1	Z2901815559	-3,260

[O-]C(=O)CCC(=O)N[C@@H]1CCc2ccccc2C1	Z2902156563	-3,347
Oc1ccc(C[C@@H](CNC(=O)Cc2ccc(cc2)C(F)(F)F)C([O-])=O)cc1	Z2912551883	-3,813
CC[C@@H](NC(=O)NC[C@H](c1ccc(Cl)cc1)n1cccn1)C([O-])=O	Z2920443580	-2,946
[O-]C(=O)[C@H]1[C@H]2CCC[C@H]2CN1C(=O)NCc1ccoc1	Z2936788393	-3,636
[O-]C(=O)[C@@H]1CCN1C(=O)N1CCc2ccccc12	Z2936916063	-3,534
O[C@H](CNC(=O)NC[C@H]1C[N@H+]2CCC[C@@H]2CO1)C([O-])=O	Z2940234196	-3,189
Cc1ccc(Cl)cc1-c1ccc(o1)C(=O)NCC([O-])=O	Z2991038604	-3,400
[O-]C(=O)CCC(=O)Nc1ccncc1F	Z3015233556	-3,422
C[C@@](Cn1cnc2ccccc12)(NC(=O)CC1CCOCC1)C([O-])=O	Z3016339487	-2,816
Cn1cc(cn1)N1CC[C@@H](C1)NC(=O)CC1(CCCC1)C([O-])=O	Z3024673744	-3,020
CC1(C)[C@H]([C@H]1C(=O)N[C@@H]1CCCn2nccc12)C([O-])=O	Z3036248534	-3,548
[O-]C(=O)C1(CC(=O)NCc2cccc3cccnc23)CCC1	Z3037294184	-3,060
Cc1ccc(cc1NC(=O)CC1(CCC1)C([O-])=O)C(N)=O	Z3050382907	-3,447
[O-]C(=O)[C@@H]1CCCN1C(=O)NCc1cccc2COCCc12	Z3068298804	-3,559
C[C@H]1CCC[C@@H](C1)OCc1cccc(NC(=O)CCC([O-])=O)c1	Z3069705677	-3,547
CC(C)[C@@](C)(CC(=O)Nc1ncccn1)C([O-])=O	Z3070229897	-3,356
CN1CCc2ccc(NC(=O)CCC([O-])=O)cc12	Z3070230000	-3,189
COc1ccc(Br)cc1CNC(=O)CC(C)(C)C([O-])=O	Z3072410321	-3,161
[O-]C(=O)[C@H]1C[C@H](C1)C(=O)NCc1cccc(Oc2ccc(F)cc2)n1	Z3173248890	-3,297
[O-]C(=O)[C@@H]1OCC[C@@H]1CNC(=O)[C@@H]1[C@H]2CCCO[C@@H]12	Z3173313929	-3,264
Cc1nn(C)cc1C[C@H](NC(=O)c1cc(Cl)cn1C)C([O-])=O	Z3290660127	-3,339
CC(C)(CNC(=O)[C@@H]1CC[C@H]1C([O-])=O)C1CC[NH2+]CC1	Z3294127862	-3,514
Cn1ccc2c(cccc12)C(=O)N1CC[C@](CF)(C1)C([O-])=O	Z3300345687	-3,308
CC(C)[NH+]1CCN(CC1)c1cc(C)cc(NC(=O)CCC([O-])=O)c1	Z3300393948	-3,567
CN(CC([O-])=O)C(=O)NC[C@@H]1CSc2ccccc2O1	Z3301447925	-3,082
Cc1cccc2c(CCC(=O)NCC3(CC([O-])=O)CC3)c[nH]c12	Z3390931925	-3,557
C[C@H](NC(=O)[C@@H]1C[C@@H](C1)C([O-])=O)c1ccc2CCCCc2c1	Z3466179106	-2,643
CC(=O)NCc1ccc(CNC(=O)[C@@H]2CCCC[C@@H]2C([O-])=O)cc1	Z3478940364	-3,663
[O-]C(=O)COC(=O)c1ccc(cc1F)-c1ccc(Cl)cc1	Z3485328180	-3,681
CCN1CCN(CC1)C(=O)c1ccc(NC(=O)[C@@H]2C[C@@H]2C([O-])=O)cc1	Z3485757482	-3,025
[O-]C(=O)[C@@H]1C[C@@H]1C(=O)NCc1ccc(CN2CCNC2=O)cc1	Z3569351211	-3,289

Cc1cc(CCNC(=O)[C@@H]2C[C@@]2(C([O-])=O)c2ccccc2)nn1C	Z3569351420	-3,095
Cc1ccc(cc1)[C@H](CC(=O)N[C@H]1CCc2ccccc12)C([O-])=O	Z3570825707	-3,631
O[C@@H]1C[C@H](NC(=O)[C@@H]2C[C@@]2(C([O-])=O)c2ccccc2)C11CCOCC1	Z3681207846	-2,418
CC(C)[C@@]1(C[C@H]1C(=O)NCc1ccc(cc1)-n1ccnc1)C([O-])=O	Z3681207865	-3,453
CC(C)[C@]1(C[C@@H]1C(=O)Nc1cccc2nccnc12)C([O-])=O	Z3681207876	-3,353
Cc1ccc(cc1NC(=O)[C@@H]1C[C@@H]1C([O-])=O)C(=O)NCC1CC1	Z3681208436	-3,295
[O-]C(=O)[C@@H]1C[C@@H]1C(=O)Nc1ccc(F)c(c1)N1CCCNC1=O	Z3681208990	-3,754
C[C@@H](NC(=O)[C@@H]1C[C@@H]1C([O-])=O)c1ccc(c(F)c1)-n1cccn1	Z3681209262	-3,376
CC(C)(CNC(=O)[C@@H]1C[C@@H]1C([O-])=O)[C@H]1COCC11OCCO1	Z3681209269	-2,746
CC[C@H](NC(=O)[C@@H]1C[C@@H]1C([O-])=O)c1csc(C)c1	Z3681209542	-2,696
COCC(=O)N1CCC(CC1)NC(=O)[C@@H]1C[C@]1(C(C)C)C([O-])=O	Z3681369109	-2,508
Cc1cc(Cl)c(NC(=O)[C@@H]2C[C@@H]2C([O-])=O)cc1Br	Z3681420099	-3,152
[O-]C(=O)[C@@H]1C[C@@H]1C(=O)N[C@@H]1C[C@H]1c1ccc(Br)s1	Z3681624095	-3,053
CC(C)[C@@]1(C[C@H]1C(=O)NCCc1ccc2ncccc2c1)C([O-])=O	Z3681646738	-2,967
[O-]C(=O)CCC(=O)Nc1cc(c[nH]c1=O)C(F)(F)F	Z3681678989	-3,292
CC(C)[C@]1(C[C@@H]1C(=O)N[C@H]1CCCN(C1=O)c1cccnc1)C([O-])=O	Z3681737843	-3,421
C[C@@H](N1CCC[C@H](NC(=O)[C@@H]2C[C@@H]2C([O-])=O)C1=O)c1ccc(F)cc1	Z3681739124	-2,865
C[C@H](CNC(=O)CCC([O-])=O)c1ccccc1	Z3681784249	-3,294
CC(C)[C@@]1(C[C@H]1C(=O)NC[C@H]1CCOC1)C([O-])=O	Z3681799936	-2,290
[O-]C(=O)[C@H]1CC[C@H](O1)C(=O)N[C@H]1CCN(C1=O)c1cccc(OC(F)(F)F)c1	Z3698776198	-2,660
CSC[C@@](C)(C([O-])=O)n1nnc(n1)-c1ccc(C[NH+](C)C)cc1	Z3779458419	-3,394
C[C@H](NC(=O)N[C@@H](Cc1c[nH]c2ccccc12)C([O-])=O)c1ccc(cc1)-c1ccncc1	Z3969355209	-3,831
CCC(CC)(CC(=O)N[C@@H](C(C)C)c1nc2ccccc2[nH]1)C([O-])=O	Z4100907640	-3,617
[O-]C(=O)[C@H](CC(=O)Nc1ccc(F)cc1F)c1ccccc1	Z4119544425	-3,410
[O-]C(=O)C1(CC(=O)N2CCCC[C@H]2Cc2cc(F)ccc2F)CC1	Z4144014302	-3,110
[O-]C(=O)C1(CC(=O)N[C@@H]2CCc3[nH+]c(cn3C2)-c2ccccc2)CC1	Z4144020480	-3,363

CCc1c(NC(=O)CC2(CCCC2)C([O-])=O)c(=O)n(-c2ccccc2)n1C	Z4188337437	-3,382
CC(C)(C)OC(=O)c1ccc(CNC(=O)C[C@]2(CCOC2)C([O-])=O)cc1	Z4221819896	-3,033
[O-]C(=O)[C@H](Cc1c[nH]c2cccc(F)c12)NC(=O)Nc1ccccc1C(F)(F)F	Z4221819990	-3,237
CC(C)(O)C[C@H](NC(=O)c1ccc(cc1)C1CCC1)C([O-])=O	Z4280355188	-3,068
[O-]C(=O)CCCC(=O)NC12C[C@H]3C[C@H](C[C@H](C3)C1)C2	Z4338758121	-3,248
C[C@H](NC(=O)N[C@@H]([C@H]1CCc2ccccc12)C([O-])=O)c1ccc(Oc2cccnc2)cc1	Z4361984504	-3,360
[O-]C(=O)CCc1cn(Cc2nn3CCCc3c2C(=O)N2Cc3cc(F)c(F)cc3C2)nn1	Z4408411122	-3,032
[O-]C(=O)[C@H]1CC[C@H]1C(=O)NCc1ccc(cc1)-c1ccc2OCCc2c1	Z4411488282	-3,986
C[C@H](NC(=O)CCC([O-])=O)c1ccc(Cl)cc1	Z4500993554	-2,873
[O-]C(=O)CCC(=O)NCc1cc(F)ccc1F	Z4500993584	-3,019
Cc1ccn(C[C@@](C)(O)C(=O)NC[C@H](O)CC([O-])=O)c(=O)c1	Z4512917358	-3,525
CC1(C)[C@@H]([C@@H]1C(=O)N[C@@H]1[C@H]2CCO[C@H]2C1(C)C)C([O-])=O	Z4527609212	-3,393
C[C@@H](NC(=O)[C@@H]1C[C@@H]1C([O-])=O)c1nc(C)cs1	Z4534051820	-3,154
C[C@H]([C@@H](C)C(=O)NC[C@@H]1CCCCO1)C([O-])=O	Z4535746141	-3,260
[O-]C(=O)CCC(=O)N[C@H]1CCN(C1=O)c1ccccc1F	Z4549268235	-3,446
O[C@H]1C[C@](C1)(NC(=O)[C@@H]1C[C@H]1c1c(F)cccc1F)C([O-])=O	Z4549434347	-3,142
[O-]C(=O)[C@@H]1CC[C@H](O1)C(=O)NC[C@@H]1CCC2(CCCCC2)O1	Z4552302162	-3,565
Cc1ccc(cc1)C(=O)N[C@@]1(C[C@H](O)C1)C([O-])=O	Z4556068197	-3,346
Cc1ccoc1CC(=O)N[C@@]1(C[C@H](O)C1)C([O-])=O	Z4561748167	-3,299
C[C@H](NC(=O)[C@@H]1C[C@@]1(C([O-])=O)c1ccccc1)C1=NO[C@@H]2COC[C@H]12	Z4572950460	-3,547
OC[C@H](CC([O-])=O)C(=O)Nc1ncc(Cc2cccc(c2)C(F)(F)F)s1	Z4600799655	-3,668
CSCC[C@H](NC(=O)NCc1ccc(COC(C)C)cc1)C([O-])=O	Z4602008618	-2,900
OCC[C@H](NC(=O)[C@H](O)[C@@H](O)c1nc2ccccc2s1)C([O-])=O	Z4605014810	-3,441
N[C@@H](Cc1cnc[nH]1)C(=O)N[C@@H](Cc1ccccc1)C([O-])=O	Z4607347236	-3,227
[O-]C(=O)c1cc(no1)-c1ccc(cc1)-c1cccc2C(=O)NCc12	Z4623200460	-3,618
[O-]C(=O)[C@H](CCc1ccncc1)NC(=O)NC[C@@H](C(F)F)c1ccccc1	Z4766652624	-3,040
CN(Cc1nc2ccccc2s1)C(=O)[C@@H]1C[C@H]1C([O-])=O	Z491669054	-3,249

[O-]C(=O)CNC(=O)c1ccc(cc1)S(=O)(=O)NCc1ccccn1	Z53037899	-3,347
[O-]C(=O)CCNC(=O)C12C[C@H]3C[C@H](C[C@H](C3)C1)C2	Z57053622	-2,850
CC(C)[C@H](NC(=O)C1CCCCC1)C([O-])=O	Z57228992	-2,822
Cc1ccc2nc(sc2c1)-c1ccc(NC(=O)CCC([O-])=O)cc1	Z57410403	-3,349
C[C@@H](N(C)C(=O)CCCC([O-])=O)c1cc2ccccc2o1	Z608663970	-3,685
[O-]C(=O)C1(CC(=O)Nc2cccc3OCCOc23)CCCC1	Z735912646	-3,770
[O-]C(=O)CCC[S@](=O)c1ccc(Br)cc1	Z802570822	-3,091
NC(=O)C[C@@H](NC(=O)c1ccccc1Cl)C([O-])=O	Z94602483	-3,442
Cc1c(nnn1-c1ccc(cc1)-c1ccccc1)C([O-])=O	Z968905616	-3,531
[O-]C(=O)c1cn(nn1)-c1ccc(cn1)C(F)(F)F	Z975855908	-3,389
CC(C)Oc1cccc(CNC(=O)N[C@@](C)(C([O-])=O)c2ccco2)c1	Z975880280	-2,616
C[C@@H]([N@@H+](C)CC(=O)Nc1c(C)cccc1C)C([O-])=O	Z993898370	-2,721

5. ACE2 assay calibration and *in vitro* studies of selected virtual hits

5.1 Optimization of volume.

Plate type: 3544 Corning, low volume, black clear bottom

Volume: 25/40ul

Concentrations: enzyme and substrate solutions prepared according to the instructions

Assay buffer: provided by the Manufacturer (composition not disclosed)

Temperature: 25 ^{o}C

The calibration and further studies were conducted in accord to the Manufacturer manual.[2] Enzyme solution was added into corresponding plate wells, buffer was added into cells corresponding to negative control. Plate was incubated for 15 min at 25 C. Than Substrate solution was added into all experimental wells. Plate was centrifuged and measurement started in kinetic mode (Exc/Em 320/420nm)

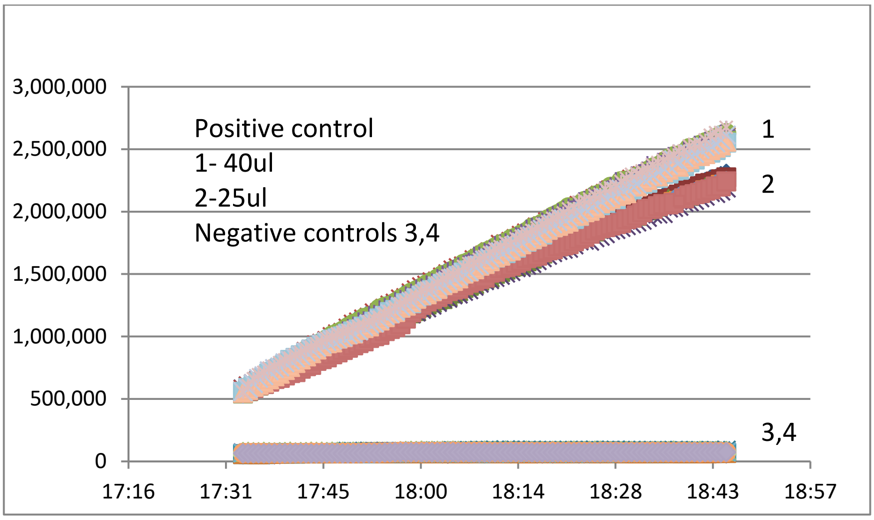

Figure S1. The kinetic curves of positive (inhibitor) control (1 – 40 μL; 2 - 25 μL) and negative (enzyme) control (3 – 40 μL; 4 - 25 μL).

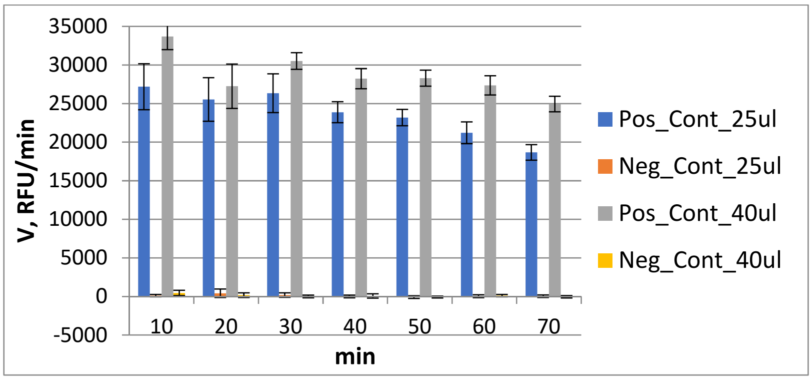

Figure S2. Dependence of relative rate of the reaction (RFU/t) from time of the reaction for positive and negative control for 40 μL and 25 μL.

	Pos_Cont_25ul			Neg_Cont_25ul		Pos_Cont_40ul			Neg_Cont_40ul	
Time, min	Average	SD	CV	Average	SD	Average	SD	CV	Average	SD
10	27186.5	2972.5	10.9	105.6	150.6	33693.1	1705.9	5.1	466.0	342.4
20	25538.0	2822.9	11.1	414.4	549.4	27246.3	2869.6	10.5	163.7	298.0
30	26348.2	2509.5	9.5	177.9	291.1	30522.3	1083.2	3.5	0.5	178.6
40	23878.1	1356.4	5.7	5.1	175.7	28227.1	1303.6	4.6	59.6	280.5
50	23191.4	1062.0	4.6	-79.7	173.5	28295.7	1036.8	3.7	-74.6	126.4

60	21215.8	1402.6	6.6	32.6	186.5	27364.4	1246.4	4.6	105.9	152.7
70	18673.5	1000.4	5.4	34.8	173.5	24943.7	1008.6	4.0	-44.2	157.8

Table S7. The values of standard deviations and coefficient of variations for relative reaction rate (RFU/t) at different volumes and reaction times.

5.2 Optimization of enzyme and substrate dilution.

Plate type: 3544 Corning, low volume, black clear bottom

Volume: 25ul

Concentrations: enzyme and substrate solutions prepared according to the instructions and further diluted '1:1, 1:2, 1:4, 1:8 in Assay Buffer

Assay buffer: provided by the Manufacturer (composition not disclosed)

Temperature: 25°C

Enzyme solutions were added into corresponding plate wells in different concentrations (dilutions), buffer was added into cells corresponding to negative control. Plate was incubated for 15 min at 25 C. Than Substrate solutions were added into all experimental wells at different concentrations (dilutions). Plate was centrifuged and measurement started in **kinetic** mode(**Exc/Em 320/420nm**)

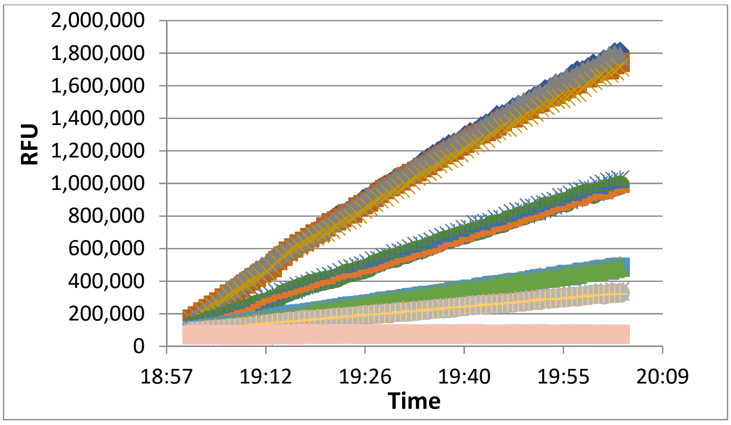

Figure S3. The kinetic curves of positive control and negative control for the undiluted substrate and diluted enzyme (1:1; 1:2; 1:4 and 1:8).

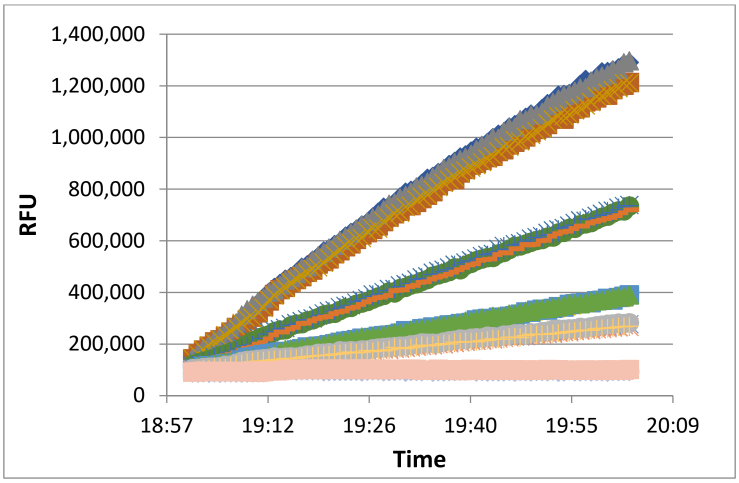

Figure S4. The kinetic curves of positive control and negative control for the 1:2 diluted substrate and diluted enzyme (1:1; 1:2; 1:4 and 1:8).

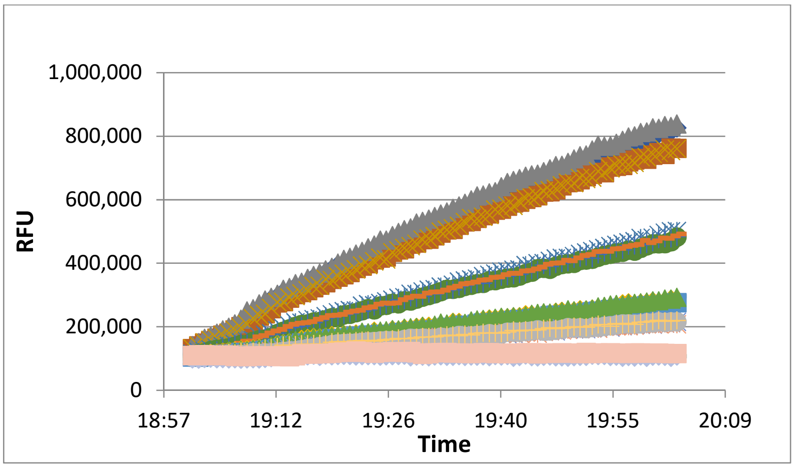

Figure S5. The kinetic curves of positive control and negative control for the 1:4 diluted substrate and diluted enzyme (1:1; 1:2; 1:4 and 1:8).

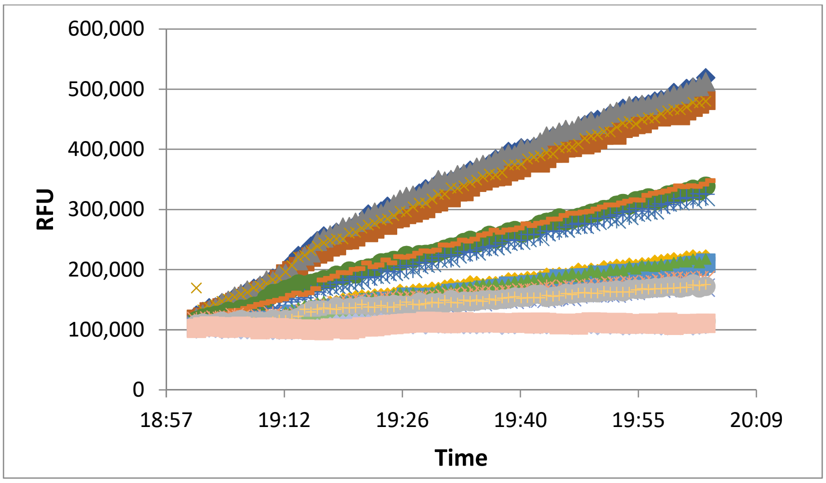

Figure S6. The kinetic curves of positive control and negative control for the 1:8 diluted substrate and diluted enzyme (1:1; 1:2; 1:4 and 1:8).

5.3 IC50 calculations for two reference inhibitors

Two inhibitors were taken as reference: (1) Ref inhibitor provided by the Manufacturer, IC50 33nM for the conditions recommended in manual; (2) Compound from Enamine library EN300-22203532, IC50 440 pM from PubChem. Both reference inhibitors were titrated 3-fold.

Plate type: 3544 Corning, low volume, black clear bottom

Volume: 25ul

Concentrations: enzyme and substrate solutions prepared according to the instructions and further diluted both to **1:2** in Assay Buffer

Assay buffer: provided by the Manufacturer (composition not disclosed)

Inhibitors:

First experiment**:** 3-fold titration in Assay Buffer, 7 concentration points (in nM):

Ref inhibitor(1): 891-297-99-33-11-3.7-1.22

En300-22203532 (2): 13.5-4.5-1.5-0.5-0.167-0.055-0.02

Second experiment**:** 3-fold titration in Assay Buffer, 8 concentration points (in nM):

Ref inhibitor (1): 1500-500-167-55.6-18.5-6.2-2.05-0.7

En300-22203532 (2): 20-6.7-2.2-0.74-0.24-0.08-0.03-0.01

Temperature: 25 °C

Enzyme solution was added into corresponding plate wells in dilution 1:2, buffer was added into wells corresponding to negative control. Both inhibitors were added in aliquot 2.5 ul in 10x concentration into corresponding wells in quadruplicates with 3-fold dilutions. Plate was incubated for 15 min at 25 °C. Then Substrate solution (**1:2**) was added into all experimental wells. Plate was centrifuged and measurement started in **kinetic** mode (**Exc/Em 320/420nm**)

Calculation of results: two time points (**50**th and **60**th minutes) were chosen from kinetic curves for all concentrations and corresponding RFU values were obtained (RFU-50th and RFU-60th

ΔRFU were calculated for each curve and IC50 values for both inhibitors were calculated in GraphPadPrizm 6.

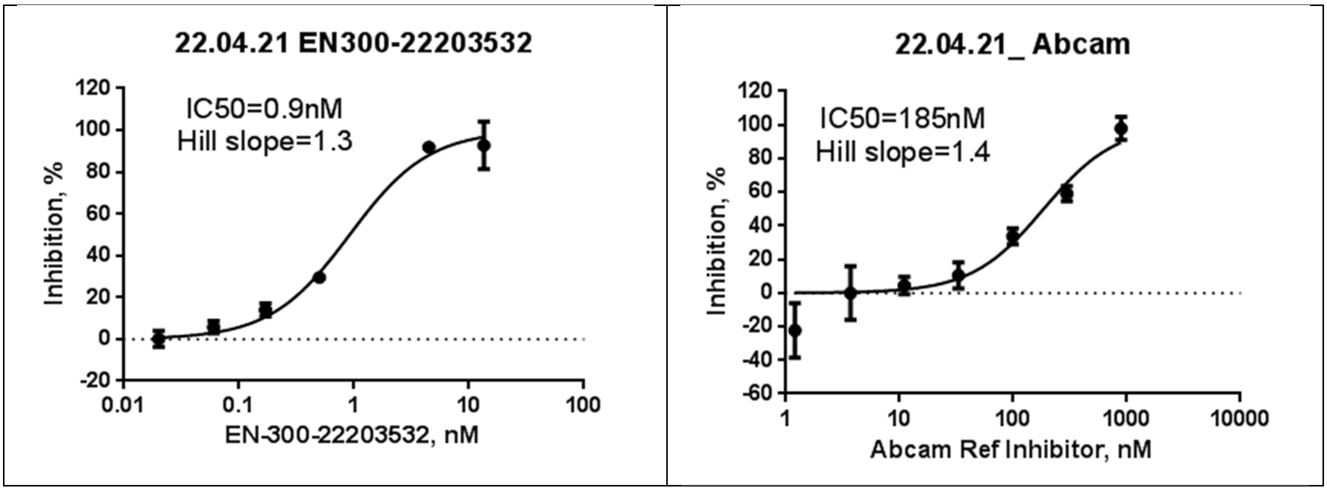

Figure S7. DRC for two reference compounds (MLN-4760 and Abcam ref) in optimized conditions.

5.4 Primary screen QC and results

Parameter	Plate 1	Plate 2
**Z"**	0.84	0.82
**S/B**	5.54	5.2
**%inh_Ref Abcam**	37.5	17.9
**SD_%inh Ref_Abcam**	7.2	7.4
**%inh_Ref EN300-22203532**	69.9	72.2
**SD_%inh Ref_EN300-22203532**	4.8	3.7

Table S8. The QC parameters values of two plates used in screening.

Plate	Well	Compound ID	Inhibition%
**1**	E21	Z57410403	74.1
**2**	J08	Z1459912954	54
**1**	P08	Z3488516360	52.1
**1**	D07	Z2301748685	50.1
**1**	C13	Z2504126907	38.8
**2**	C07	Z95185503	29.8
**2**	I06	Z85905794	28.9

Table S9. Hits selected according to hit criteria Inh% > 3*SD+Avg_plate.

6. Evaluation of the BBB penetration ability of the hit molecules

A boiled-egg diagram[3] representation of computed ADME parameters was used. Compounds **1**, **4** and reference MLN-4760 (marked as **DiCl** on the **Figure S.6.1.**), were predicted to be P-glycoprotein substrates (PGP+) and could be actively pumped up from the brain or to the gastrointestinal lumen, are colored with blue. The prediction was performed using

SwissADME, which is a free web tool for evaluating pharmacokinetics, drug-likeness and medicinal chemistry friendliness of small molecules.[4]

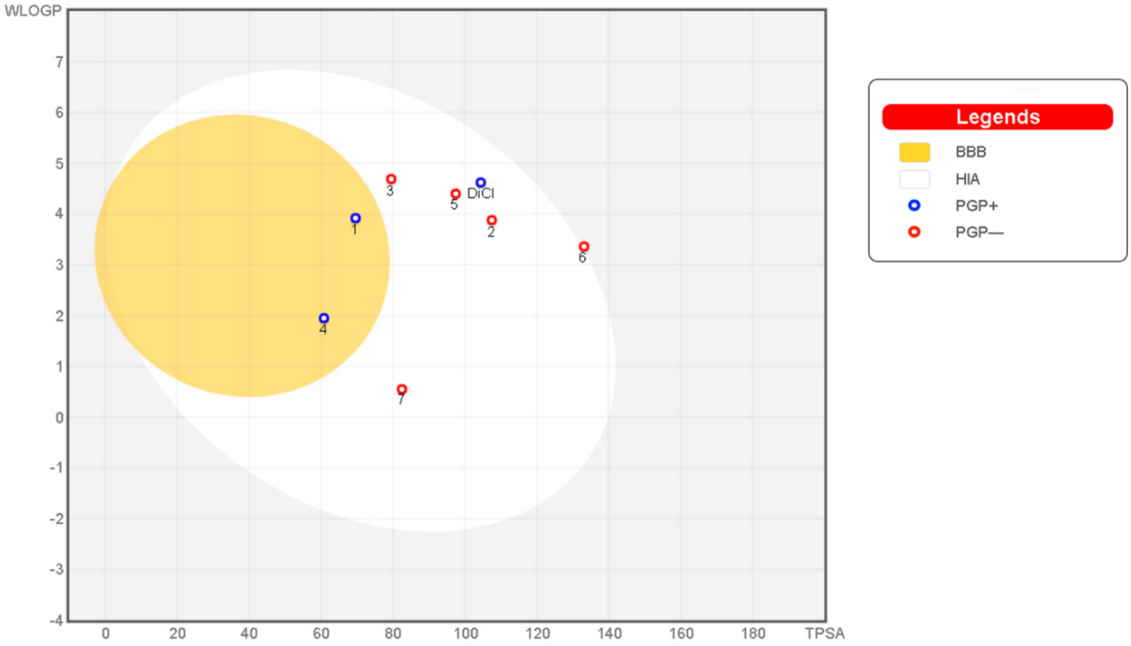

Figure S8. A boiled-egg diagram for the hit compounds (**1-7**) and reference molecule MLN-4760 (**DiCl**).

7. References:

1. Kravets, I.O., et al., *Virtual Screening in Search for a Chemical Probe for Angiotensin-Converting Enzyme 2 (ACE2).* Molecules, 2021. **26**(24).
2. Available from: https://www.abcam.com/angiotensin-ii-converting-enzyme-ace2-inhibitor-screening-kit-ab273373.html.
3. Daina, A. and V. Zoete, *A BOILED-Egg To Predict Gastrointestinal Absorption and Brain Penetration of Small Molecules.* ChemMedChem, 2016. **11**(11): p. 1117-21.
4. Daina, A., O. Michielin, and V. Zoete, *SwissADME: a free web tool to evaluate pharmacokinetics, drug-likeness and medicinal chemistry friendliness of small molecules.* Sci Rep, 2017. **7**: p. 42717.
